# The O-Antigen Epitope Governs Susceptibility to Colistin in Salmonella enterica

**DOI:** 10.1128/mBio.02831-19

**Published:** 2020-01-28

**Authors:** Vito Ricci, Dexian Zhang, Christopher Teale, Laura J. V. Piddock

**Affiliations:** a Antimicrobials Research Group, Institute of Microbiology and Infection, College of Medical and Dental Science, University of Birmingham, Birmingham, United Kingdom; b Animal and Plant Health Agency, Weybridge, New Haw, Addlestone, Surrey, United Kingdom; c Key Laboratory of Zoonosis of Liaoning Province, College of Animal Husbandry and Veterinary Medicine, Shenyang Agricultural University, Shenyang, People’s Republic of China; Louis Stokes Veterans Affairs Medical Center

**Keywords:** whole-genome sequencing, LPS, *Salmonella* Enteritidis, lipopolysaccharide

## Abstract

Group D and group B Salmonella enterica serovars differ in their susceptibility to colistin with the former frequently intrinsically resistant (MIC > 2 μg/ml); however, the mechanism has not been described. Here, we show that the O-antigen epitope in group D *Salmonella* governs the levels of colistin susceptibility. Substitution of the *rfbJ* gene in a group B *Salmonella* with the *rfbSE* genes from a group D *Salmonella* conferred a decrease in susceptibility to colistin. The presence of dideoxyhexose, abequose, and the deoxymannose, tyvelose, differentiate the *Salmonella* group B and group D O antigens, respectively. We hypothesize that the subtle difference between abequose and tyvelose hinders the colistin molecule from reaching its target. Whole-genome sequencing also revealed that increased colistin susceptibility in a group D *Salmonella* veterinary isolate was due to a defect in the O-antigen polymerase protein, Rfc. This study shows that two different mechanisms that influence the presence and composition of O antigens affect colistin susceptibility in Salmonella enterica.

## Abstract (precis)

Some serovars of *Salmonella*, namely, those belonging to group D, appear to show a degree of intrinsic resistance to colistin. This observed intrinsic colistin resistance is of concern since this last-resort drug might no longer be effective for treating severe human infections with the most common *Salmonella* serovar, Salmonella enterica serovar Enteritidis. Here, we show that the O-antigen epitope in group D *Salmonella* governs the levels of colistin susceptibility. Using whole-genome sequencing, we also revealed that increased colistin susceptibility in a group D *Salmonella* veterinary isolate was due to a defect in the O-antigen polymerase protein, Rfc. In summary, we show that two different mechanisms that influence the presence and composition of O antigens affect colistin susceptibility in Salmonella enterica.

## INTRODUCTION

The chromosomal mechanism of evolved colistin resistance in the *Enterobacteriaceae* family involves mutations in several genes conferring structural modifications of the lipopolysaccharide (LPS) (1). LPS modification by cationic substitution has been shown to be mediated by mutations in genes conferring nonsynonymous substitutions in the PmrAB and PhoPQ two-component systems (TCSs) and their regulator MgrB and intermediate PmrD (1). Mutations within the *pmrA* and *pmrB* or *phoP* and *phoQ* genes give rise to constitutive activation of the PmrAB and PhoPQ TCSs, respectively, and lead to upregulation of the *pmrCAB* and *pmrHFIJKLM* operons. This gives increased synthesis of phosphoethanolamine (pEtN) and 4-amino-4-deoxy-l-arabinose (l-Ara4N) and their addition to LPS, thus reducing the efficacy of colistin and polymyxin B against isolates harboring mutations in these genes (MICs typically 4 to 32 μg/ml).

Plasmid-borne *mcr* (mobilized colistin resistance) genes encoding phosphoethanolamine transferases that modify lipid A and confer decreased susceptibility to colistin have been described. To date, nine *mcr* genes have been discovered, *mcr-1* to *mcr-9*, in a variety of members of the family *Enterobacteriaceae*, including Escherichia coli, Klebsiella pneumoniae, and *Salmonella* spp. (2–10). The MICs of colistin in *mcr*-containing strains are typically 2 to 16 μg/ml.

As well as evolved and acquired resistance to colistin, there are several species of Gram-negative bacteria that are naturally resistant to colistin; these include *Proteus* spp., *Serratia* spp., Edwardsiella tarda, and Burkholderia cepacia complex (11–14). The natural resistance to colistin in these species is due to LPS that has been modified with l-Ara4N by various mechanisms, one example being the overexpression of the *arnBCADTEF* operon as observed in *Serratia* spp. (15, 16).

In 2012, Agerso et al. (17) determined the colistin MIC population distribution for Salmonella enterica at the serovar level. Salmonella enterica serovar Dublin and S. enterica serovar Enteritidis were less susceptible than other *Salmonella* serovars originating from humans and S. enterica serovar Typhimurium of animal/meat origin. With Salmonella Dublin and Salmonella Enteritidis belonging to the same O group (O:1,9,12), Agerso et al. suggested that the surface LPS, O antigen, contributes to colistin susceptibility. Analysis of the *pmrA* and *pmrB* DNA sequences of the isolates showed identical sequences for all except in *pmrA* in one isolate of a group B *Salmonella* and *pmrB* in one isolate of a group C1 *Salmonella*; the MICs of colistin for both strains were <1 μg/ml, in which missense mutations were observed. Agerso et al. concluded that increased MICs for colistin were associated with specific serovars, particularly *S*. Dublin and *S.* Enteritidis among the isolates studied, and recommended that *Salmonella* inhibited by MICs of >2 μg/ml for colistin be evaluated at the serovar level. More recently, a report by the European Food Safety Authority (EFSA) and ECDC (European Centre for Disease Prevention and Control) (18) reported data for 2016 on antimicrobial resistance in zoonotic and indicator bacteria, submitted by 28 EU Member States. This report showed that colistin MICs of >2 μg/ml were observed for *Salmonella* serovars belonging to group D, which possess O:9 somatic antigens. The report highlighted that a large proportion of the colistin resistance in *Salmonella* in chickens appeared to be related to the occurrence of *S.* Enteritidis and the higher level of intrinsic resistance of this serovar. As the European Committee on Antimicrobial Susceptibility Testing (EUCAST) recommended breakpoint concentration of 2 μg/ml is the same concentration as the epidemiological cutoff values applied in the analysis, the observed colistin resistance is of concern, since colistin might not be effective for treating severe human infections with the most common *Salmonella* serovar.

The outer leaflet of the outer membrane of Gram-negative bacteria is composed mainly of lipopolysaccharide, which consists of lipid A, core oligosaccharide, and repetitive oligosaccharide units (O antigen), normally containing two to eight sugar residues (19). The O-antigen carbohydrate chain differs between species and is responsible for the serological specificity of bacteria. In *S.* Enteritidis and *S.* Typhimurium, the O antigens are structurally similar, with both serovars sharing identical trisaccharide backbones of α-d-Man*p*-(1-4)-α-l-Rha*p*-(1-3)-α-d-Gal*p*-(1-2), with the mannose bearing tyvelose in *S.* Enteritidis and abequose in *S.* Typhimurium (19–21).

The primary objectives of this study were to determine the mechanism of decreased susceptibility to colistin in *S.* Enteritidis and *S.* Dublin isolates and the role that the O-antigen epitope of group D *Salmonella* has in determining colistin susceptibility.

## RESULTS

### Antimicrobial susceptibilities.

Colistin MIC values of 5.5 to 6 μg/ml were observed for group D *Salmonella*, *S.* Enteritidis NCTC 13349, *S*. Dublin CT_02021853, and for the Animal and Plant Health Agency (APHA) isolates, *S*. Dublin L00668-14, *S.* Enteritidis S02454-14, and *S.* Enteritidis S02576-14, respectively (Table 1). Compared to *S.* Enteritidis NCTC 13349, APHA isolate *S.* Enteritidis S02703-14 was more susceptible to colistin (MIC = 0.75 μg/ml). Compared to *S.* Typhimurium SL1344, APHA isolates *S*. Dublin L00668-14, *S.* Enteritidis S02454-14, and *S.* Enteritidis S02576-14 all had decreased susceptibility to colistin, with MICs of 5.5 μg/ml (Table 1).

**TABLE 1:** Colistin susceptibility and serotyping of the isolates, mutants, and control strains used in this study

Isolate, mutant, or strain^*c*^	Group	Origin, source, or reference^*a*^	Colistin MIC (μg/ml)	Slide agglutination result^*b*^	
				O4 antisera	O9 antisera
Escherichia coli ATCC 25922		PHE culture collections, UK	0.35	NA	NA
*Salmonella* Typhimurium SL1344	B	34	0.85	+++	−
Salmonella Enteritidis NCTC 13349	D	PHE culture collections, UK	5.5	−	+++
Salmonella Dublin CT_02021853	D	PHE culture collections, UK	6	−	+++
Salmonella Dublin L00668-14	D	APHA, Surrey, UK	5.5	−	+++
Salmonella Enteritidis S02454-14	D	APHA, Surrey, UK	5.5	−	+++
Salmonella Enteritidis S02576-14	D	APHA, Surrey, UK	5.5	−	+++
Salmonella Enteritidis S02703-14	D	APHA, Surrey, UK	0.75	−	+
*S*. Typhimurium SL1344 [*rfbSE* (SE) crossover mutant]	D	This study	3.5	−	++
*S*. Typhimurium SL1344 [*rfbSE* (SD) crossover mutant]	D	This study	3	−	++
*S*. Enteritidis NCTC 13349 [*rfbJ* (ST) crossover mutant]	B	This study	1.5	++	−
*S*. Dublin CT_02021853 [*rfbJ* (ST) crossover mutant]	B	This study	1.5	++	−
*S*. Enteritidis NCTC 13349 (*rfc* frameshift mutant)	D	This study	0.8	−	+
*S*. Enteritidis NCTC 13349 (*rfc* frameshift mutant + pWSK30*rfc*^WT^)	D	This study	6	−	+++
Salmonella Enteritidis S02703-14 (+ pWSK30*rfc*^WT^)	D	This study	6	−	+++

a PHE, Public Health England; APHA, Animal and Plant Health Agency.

b NA, not available; +++, very good agglutination; ++, good agglutination; +, poor agglutination; −, no agglutination.

c SE, *S*. Enteritidis; SD, *S*. Dublin; ST, *S*. Typhimurium.

### Whole-genome sequencing reveals serovar-specific differences are responsible for decreased colistin susceptibility, not specific gene mutations.

Analysis of the genomes of the three APHA isolates, *S*. Dublin L00668-14, *S.* Enteritidis S02454-14, and *S.* Enteritidis S02576-14, using Resfinder revealed two different plasmid replicons, IncFII(S) and IncFIB(S), in both *S.* Enteritidis S02454-14 and *S.* Enteritidis S02576-14. IncFII(S) and IncX1 were detected in *S.* Dublin (L00668-14). No resistance genes were detected on these plasmids (see Table S1 in the supplemental material). None of the three isolates contained plasmids that harbored the *mcr-1*, *mcr-2*, *mcr-3*, *mcr-4*, or *mcr-5* gene. The absence of *mcr* alleles *1* to *5* was also verified by PCR.

10.1128/mBio.02831-19.1 TABLE S1 Summary of isolate type, presence of transmissible antibiotic resistance genes, and presence of mutations on chromosomal genes in which mutations can confer resistance to colistin. Download Table S1, DOCX file, 0.02 MB. Copyright © 2020 Ricci et al. 2020 Ricci et al. This content is distributed under the terms of the Creative Commons Attribution 4.0 International license.

No differences were detected in the genes in which mutations can confer resistance to polymyxins and those related to LPS synthesis in the genomes of *S*. Dublin L00668-14, *S.* Enteritidis S02454-14, and *S.* Enteritidis S02576-14 isolates compared to their homologous serovars. However, compared to a nonhomologous serovar (*S.* Typhimurium, isolate SL1344), numerous nonsynonymous single nucleotide polymorphisms (SNPs) were observed in *pmrB*, *pmrC*, *arnB*, *arnC*, *arnA*, *arnD*, *arnT*, *arnE*, *arnF*, *rfbB*, *rfbD*, *rfbA*, *rfbC*, *rfbI*, *rfbF*, *rfbG*, *rfaC*, *rfaF*, *rfaD*, and *rfaL*, all genes in which mutations can confer polymyxin resistance (Table S1). To check whether the observed SNPs in these genes were isolate specific or serovar specific, we ran an *in silico* search, using “snippy,” against approximately 3,000 genomes of *S.* Enteritidis and 200 genomes of *S.* Dublin, obtained from The European Bioinformatics Institute (EMBL-EBI) (https://www.ebi.ac.uk) (accession numbers in Table S2). The *in silico* screen showed that the SNPs observed from our genome sequence data were also detected in all of the *S.* Enteritidis and *S*. Dublin genomes screened. Therefore, we concluded that the nucleotide variants observed in theses serotypes are specific to these serotypes and not linked to colistin susceptibility differences.

10.1128/mBio.02831-19.2 TABLE S2 Accession numbers for *S.* Enteritidis and *S*. Dublin genome sequences used for *in silico* screen. Download Table S2, DOCX file, 0.04 MB. Copyright © 2020 Ricci et al. 2020 Ricci et al. This content is distributed under the terms of the Creative Commons Attribution 4.0 International license.

### Serotype conversion from O4 to O9 and O9 to O4 serotype has an effect on colistin susceptibility.

**FIG 1:**
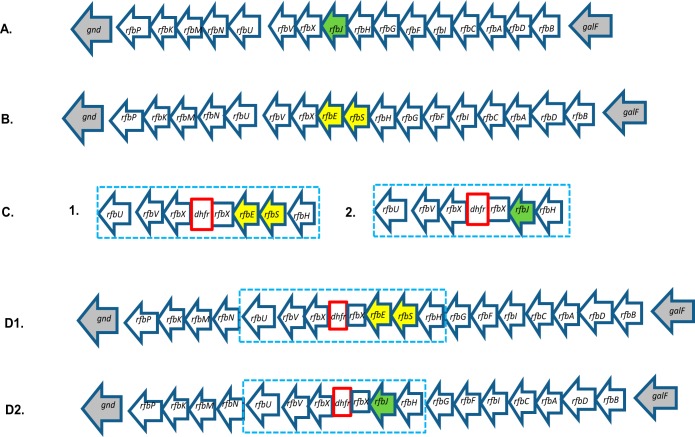
The O-antigen gene clusters of group B and group D *Salmonella* and the strategy taken to make the crossover mutant. (A) The O-antigen gene cluster of *S.* Typhimurium with the *rfbJ* gene highlighted in green. The *rfbJ* gene encodes the enzyme CDP-abequose synthase, which synthesizes abequose. Abequose is the last in a repeat unit of four sugars, which make up the O antigen in group B *Salmonella* and is the sugar that confers the O4 antigen. (B) The O-antigen gene cluster of *S.* Enteritidis and *S*. Dublin with the *rfbSE* genes highlighted in yellow. The *rfbSE* genes encode the enzymes CDP-paratose synthase and CDP-paratose-2-epimerase, respectively. CDP-paratose synthase synthesizes paratose from its precursor, CDP-3,6-dideoxy-α-d-mannose, and CDP-paratose-2-epimerase catalyzes the isomerization of CDP-paratose to CDP-tyvelose. Tyvelose is the last in a repeat unit of four sugars, which make up the O antigen in group D *Salmonella*, and is the sugar that confers the O9 antigen. (C1) Synthesized mutation cassette containing a trimethoprim resistance gene (*dhfr*) (red box) flanked at the 5′ end by DNA sequence homologous to *rfbUVX* and at the 3′ end by DNA sequence homologous to *rfbXESH.* (C2) Synthesized mutation cassette containing a trimethoprim resistance gene (*dhfr*) (red box) flanked at the 5′ end by DNA sequence homologous to *rfbUVX* and at the 3′ end by DNA sequence homologous to *rfbXJH.* (D1) The synthesized mutation cassette (blue dashed rectangular box) recombined into the chromosome of *S*. Typhimurium, where the *rfbJ* gene has been replaced by the *rfbSE* genes that are highlighted in yellow. (D2) The synthesized mutation cassette (blue dashed rectangular box) recombined into the chromosome of *S.* Enteritidis and *S*. Dublin, where the *rfbSE* genes have been replaced by the *rfbJ* gene that is highlighted in green. Excision of the trimethoprim gene (*dhfr)* (red box) from the chromosome was conducted by using pACBSCE.

**FIG 2:**
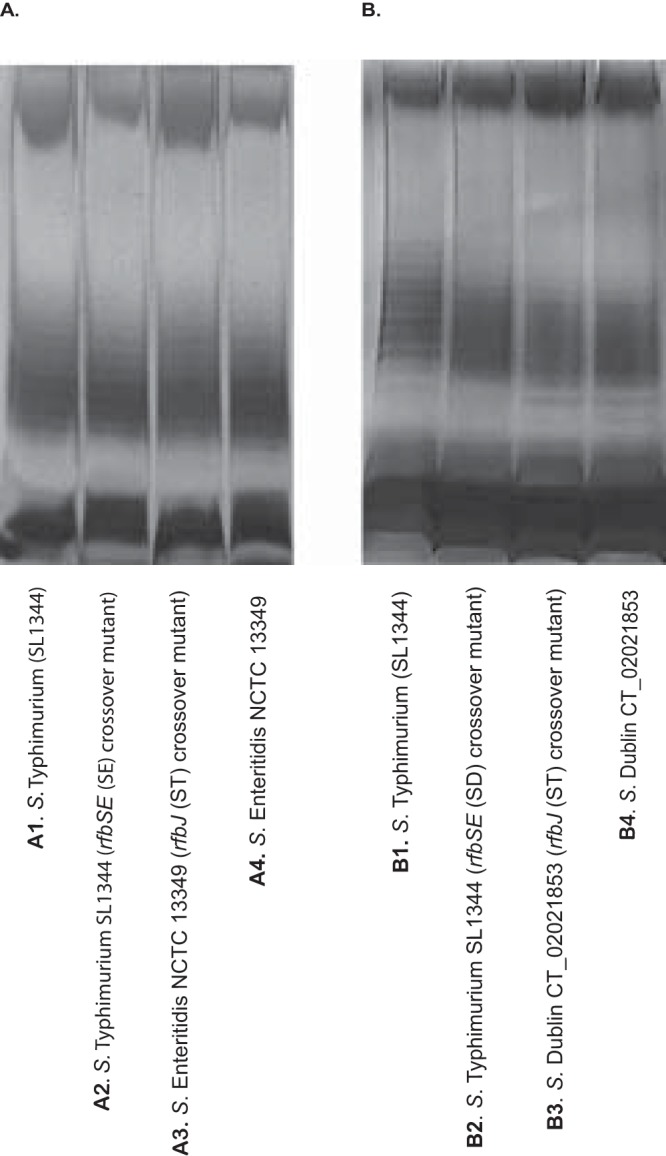
Lipopolysaccharide (LPS) profiles of wild-type strains and *rfbSE* and *rfbJ* crossover mutants as visualized by silver nitrate staining.

In Salmonella enterica, the genes involved in the biosynthesis of the basic O antigen are located in a specific locus between the *galF* and *gnd* genes. The O-antigen unit is synthesized by sequential transfer of individual sugars from their respective dinucleotide precursors to the carrier lipid, undecaprenyl pyrophosphate (UndPP). These reactions are catalyzed by specific sugar transferases, and the last sugar in the chain confers the O-antigenic epitope characteristic of the group. In *Salmonella* group D serovars, the *rfbSE* genes encode the enzymes CDP-paratose synthase and CDP-paratose-2-epimerase, respectively. CDP-paratose synthase synthesizes paratose from its precursor, CDP-3,6-dideoxy-α-d-mannose, and CDP-paratose-2-epimerase catalyzes the isomerization of CDP-paratose to CDP-tyvelose. Tyvelose is the last in a repeat unit of four sugars, which make up the O antigen in group D *Salmonella* and is the sugar that confers the O9 antigen. In *Salmonella* group B serovars, the *rfbJ* gene encodes the enzyme CDP-abequose synthase, which synthesizes abequose from its precursor, CDP-4-dehydro-3,6-dideoxy-α-d-glucose. Abequose is the last in a repeat unit of four sugars, which make up the O antigen in group B *Salmonella*, and is the sugar that confers the O4 antigen (19–21). In order to test whether serovar conversion from O4 to O9 and O9 to O4 serovar, respectively, affects colistin susceptibility, we replaced the *rfbJ* gene in *S.* Typhimurium (SL1344) on the chromosome with the *rfbSE* genes from *S.* Enteritidis (NCTC 13349) and *S*. Dublin (CT_02021853). We also replaced the *rfbSE* genes on the chromosomes of *S.* Enteritidis (NCTC 13349) and *S*. Dublin (CT_02021853) with the *rfbJ* gene from *S.* Typhimurium (SL1344) (Fig. 1). To establish that the LPS pathway had not been disrupted during the construction of the *rfbSE* and *rfbJ* crossover mutants, we analyzed lipopolysaccharide profiles by silver staining. Silver-stained LPS profiles of *S.* Typhimurium (SL1344), *S.* Enteritidis (NCTC 13349), *S*. Dublin (CT_02021853), and the *rfbSE* and *rfbJ* crossover mutants showed that all gave a smooth LPS profile, and band patterns were similar, suggesting that the biosynthetic pathway had not been disrupted (Fig. 2). Serotyping of the *rfbSE* crossover mutants by slide agglutination confirmed successful serovar conversion from O4 to O9 with good agglutination with O9 antisera and no agglutination with O4 antisera, whereas their parent strains agglutinated only with O4 antisera (Table 1). Serotyping of the *rfbJ* crossover mutants by slide agglutination confirmed successful serovar conversion from O9 to O4 with good agglutination with O4 antisera and no agglutination with O9 antisera, whereas their parent strains agglutinated only with O9 antisera (Table 1).

Compared to the parental strain, *S*. Typhimurium SL1344, both *rfbSE* (*S*. Enteritidis) and *rfbSE* (*S*. Dublin) crossover mutants had decreased susceptibility to colistin (MIC of 3/3.5 μg/ml versus MIC of 0.85 μg/ml; Table 1). Compared to the parent strains *S.* Enteritidis NCTC 13349 and *S*. Dublin CT_02021853, the respective *rfbJ* crossover mutants had an increased susceptibility to colistin (MIC of 1.5 μg/ml versus MIC of 5.5/6 μg/ml; Table 1).

### Defects in O-antigen polymerase can increase colistin susceptibility in group D *Salmonella*.

**FIG 3:**
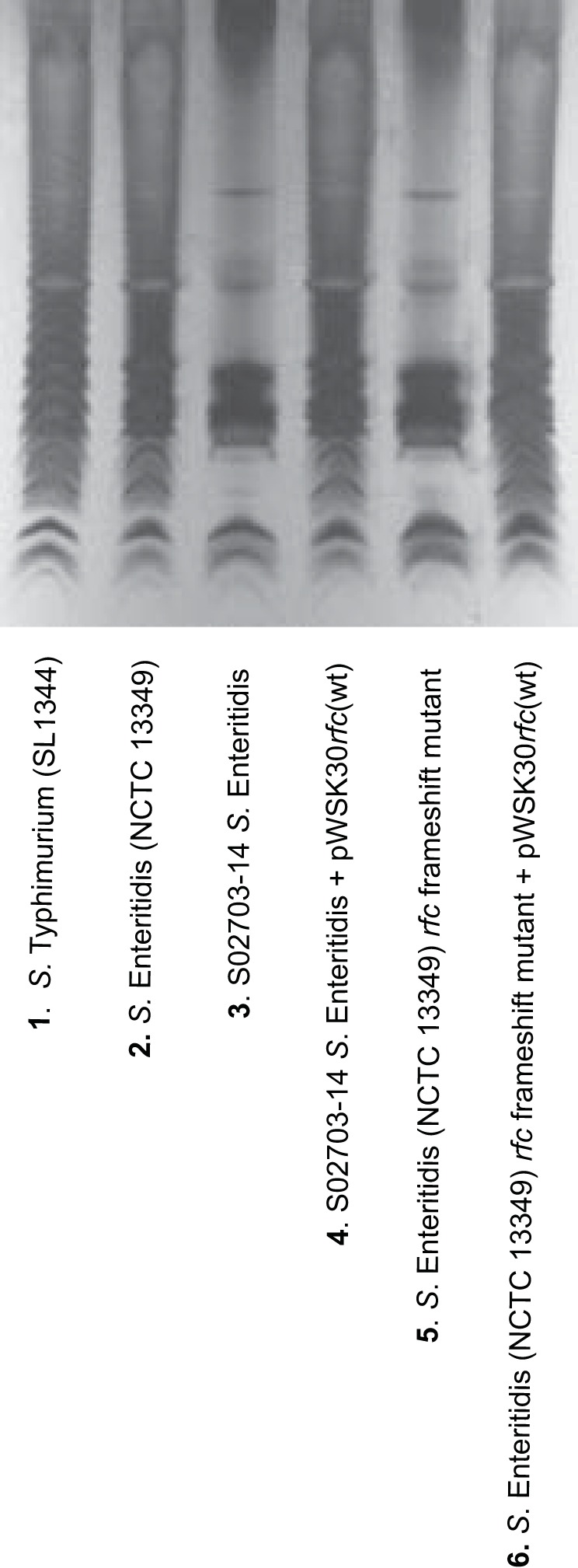
LPS profiles showing the effect of the *rfc* frameshift mutation on O-antigen production and the effect of complementing the colistin-susceptible *S.* Enteritidis isolate S02703-14 and the *rfc* frameshift mutant with wild-type (wt) *rfc* on pWSK30.

To ascertain whether the natural resistance to colistin exhibited by group D *Salmonella* is O antigen specific or whether other mechanisms were involved, we sequenced the genome of a colistin-susceptible *S.* Enteritidis (S02703-14) isolated from a chicken. Isolate S02703-14 was more susceptible to colistin (MIC = 0.75 μg/ml) than was the *S.* Enteritidis NCTC 13349 reference strain (MIC = 5.5 μg/ml). Following whole-genome sequencing of isolate S02703-14 and comparison with the genome of *S.* Enteritidis (NCTC 13349), five nonsynonomous single nucleotide polymorphisms, one nonsynonomous multinucleotide polymorphism (MNP), and one deletion mutation were found (Table S3). The deletion mutation occurred in the *rfc* gene which encodes the O-antigen polymerase Rfc, responsible for linking the O-antigen tetrasaccharide units into long chains (21). The deletion occurred at position 1818500 on the chromosome of isolate S02703-14 where deletion of a cytosine (C) in the codon for serine (TCT) at amino acid position 152 in the Rfc protein caused a frameshift (TCT → TT = S152fs). The deletion mutation was confirmed by PCR and DNA sequencing. To establish the role of the deletion mutation found in *rfc*, upon susceptibility to colistin, the observed nucleotide deletion (TCT → TT) was introduced onto the chromosome of *S.* Enteritidis (NCTC 13349) by site-directed mutagenesis. The introduction of this mutation increased the susceptibility of colistin in the *rfc* frameshift mutant, compared to the parent strain *S.* Enteritidis NCTC 13349, from 5.5 to 0.8 μg/ml. Poor agglutination was observed with O9 antisera (Table 1), and the LPS profile of the *rfc* frameshift mutant exhibited a semirough appearance (Fig. 3) with a lack of long-chain O-antigen bands observed. To determine whether the *rfc* frameshift mutation was responsible for the observed phenotype, we complemented both the *rfc* frameshift mutant and the original colistin-sensitive *S.* Enteritidis isolate S02703-14, with wild-type *rfc* cloned into a pWSK30 plasmid. In both complemented strains, the susceptibility to colistin decreased from 0.75 and 0.8 μg/ml, respectively, to 6 μg/ml (Table 1), and good agglutination was observed with O9 antisera. The LPS profiles reverted to those observed for the parental strains (Table 1 and Fig. 3).

10.1128/mBio.02831-19.3 TABLE S3 Mutations identified in the genome-sequenced Salmonella Enteritidis S02703-14 isolate. Download Table S3, DOCX file, 0.01 MB. Copyright © 2020 Ricci et al. 2020 Ricci et al. This content is distributed under the terms of the Creative Commons Attribution 4.0 International license.

## DISCUSSION

In the majority of bacterial species studied, natural resistance to polymyxins has been linked to the constitutive expression of the *arnBCADTEF* operon and/or the *eptB* gene. Overexpression of these genes causes the addition of phosphoethanolamine (pEtN) and/or 4-amino-4-deoxy-l-arabinose (l-Ara4N) cationic groups to the LPS, which increases the charge of the LPS and subsequently decreases polymyxin binding (22–24). Gram-negative bacteria that are known to be naturally resistant to polymyxins include *Proteus* spp., *Providencia* spp., Morganella morganii, *Serratia* spp., Edwardsiella tarda, and Burkholderia cepacia complex.

To date, the mechanism by which certain serovars of Salmonella enterica are intrinsically resistant to colistin has not been described. In this study, we show that intrinsic colistin resistance in two group D *Salmonella* serovars is attributable to the O-antigen epitope governing their antigenic specificity. The genes involved in O-antigen biosynthesis are located in the *rfb* gene cluster (25). The difference between the O-antigen structures of the group D and B *Salmonella* is the presence of CDP-abequose synthase in group B versus CDP-paratose synthase in group D plus an additional enzyme, CDP-paratose-2-epimerase, which converts CDP-paratose to CDP-tyvelose (26). The CDP-abequose synthase in group B is encoded by the *rfbJ* gene, and the CDP-paratose synthase and CDP-tyvelose are encoded by *rfbS* and *rfbE*, respectively, in group D serovars (26). In this study, we replaced the *rfbJ* gene in *S.* Typhimurium (SL1344), a group B *Salmonella*, with the *rfbS* and *rfbE* genes from two group D *Salmonella* serovars, *S.* Enteritidis and *S.* Dublin, and showed that this conferred colistin resistance to the group B *Salmonella*. Our data showed a decrease in susceptibility to colistin in both mutants and a change in serotype from an O4 serotype to an O9 serotype with no evidence of any other interference in LPS production as evidenced by the LPS profiles. We also conducted the reciprocal experiments and replaced the *rfbSE* genes in two group D *Salmonella* serovars, *S.* Enteritidis and *S.* Dublin, with the *rfbJ* gene from *S.* Typhimurium (SL1344). From these reciprocal experiments, our data showed an increase in susceptibility to colistin in both mutants and a change in serotype from an O9 serotype to an O4 serotype with no evidence of any other interference in LPS production as evidenced by the LPS profiles.

We also show that increased susceptibility to colistin in a group D *Salmonella* serovar, *S.* Enteritidis, was due to a defect in the O-antigen polymerase. Whole-genome sequencing of a colistin-sensitive *S.* Enteritidis isolate revealed a frameshift mutation in the *rfc* gene which encodes the O-antigen polymerase. The O-antigen polymerase (Rfc) is a membrane protein that is responsible for extension of the O antigen by the addition of repeating units (27). We hypothesize that the truncation of the Rfc protein caused by the frameshift mutation will lead to inefficient assembly and polymerization of the O-antigen subunits, therefore leading to a rough LPS profile and a more permeable cell membrane. To test this hypothesis we constructed the frameshift mutation in *rfc* on the chromosome of a colistin-resistant *S.* Enteritidis strain. Our data showed that the *S.* Enteritidis mutant was more susceptible to colistin, reflecting the original colistin-susceptible *S.* Enteritidis isolate S02703-14. The rough LPS profile exhibited by the *rfc* frameshift mutant compared to the type strain suggests that a more complex molecular mechanism may be involved. However, the whole-genome sequence data for the original isolate carrying the *rfc* frameshift mutation (*S.* Enteritidis S02703-14) does not indicate any other changes, and data from the *rfc* complement experiments suggest that the *rfc* mutation confers the rough LPS effect observed.

In this study, we have shown that the O-antigen epitope, whether it be due to inefficient assembly or substitution for another type, can govern the level of susceptibility to colistin in S. enterica. The immune-dominant sugars in the O antigens of *Salmonella* group B and group D strains are abequose and tyvelose, respectively (26). Although very similar in structure, differing only in the position of a hydroxyl group, the chemistry of the O antigen is an important factor. We hypothesize that this subtle difference between abequose and tyvelose is the cause of the reduced susceptibility to colistin observed in the strains and isolates in our study. One explanation is that tyvelose may hinder the colistin molecule from reaching its target, as the LPS component of the bacterial outer membrane is the initial cellular target of polymyxins (28).

Recent reports and studies have shown that higher levels of resistance to colistin were observed for *S.* Enteritidis than for other *Salmonella* serovars (17, 18). This is of concern since the last-resort drug colistin might not be effective for treating severe human infections with the most common *Salmonella* serovar. More of a concern would be if this characteristic were to become transmissible and shared between colistin-susceptible serovars.

## MATERIALS AND METHODS

### Bacterial strains.

Four veterinary *Salmonella* group D isolates were obtained from the Animal and Plant Health Agency (APHA), Surrey, United Kingdom (Table 1). All other quality control and reference strains used in this study are described in Table 1. Isolates were stored at 4°C and cultured on cation-adjusted Mueller-Hinton (MH) agar (Oxoid, Basingstoke, Hampshire, UK) and incubated overnight at 37°C.

### Antimicrobial susceptibility testing.

Antimicrobial susceptibility testing of all NCTC, APHA, and constructed mutant strains in this study to colistin (polymyxin E) and other antimicrobials was determined in triplicate by the broth microdilution (BMD) method. EUCAST guidelines (http://www.eucast.org/fileadmin/src/media/PDFs/EUCAST_files/General_documents/Recommendations_for_MIC_determination_of_colistin_March_2016.pdf) were followed conforming to ISO 20776-1:2006 (29), except that decimal dilutions (increments of 0.5 μg/ml) of antimicrobials were used rather than doubling dilutions. E. coli ATCC 25922 was used as the control strain.

### Whole-genome sequencing (WGS), assembly, and annotation.

Whole-genome sequencing was conducted by the Beijing Genomics Institute (BGI), using paired-end sequencing performed on the Illumina HiSeq 4000 platform. DNA extraction from each isolate was conducted using the Bioline Isolate II genomic DNA kit (Bioline, UK). The trimmed read FASTQ files for each isolate were converted using FASTQ Groomer in order to be consistent with Sanger FASTA format. The paired reads were joined by “FASTQ interlacer” using sequence identifiers. The reads were then assembled by *de novo* assembly using Velvet Optimiser. All operations were conducted using a Galaxy installation on CLIMB (Cloud Infrastructure for Microbial Bioinformatics). The reference genomes used were *S.* Typhimurium (SL1344), *S.* Enteritidis (NCTC 13349), and *S*. Dublin (CT_02021853). Annotation was performed using RAST (http://rast.nmpdr.org/rast.cgi).

### Multilocus sequence typing and plasmid and resistance gene identification.

Multilocus sequence typing (MLST) for each isolate was conducted using the MLST finder tool (Centre of Genetic Epidemiology [CGE]; https://cge.cbs.dtu.dk/services/MLST/). Plasmid identification was performed using the Plasmid finder tool (CGE) (https://cge.cbs.dtu.dk/services/PlasmidFinder) which identified the number of plasmids and gave the Inc group and replicon type for each plasmid. Known resistance genes present in each genome were identified using the Resfinder tool (CGE) (https://cge.cbs.dtu.dk/services/ResFinder) and the Comprehensive Antibiotic Resistance Gene Database (CARD) (http://arpcard.mcmaster.ca). Resfinder provided a list of plasmid-mediated resistance genes present in each genome, and CARD showed chromosomal genes with mutations and plasmid-mediated resistance genes. CARD also showed the sequences of all of the resistance genes detected. The sequences of chromosomal genes with mutations were analyzed by alignment of the gene sequence to the reference genome. The Artemis navigator (http://www.sanger.ac.uk/science/tools/artemis) was then used to locate the resistance genes that had been found. The amino acid sequence for each resistance gene in each isolate was aligned to the same gene’s amino acid sequence in the reference genome using Clustal Omega (www.ebi.ac.uk/Tools/msa/clustalo/).

### Single nucleotide polymorphism analysis.

In order to find both substitutions (SNPs) and insertions/deletions (indels) between the reference genome and isolates, we used one command line program, “snippy” (https://github.com/tseemann/snippy), and a web-based program, Resfinder-3.0 (https://cge.cbs.dtu.dk/services/ResFinder-3.0/).

### Screening for mobile colistin resistance (*mcr*) genes.

All isolates in this study were screened for the presence of colistin resistance genes (*mcr-1*, *mcr-2*, *mcr-3*, *mcr-4*, and *mcr-5*) by PCR. Amplification of each gene was conducted using the primers *mcr-1* Fw (Fw stands for forward) (5′-ATGCCAGTTTCTTTCGCGTG-3′) and *mcr-1* Rv (Rv stands for reverse) (5′-TCGGCAAATTGCGCTTTTGGC-3′), *mcr-2* Fw (5′-GATGGCGGTCTATCCTGTAT-3′), *mcr-2* Rv (5′-AAGGCTGACACCCCATGTCAT-3′), *mcr-3* Fw (5′-ACCAGTAAATCTGGTGGCGT-3′) *mcr-3* Rv (5′-AGGACAACCTCGTCATAGCA-3′), *mcr-4* Fw (5′-TTGCAGACGCCCATGGAATA-3′), *mcr-4* Rv (5′-GCCGCATGAGCTAGTATCGT-3′), *mcr-5* Fw (5′-GGACGCGACTCCCTAACTTC-3′), and *mcr-5* Rv (5′-ACAACCAGTACGAGAGCACG-3′). PCR was performed as previously described (30).

### Site-directed mutagenesis.

A modified version of the method described by Kim et al. (31) was used for the construction of the *rfbSE* and *rfbJ* crossover chromosomal mutants and the *rfc* frameshift mutant. For the *rfbSE* and *rfbJ* crossover chromosomal mutants, mutation cassettes were synthesized by Integrated DNA Technologies, Inc. (USA). Each cassette contained a trimethoprim resistance gene (*dhfr*) flanked at the 5′ end by sequence homologous to *rfbUVX* and at the 3′ end by sequence homologous to *rfbXESH* for the *rfbSE* crossover mutant and *rfbXJH* for the *rfbJ* crossover mutant (Fig. 1). The respective mutation cassettes were inserted into the chromosome of *S.* Typhimurium (SL1344), *S.* Enteritidis NCTC 13349, and *S*. Dublin CT_02021853 using pSIM18 hygromycin selection (32). Recombinants were selected by plating onto LB agar with 50 μg/ml of trimethoprim. For the *rfc* frameshift mutation, a mutation cassette was synthesized by Integrated DNA Technologies, Inc. (USA) which contained a kanamycin resistance gene (*aph*) flanked by sequence homologous to *rfc* which contained the nucleotide deletion giving rise to the frameshift at amino acid position 152. Excision of the trimethoprim and kanamycin selection cassettes from the chromosome was performed with pACBSCE (33). Mutant candidates were screened by PCR for loss of the selection marker and sequenced to confirm correct insertion of the desired mutation.

The *rfc* frameshift mutant and colistin-sensitive *S.* Enteritidis (S02703-14) were complemented with a wild-type *rfc* gene (amplified from *S.* Enteritidis NCTC 13349) on a pWSK30 plasmid.

### LPS extraction.

LPS was isolated as follows. *Salmonella* isolates were grown overnight at 37°C, and the following day the equivalent of 1 ml of a culture with an optical density at 600 nm (OD_600_) of 1 was centrifuged and the cell pellet was resuspended in 100 μl of lysing buffer (1 M Tris [pH 6.8], 2% sodium dodecyl sulfate [SDS], and 4% 2-mercaptoethanol). The suspension was then boiled for 10 min and centrifuged, and the supernatant was retained. Proteinase K was added to 0.25 mg/ml, and the sample was incubated at 60°C for 1 h. Finally, the LPS preparation was heated at 98°C for 10 min and stored at −20°C. LPS was resolved on 10% Bis-Tris SDS-polyacrylamide gels and visualized by silver staining using the SilverQuest kit (Thermo Fisher).

### Serotyping.

Serotyping of the *Salmonella* isolates in this study was conducted by slide agglutination assays using monovalent somatic O antiserum group B, factor 4 and group D, factor 9 (Pro-Lab Diagnostics). Two separate loopfuls of normal saline (0.85% sodium chloride) were placed on a clean glass slide. A small part of a *Salmonella* colony from an overnight culture plate was mixed thoroughly with both drops of normal saline on the slide to obtain a smooth suspension. One loopful of antisera was added to one of the bacterial suspension drops on the slide; to the other (control), one loopful of normal saline was added. The antiserum was mixed with the bacterial suspension using a sterile loop. The slide was gently tilted back and forth for 1 min and observed for agglutination under normal lighting conditions.
