# Supplementary material for: The O-Antigen Epitope Governs Susceptibility to Colistin in Salmonella enterica
Source: mBio. 2020 Jan 28;11(1):e02831-19. doi: 10.1128/mBio.02831-19 (PMC6989106; doi:10.1128/mBio.02831-19)
Supplement: TABLE S1 [file mBio.02831-19-st001.docx]

**Supplementary Table 1. Summary of Isolate type, presence of transmissible antibiotic resistance genes and presence of mutations on chromosomal genes in which mutations can confer resistance to colistin.**

| **Source of Isolates** | **APHA** | | | | | | | | |
| --- | --- | --- | --- | --- | --- | --- | --- | --- | --- |
| **Isolate** | *S*. Enteritidis (NCTC 13349) | S02454-14  *S. Enteritidis* | S02576-14  *S. Enteritidis* | *S*. Dublin (CT_02021853) | L00668-14 *S. Dublin* | *S*.Typhimurium SL1344 | S02454-14  *S. Enteritidis* | S02576-14 *S. Enteritidis* | L00668-14  *S. Dublin* |
| **Serotype** |  | O-9:g,m | O-9:g,m | O-9:g,p | O-9:g,p | O-4;i:1,2 | O-9:g,m | O-9:g,m | O-9:g,p |
| **MLST** | ST-11 | ST-11 | ST-11 | ST-10 | ST-10 |  | ST-11 | ST-11 | ST-10 |
| **Number of Plasmids** | 0 | 2 | 2 | 2 | 2 | 3 | 2 | 2 | 2 |
| **Inc Group (Replicon type)** | **-** | *IncFII(S)*  *IncFIB(S)* | *IncFII(S)*  *IncFIB(S)* | *IncFII(S)*  *IncX1* | *IncFII(S)*  *IncX1* | *IncI1*  *IncQ1*  *IncFII(S)* | *IncFII(S)*  *IncFIB(S)* | *IncFII(S)*  *IncFIB(S)* | *IncFII(S)*  *IncX1* |
| **Plasmid Mediated Resistance Genes** | **-** | ND | ND | ND | ND | Present | ND | ND | ND |
| **Chromosomal genes in which mutations can confer resistance** |  | Compared to  *S*. Enteritidis (NCTC 13349) | Compared to  *S*. Enteritidis (NCTC 13349) |  | Compared to  *S*. Dublin (CT_02021853) |  | Compared to  *S*.Typhimurium SL1344 | Compared to  *S*.Typhimurium SL1344 | Compared to  *S*.Typhimurium SL1344 |
| **Polymyxins** |  |  |  |  |  |  |  |  |  |
| *pmrA* | Wild type | Wild type | Wild type | Wild type | Wild type | Wild type | Wild type | Wild type | Wild type |
| *pmrB* | Wild type | Wild type | Wild type | Wild type | Wild type | Wild type | Wild type | Wild type | T9P |
| *pmrC/eptA* | Wild type | Wild type | Wild type | Wild type | Wild type | Wild type | L77P  A159V  G232S | L77P  A159V  G232S | Q494H |
| *pmrD* | Wild type | Wild type | Wild type | Wild type | Wild type | Wild type | Wild type | Wild type | Wild type |
| *arnB* | Wild type | Wild type | Wild type | Wild type | Wild type | Wild type | N96T | N96T | N96T |
| *arnC* | Wild type | Wild type | Wild type | Wild type | Wild type | Wild type | T32A | T32A | T32A |
| *arnA* | Wild type | Wild type | Wild type | Wild type | Wild type | Wild type | Q48L  S195A  D284G | Q48L  S195A  D284G | Q48L  S195A  D284G  D554E |
| *arnD* | Wild type | Wild type | Wild type | Wild type | Wild type | Wild type | S164P  A268T | S164P  A268T | S164P  A268T |
| *arnT* | Wild type | Wild type | Wild type | Wild type | Wild type | Wild type | M160T  K404Q | M160T  K404Q | M160T  K404Q |
| *arnE* | Wild type | Wild type | Wild type | Wild type | Wild type | Wild type | A29V  L95F | A29V  L95F | A29V  L95F |
| *arnF* | Wild type | Wild type | Wild type | Wild type | Wild type | Wild type | F43L  Q65H | F43L  Q65H | F43L  Q65H |
| *rosB* | Wild type | Wild type | Wild type | Wild type | Wild type | Wild type | Wild type | Wild type | Wild type |
| *phoQ* | Wild type | Wild type | Wild type | Wild type | Wild type | Wild type | Wild type | Wild type | Wild type |
| *phoP* | Wild type | Wild type | Wild type | Wild type | Wild type | Wild type | Wild type | Wild type | Wild type |
| **Genes involved in LPS biosynthesis** |  |  |  |  |  |  |  |  |  |
| *rfbB* | Wild type | Wild type | Wild type | Wild type | Wild type | Wild type | A106V | A106V | A106V |
| *rfbD* | Wild type | Wild type | Wild type | Wild type | Wild type | Wild type | N205K | N205K | N205K |
| *rfbA* | Wild type | Wild type | Wild type | Wild type | Wild type | Wild type | H104N  K156S  E211D | H104N  K156S  E211D | H104N  K156S  E211D |
| *rfbC* | Wild type | Wild type | Wild type | Wild type | Wild type | Wild type | A153T  D177H | A153T  D177H | A153T  D177H |
| *rfbI* | Wild type | Wild type | Wild type | Wild type | Wild type | Wild type | Y234D | Y234D | Y234D  S10L |
| *rfbF* | Wild type | Wild type | Wild type | Wild type | Wild type | Wild type | R167Q | R167Q | R167Q |
| *rfbG* | Wild type | Wild type | Wild type | Wild type | Wild type | Wild type | N56S  T116A | N56S  T116A | N56S  T116A |
| *rfbH* | Wild type | Wild type | Wild type | Wild type | Wild type | Wild type | Wild type | Wild type | Wild type |
| *rfbJ* | Not present | Not present | Not present | Not present | Not present | Wild type | Not present | Not present | Not present |
| *rfaC* | Wild type | Wild type | Wild type | Wild type | Wild type | Wild type | A36V  R79H  N85Y  Q302K | A36V  R79H  N85Y  Q302K | A36V  R79H  N85Y  Q302K |
| *rfaF* | Wild type | Wild type | Wild type | Wild type | Wild type | Wild type | Q161L  C171R | Q161L  C171R | Q161L  C171R |
| *rfaD* | Wild type | Wild type | Wild type | Wild type | Wild type | Wild type | G257S | G257S | G257S |
| *rfaL* | Wild type | Wild type | Wild type | Wild type | Wild type | Wild type | V340A  L355L | V340A  L355L | V340A  L355L |

ND, not detected
