# Supplementary material for: The O-Antigen Epitope Governs Susceptibility to Colistin in Salmonella enterica
Source: mBio. 2020 Jan 28;11(1):e02831-19. doi: 10.1128/mBio.02831-19 (PMC6989106; doi:10.1128/mBio.02831-19)
Supplement: TABLE S2 [file mBio.02831-19-st002.docx]

**Supplementary Table 2. Accession numbers for *S*. Enteritidis and *S*. Dublin genome sequences used for *in silico* screen.**

| ***Salmonella enterica subsp. enterica* serovar Enteritidis** | | |
| --- | --- | --- |
| **Accession number** | **Scientific name** | |
| ERR025164 | Salmonella enterica subsp. enterica serovar Enteritidis | |
| ERR025165 | Salmonella enterica subsp. enterica serovar Enteritidis | |
| ERR025166 | Salmonella enterica subsp. enterica serovar Enteritidis | |
| ERR025172 | Salmonella enterica subsp. enterica serovar Enteritidis | |
| ERR025173 | Salmonella enterica subsp. enterica serovar Enteritidis | |
| ERR025174 | Salmonella enterica subsp. enterica serovar Enteritidis | |
| ERR036132 | Salmonella enterica subsp. enterica serovar Enteritidis | |
| ERR036134 | Salmonella enterica subsp. enterica serovar Enteritidis | |
| ERR036135 | Salmonella enterica subsp. enterica serovar Enteritidis | |
| ERR036136 | Salmonella enterica subsp. enterica serovar Enteritidis | |
| ERR036137 | Salmonella enterica subsp. enterica serovar Enteritidis | |
| ERR036138 | Salmonella enterica subsp. enterica serovar Enteritidis | |
| ERR036139 | Salmonella enterica subsp. enterica serovar Enteritidis | |
| ERR036140 | Salmonella enterica subsp. enterica serovar Enteritidis | |
| ERR037559 | Salmonella enterica subsp. enterica serovar Enteritidis | |
| ERR037560 | Salmonella enterica subsp. enterica serovar Enteritidis | |
| ERR037561 | Salmonella enterica subsp. enterica serovar Enteritidis | |
| ERR037562 | Salmonella enterica subsp. enterica serovar Enteritidis | |
| ERR037563 | Salmonella enterica subsp. enterica serovar Enteritidis | |
| ERR037564 | Salmonella enterica subsp. enterica serovar Enteritidis | |
| ERR037565 | Salmonella enterica subsp. enterica serovar Enteritidis | |
| ERR037566 | Salmonella enterica subsp. enterica serovar Enteritidis | |
| ERR037567 | Salmonella enterica subsp. enterica serovar Enteritidis | |
| ERR037568 | Salmonella enterica subsp. enterica serovar Enteritidis | |
| ERR037569 | Salmonella enterica subsp. enterica serovar Enteritidis | |
| ERR037570 | Salmonella enterica subsp. enterica serovar Enteritidis | |
| ERR037572 | Salmonella enterica subsp. enterica serovar Enteritidis | |
| ERR037573 | Salmonella enterica subsp. enterica serovar Enteritidis | |
| ERR037574 | Salmonella enterica subsp. enterica serovar Enteritidis | |
| ERR037575 | Salmonella enterica subsp. enterica serovar Enteritidis | |
| ERR037576 | Salmonella enterica subsp. enterica serovar Enteritidis | |
| ERR037577 | Salmonella enterica subsp. enterica serovar Enteritidis | |
| ERR037578 | Salmonella enterica subsp. enterica serovar Enteritidis | |
| ERR037579 | Salmonella enterica subsp. enterica serovar Enteritidis | |
| ERR037580 | Salmonella enterica subsp. enterica serovar Enteritidis | |
| ERR037581 | Salmonella enterica subsp. enterica serovar Enteritidis | |
| ERR037582 | Salmonella enterica subsp. enterica serovar Enteritidis | |
| ERR037583 | Salmonella enterica subsp. enterica serovar Enteritidis | |
| ERR037597 | Salmonella enterica subsp. enterica serovar Enteritidis | |
| ERR037598 | Salmonella enterica subsp. enterica serovar Enteritidis | |
| ERR037599 | Salmonella enterica subsp. enterica serovar Enteritidis | |
| ERR037600 | Salmonella enterica subsp. enterica serovar Enteritidis | |
| ERR037601 | Salmonella enterica subsp. enterica serovar Enteritidis | |
| ERR037602 | Salmonella enterica subsp. enterica serovar Enteritidis | |
| ERR037603 | Salmonella enterica subsp. enterica serovar Enteritidis | |
| ERR037604 | Salmonella enterica subsp. enterica serovar Enteritidis | |
| ERR037605 | Salmonella enterica subsp. enterica serovar Enteritidis | |
| ERR037606 | Salmonella enterica subsp. enterica serovar Enteritidis | |
| ERR037607 | Salmonella enterica subsp. enterica serovar Enteritidis | |
| ERR037608 | Salmonella enterica subsp. enterica serovar Enteritidis | |
| ERR037609 | Salmonella enterica subsp. enterica serovar Enteritidis | |
| ERR048502 | Salmonella enterica subsp. enterica serovar Enteritidis | |
| ERR048503 | Salmonella enterica subsp. enterica serovar Enteritidis | |
| ERR048504 | Salmonella enterica subsp. enterica serovar Enteritidis | |
| ERR048505 | Salmonella enterica subsp. enterica serovar Enteritidis | |
| ERR048506 | Salmonella enterica subsp. enterica serovar Enteritidis | |
| ERR048507 | Salmonella enterica subsp. enterica serovar Enteritidis | |
| ERR048508 | Salmonella enterica subsp. enterica serovar Enteritidis | |
| ERR048509 | Salmonella enterica subsp. enterica serovar Enteritidis | |
| ERR048510 | Salmonella enterica subsp. enterica serovar Enteritidis | |
| ERR048511 | Salmonella enterica subsp. enterica serovar Enteritidis | |
| ERR048512 | Salmonella enterica subsp. enterica serovar Enteritidis | |
| ERR048513 | Salmonella enterica subsp. enterica serovar Enteritidis | |
| ERR048514 | Salmonella enterica subsp. enterica serovar Enteritidis | |
| ERR048515 | Salmonella enterica subsp. enterica serovar Enteritidis | |
| ERR048516 | Salmonella enterica subsp. enterica serovar Enteritidis | |
| ERR048517 | Salmonella enterica subsp. enterica serovar Enteritidis | |
| ERR048518 | Salmonella enterica subsp. enterica serovar Enteritidis | |
| ERR048519 | Salmonella enterica subsp. enterica serovar Enteritidis | |
| ERR048520 | Salmonella enterica subsp. enterica serovar Enteritidis | |
| ERR048521 | Salmonella enterica subsp. enterica serovar Enteritidis | |
| ERR048522 | Salmonella enterica subsp. enterica serovar Enteritidis | |
| ERR048523 | Salmonella enterica subsp. enterica serovar Enteritidis | |
| ERR048524 | Salmonella enterica subsp. enterica serovar Enteritidis | |
| ERR048525 | Salmonella enterica subsp. enterica serovar Enteritidis | |
| ERR064830 | Salmonella enterica subsp. enterica serovar Enteritidis | |
| ERR064831 | Salmonella enterica subsp. enterica serovar Enteritidis | |
| ERR064832 | Salmonella enterica subsp. enterica serovar Enteritidis | |
| ERR064833 | Salmonella enterica subsp. enterica serovar Enteritidis | |
| ERR064834 | Salmonella enterica subsp. enterica serovar Enteritidis | |
| ERR064835 | Salmonella enterica subsp. enterica serovar Enteritidis | |
| ERR064843 | Salmonella enterica subsp. enterica serovar Enteritidis | |
| ERR064845 | Salmonella enterica subsp. enterica serovar Enteritidis | |
| ERR1010000 | Salmonella enterica subsp. enterica serovar Enteritidis | |
| ERR1010027 | Salmonella enterica subsp. enterica serovar Enteritidis | |
| ERR1010028 | Salmonella enterica subsp. enterica serovar Enteritidis | |
| ERR1010032 | Salmonella enterica subsp. enterica serovar Enteritidis | |
| ERR1010036 | Salmonella enterica subsp. enterica serovar Enteritidis | |
| ERR1010059 | Salmonella enterica subsp. enterica serovar Enteritidis | |
| ERR1010064 | Salmonella enterica subsp. enterica serovar Enteritidis | |
| ERR1010070 | Salmonella enterica subsp. enterica serovar Enteritidis | |
| ERR1010082 | Salmonella enterica subsp. enterica serovar Enteritidis | |
| ERR1010101 | Salmonella enterica subsp. enterica serovar Enteritidis | |
| ERR1010128 | Salmonella enterica subsp. enterica serovar Enteritidis | |
| ERR1010129 | Salmonella enterica subsp. enterica serovar Enteritidis | |
| ERR1010141 | Salmonella enterica subsp. enterica serovar Enteritidis | |
| ERR1010152 | Salmonella enterica subsp. enterica serovar Enteritidis | |
| ERR1010173 | Salmonella enterica subsp. enterica serovar Enteritidis | |
| ERR1010174 | Salmonella enterica subsp. enterica serovar Enteritidis | |
| ERR1082370 | Salmonella enterica subsp. enterica serovar Enteritidis | |
| ERR1370583 | Salmonella enterica subsp. enterica serovar Enteritidis | |
| ERR1370584 | Salmonella enterica subsp. enterica serovar Enteritidis | |
| ERR1370585 | Salmonella enterica subsp. enterica serovar Enteritidis | |
| ERR1370586 | Salmonella enterica subsp. enterica serovar Enteritidis | |
| ERR1370587 | Salmonella enterica subsp. enterica serovar Enteritidis | |
| ERR1544909 | Salmonella enterica subsp. enterica serovar Enteritidis | |
| ERR1544910 | Salmonella enterica subsp. enterica serovar Enteritidis | |
| ERR1544911 | Salmonella enterica subsp. enterica serovar Enteritidis | |
| ERR1706759 | Salmonella enterica subsp. enterica serovar Enteritidis | |
| ERR1706763 | Salmonella enterica subsp. enterica serovar Enteritidis | |
| ERR1706765 | Salmonella enterica subsp. enterica serovar Enteritidis | |
| ERR1706768 | Salmonella enterica subsp. enterica serovar Enteritidis | |
| ERR1755123 | Salmonella enterica subsp. enterica serovar Enteritidis | |
| ERR1755124 | Salmonella enterica subsp. enterica serovar Enteritidis | |
| ERR1755125 | Salmonella enterica subsp. enterica serovar Enteritidis | |
| ERR1755126 | Salmonella enterica subsp. enterica serovar Enteritidis | |
| ERR1755127 | Salmonella enterica subsp. enterica serovar Enteritidis | |
| ERR1755128 | Salmonella enterica subsp. enterica serovar Enteritidis | |
| ERR1755129 | Salmonella enterica subsp. enterica serovar Enteritidis | |
| ERR1755130 | Salmonella enterica subsp. enterica serovar Enteritidis | |
| ERR1755131 | Salmonella enterica subsp. enterica serovar Enteritidis | |
| ERR1755132 | Salmonella enterica subsp. enterica serovar Enteritidis | |
| ERR1755133 | Salmonella enterica subsp. enterica serovar Enteritidis | |
| ERR1755134 | Salmonella enterica subsp. enterica serovar Enteritidis | |
| ERR1755135 | Salmonella enterica subsp. enterica serovar Enteritidis | |
| ERR1755136 | Salmonella enterica subsp. enterica serovar Enteritidis | |
| ERR1755137 | Salmonella enterica subsp. enterica serovar Enteritidis | |
| ERR1755138 | Salmonella enterica subsp. enterica serovar Enteritidis | |
| ERR1755139 | Salmonella enterica subsp. enterica serovar Enteritidis | |
| ERR1755140 | Salmonella enterica subsp. enterica serovar Enteritidis | |
| ERR1755141 | Salmonella enterica subsp. enterica serovar Enteritidis | |
| ERR1755142 | Salmonella enterica subsp. enterica serovar Enteritidis | |
| ERR1755143 | Salmonella enterica subsp. enterica serovar Enteritidis | |
| ERR1755144 | Salmonella enterica subsp. enterica serovar Enteritidis | |
| ERR1755145 | Salmonella enterica subsp. enterica serovar Enteritidis | |
| ERR1755146 | Salmonella enterica subsp. enterica serovar Enteritidis | |
| ERR1755147 | Salmonella enterica subsp. enterica serovar Enteritidis | |
| ERR1755148 | Salmonella enterica subsp. enterica serovar Enteritidis | |
| ERR1755149 | Salmonella enterica subsp. enterica serovar Enteritidis | |
| ERR1755150 | Salmonella enterica subsp. enterica serovar Enteritidis | |
| ERR1755151 | Salmonella enterica subsp. enterica serovar Enteritidis | |
| ERR1755152 | Salmonella enterica subsp. enterica serovar Enteritidis | |
| ERR1755153 | Salmonella enterica subsp. enterica serovar Enteritidis | |
| ERR1755154 | Salmonella enterica subsp. enterica serovar Enteritidis | |
| ERR1755155 | Salmonella enterica subsp. enterica serovar Enteritidis | |
| ERR1755156 | Salmonella enterica subsp. enterica serovar Enteritidis | |
| ERR1755157 | Salmonella enterica subsp. enterica serovar Enteritidis | |
| ERR1755158 | Salmonella enterica subsp. enterica serovar Enteritidis | |
| ERR1755159 | Salmonella enterica subsp. enterica serovar Enteritidis | |
| ERR1755160 | Salmonella enterica subsp. enterica serovar Enteritidis | |
| ERR1755161 | Salmonella enterica subsp. enterica serovar Enteritidis | |
| ERR1755162 | Salmonella enterica subsp. enterica serovar Enteritidis | |
| ERR1755163 | Salmonella enterica subsp. enterica serovar Enteritidis | |
| ERR1755164 | Salmonella enterica subsp. enterica serovar Enteritidis | |
| ERR1755165 | Salmonella enterica subsp. enterica serovar Enteritidis | |
| ERR1755166 | Salmonella enterica subsp. enterica serovar Enteritidis | |
| ERR1755167 | Salmonella enterica subsp. enterica serovar Enteritidis | |
| ERR1755168 | Salmonella enterica subsp. enterica serovar Enteritidis | |
| ERR1755169 | Salmonella enterica subsp. enterica serovar Enteritidis | |
| ERR1755170 | Salmonella enterica subsp. enterica serovar Enteritidis | |
| ERR1755171 | Salmonella enterica subsp. enterica serovar Enteritidis | |
| ERR1755172 | Salmonella enterica subsp. enterica serovar Enteritidis | |
| ERR1755178 | Salmonella enterica subsp. enterica serovar Enteritidis | |
| ERR1755179 | Salmonella enterica subsp. enterica serovar Enteritidis | |
| ERR1755180 | Salmonella enterica subsp. enterica serovar Enteritidis | |
| ERR1755181 | Salmonella enterica subsp. enterica serovar Enteritidis | |
| ERR1755182 | Salmonella enterica subsp. enterica serovar Enteritidis | |
| ERR1755183 | Salmonella enterica subsp. enterica serovar Enteritidis | |
| ERR1755184 | Salmonella enterica subsp. enterica serovar Enteritidis | |
| ERR1755185 | Salmonella enterica subsp. enterica serovar Enteritidis | |
| ERR1755186 | Salmonella enterica subsp. enterica serovar Enteritidis | |
| ERR1755187 | Salmonella enterica subsp. enterica serovar Enteritidis | |
| ERR1755188 | Salmonella enterica subsp. enterica serovar Enteritidis | |
| ERR1755189 | Salmonella enterica subsp. enterica serovar Enteritidis | |
| ERR1755190 | Salmonella enterica subsp. enterica serovar Enteritidis | |
| ERR1755191 | Salmonella enterica subsp. enterica serovar Enteritidis | |
| ERR1755192 | Salmonella enterica subsp. enterica serovar Enteritidis | |
| ERR1755193 | Salmonella enterica subsp. enterica serovar Enteritidis | |
| ERR1755194 | Salmonella enterica subsp. enterica serovar Enteritidis | |
| ERR1755195 | Salmonella enterica subsp. enterica serovar Enteritidis | |
| ERR1755196 | Salmonella enterica subsp. enterica serovar Enteritidis | |
| ERR1755197 | Salmonella enterica subsp. enterica serovar Enteritidis | |
| ERR1755198 | Salmonella enterica subsp. enterica serovar Enteritidis | |
| ERR1755199 | Salmonella enterica subsp. enterica serovar Enteritidis | |
| ERR1755200 | Salmonella enterica subsp. enterica serovar Enteritidis | |
| ERR1755201 | Salmonella enterica subsp. enterica serovar Enteritidis | |
| ERR1755202 | Salmonella enterica subsp. enterica serovar Enteritidis | |
| ERR1755203 | Salmonella enterica subsp. enterica serovar Enteritidis | |
| ERR1755204 | Salmonella enterica subsp. enterica serovar Enteritidis | |
| ERR1755205 | Salmonella enterica subsp. enterica serovar Enteritidis | |
| ERR1755206 | Salmonella enterica subsp. enterica serovar Enteritidis | |
| ERR1755207 | Salmonella enterica subsp. enterica serovar Enteritidis | |
| ERR1755685 | Salmonella enterica subsp. enterica serovar Enteritidis | |
| ERR1755686 | Salmonella enterica subsp. enterica serovar Enteritidis | |
| ERR1755687 | Salmonella enterica subsp. enterica serovar Enteritidis | |
| ERR1755688 | Salmonella enterica subsp. enterica serovar Enteritidis | |
| ERR1755689 | Salmonella enterica subsp. enterica serovar Enteritidis | |
| ERR1755690 | Salmonella enterica subsp. enterica serovar Enteritidis | |
| ERR1755691 | Salmonella enterica subsp. enterica serovar Enteritidis | |
| ERR1755692 | Salmonella enterica subsp. enterica serovar Enteritidis | |
| ERR1755693 | Salmonella enterica subsp. enterica serovar Enteritidis | |
| ERR1755694 | Salmonella enterica subsp. enterica serovar Enteritidis | |
| ERR1755695 | Salmonella enterica subsp. enterica serovar Enteritidis | |
| ERR1755696 | Salmonella enterica subsp. enterica serovar Enteritidis | |
| ERR1755697 | Salmonella enterica subsp. enterica serovar Enteritidis | |
| ERR1755698 | Salmonella enterica subsp. enterica serovar Enteritidis | |
| ERR1755699 | Salmonella enterica subsp. enterica serovar Enteritidis | |
| ERR1755700 | Salmonella enterica subsp. enterica serovar Enteritidis | |
| ERR1755701 | Salmonella enterica subsp. enterica serovar Enteritidis | |
| ERR1755702 | Salmonella enterica subsp. enterica serovar Enteritidis | |
| ERR1755703 | Salmonella enterica subsp. enterica serovar Enteritidis | |
| ERR1755704 | Salmonella enterica subsp. enterica serovar Enteritidis | |
| ERR1755705 | Salmonella enterica subsp. enterica serovar Enteritidis | |
| ERR1755706 | Salmonella enterica subsp. enterica serovar Enteritidis | |
| ERR1755707 | Salmonella enterica subsp. enterica serovar Enteritidis | |
| ERR1755708 | Salmonella enterica subsp. enterica serovar Enteritidis | |
| ERR1755709 | Salmonella enterica subsp. enterica serovar Enteritidis | |
| ERR1755710 | Salmonella enterica subsp. enterica serovar Enteritidis | |
| ERR1755711 | Salmonella enterica subsp. enterica serovar Enteritidis | |
| ERR1755712 | Salmonella enterica subsp. enterica serovar Enteritidis | |
| ERR1755713 | Salmonella enterica subsp. enterica serovar Enteritidis | |
| ERR1755714 | Salmonella enterica subsp. enterica serovar Enteritidis | |
| ERR1755715 | Salmonella enterica subsp. enterica serovar Enteritidis | |
| ERR1755716 | Salmonella enterica subsp. enterica serovar Enteritidis | |
| ERR1755717 | Salmonella enterica subsp. enterica serovar Enteritidis | |
| ERR1755718 | Salmonella enterica subsp. enterica serovar Enteritidis | |
| ERR1755719 | Salmonella enterica subsp. enterica serovar Enteritidis | |
| ERR1755720 | Salmonella enterica subsp. enterica serovar Enteritidis | |
| ERR1755721 | Salmonella enterica subsp. enterica serovar Enteritidis | |
| ERR1755722 | Salmonella enterica subsp. enterica serovar Enteritidis | |
| ERR1755723 | Salmonella enterica subsp. enterica serovar Enteritidis | |
| ERR1755724 | Salmonella enterica subsp. enterica serovar Enteritidis | |
| ERR1755726 | Salmonella enterica subsp. enterica serovar Enteritidis | |
| ERR1755727 | Salmonella enterica subsp. enterica serovar Enteritidis | |
| ERR1755728 | Salmonella enterica subsp. enterica serovar Enteritidis | |
| ERR1755729 | Salmonella enterica subsp. enterica serovar Enteritidis | |
| ERR1755730 | Salmonella enterica subsp. enterica serovar Enteritidis | |
| ERR1755731 | Salmonella enterica subsp. enterica serovar Enteritidis | |
| ERR1755732 | Salmonella enterica subsp. enterica serovar Enteritidis | |
| ERR1755733 | Salmonella enterica subsp. enterica serovar Enteritidis | |
| ERR1755734 | Salmonella enterica subsp. enterica serovar Enteritidis | |
| ERR1755735 | Salmonella enterica subsp. enterica serovar Enteritidis | |
| ERR1755736 | Salmonella enterica subsp. enterica serovar Enteritidis | |
| ERR1755737 | Salmonella enterica subsp. enterica serovar Enteritidis | |
| ERR1755738 | Salmonella enterica subsp. enterica serovar Enteritidis | |
| ERR1755739 | Salmonella enterica subsp. enterica serovar Enteritidis | |
| ERR1755740 | Salmonella enterica subsp. enterica serovar Enteritidis | |
| ERR1755741 | Salmonella enterica subsp. enterica serovar Enteritidis | |
| ERR1755742 | Salmonella enterica subsp. enterica serovar Enteritidis | |
| ERR1755743 | Salmonella enterica subsp. enterica serovar Enteritidis | |
| ERR1755744 | Salmonella enterica subsp. enterica serovar Enteritidis | |
| ERR1755785 | Salmonella enterica subsp. enterica serovar Enteritidis | |
| ERR1755786 | Salmonella enterica subsp. enterica serovar Enteritidis | |
| ERR1755787 | Salmonella enterica subsp. enterica serovar Enteritidis | |
| ERR1755788 | Salmonella enterica subsp. enterica serovar Enteritidis | |
| ERR1755789 | Salmonella enterica subsp. enterica serovar Enteritidis | |
| ERR1755790 | Salmonella enterica subsp. enterica serovar Enteritidis | |
| ERR1755791 | Salmonella enterica subsp. enterica serovar Enteritidis | |
| ERR1755792 | Salmonella enterica subsp. enterica serovar Enteritidis | |
| ERR1755793 | Salmonella enterica subsp. enterica serovar Enteritidis | |
| ERR1755794 | Salmonella enterica subsp. enterica serovar Enteritidis | |
| ERR1755797 | Salmonella enterica subsp. enterica serovar Enteritidis | |
| ERR1755798 | Salmonella enterica subsp. enterica serovar Enteritidis | |
| ERR1755799 | Salmonella enterica subsp. enterica serovar Enteritidis | |
| ERR1755800 | Salmonella enterica subsp. enterica serovar Enteritidis | |
| ERR1755801 | Salmonella enterica subsp. enterica serovar Enteritidis | |
| ERR1755802 | Salmonella enterica subsp. enterica serovar Enteritidis | |
| ERR1755803 | Salmonella enterica subsp. enterica serovar Enteritidis | |
| ERR1755804 | Salmonella enterica subsp. enterica serovar Enteritidis | |
| ERR1755805 | Salmonella enterica subsp. enterica serovar Enteritidis | |
| ERR1755806 | Salmonella enterica subsp. enterica serovar Enteritidis | |
| ERR1755807 | Salmonella enterica subsp. enterica serovar Enteritidis | |
| ERR1755808 | Salmonella enterica subsp. enterica serovar Enteritidis | |
| ERR1755809 | Salmonella enterica subsp. enterica serovar Enteritidis | |
| ERR1755810 | Salmonella enterica subsp. enterica serovar Enteritidis | |
| ERR1755811 | Salmonella enterica subsp. enterica serovar Enteritidis | |
| ERR1755812 | Salmonella enterica subsp. enterica serovar Enteritidis | |
| ERR1755813 | Salmonella enterica subsp. enterica serovar Enteritidis | |
| ERR1755814 | Salmonella enterica subsp. enterica serovar Enteritidis | |
| ERR1755815 | Salmonella enterica subsp. enterica serovar Enteritidis | |
| ERR1755816 | Salmonella enterica subsp. enterica serovar Enteritidis | |
| ERR1755863 | Salmonella enterica subsp. enterica serovar Enteritidis | |
| ERR1755864 | Salmonella enterica subsp. enterica serovar Enteritidis | |
| ERR1755865 | Salmonella enterica subsp. enterica serovar Enteritidis | |
| ERR1755866 | Salmonella enterica subsp. enterica serovar Enteritidis | |
| ERR1755867 | Salmonella enterica subsp. enterica serovar Enteritidis | |
| ERR1755868 | Salmonella enterica subsp. enterica serovar Enteritidis | |
| ERR1755869 | Salmonella enterica subsp. enterica serovar Enteritidis | |
| ERR1755870 | Salmonella enterica subsp. enterica serovar Enteritidis | |
| ERR1755871 | Salmonella enterica subsp. enterica serovar Enteritidis | |
| ERR1755872 | Salmonella enterica subsp. enterica serovar Enteritidis | |
| ERR1756366 | Salmonella enterica subsp. enterica serovar Enteritidis | |
| ERR1756367 | Salmonella enterica subsp. enterica serovar Enteritidis | |
| ERR1756368 | Salmonella enterica subsp. enterica serovar Enteritidis | |
| ERR1756369 | Salmonella enterica subsp. enterica serovar Enteritidis | |
| ERR1756370 | Salmonella enterica subsp. enterica serovar Enteritidis | |
| ERR1756371 | Salmonella enterica subsp. enterica serovar Enteritidis | |
| ERR1756372 | Salmonella enterica subsp. enterica serovar Enteritidis | |
| ERR1756373 | Salmonella enterica subsp. enterica serovar Enteritidis | |
| ERR1756374 | Salmonella enterica subsp. enterica serovar Enteritidis | |
| ERR1756375 | Salmonella enterica subsp. enterica serovar Enteritidis | |
| ERR1756376 | Salmonella enterica subsp. enterica serovar Enteritidis | |
| ERR1756377 | Salmonella enterica subsp. enterica serovar Enteritidis | |
| ERR1756378 | Salmonella enterica subsp. enterica serovar Enteritidis | |
| ERR1756379 | Salmonella enterica subsp. enterica serovar Enteritidis | |
| ERR1756380 | Salmonella enterica subsp. enterica serovar Enteritidis | |
| ERR1756381 | Salmonella enterica subsp. enterica serovar Enteritidis | |
| ERR1756382 | Salmonella enterica subsp. enterica serovar Enteritidis | |
| ERR1756383 | Salmonella enterica subsp. enterica serovar Enteritidis | |
| ERR1756384 | Salmonella enterica subsp. enterica serovar Enteritidis | |
| ERR1756385 | Salmonella enterica subsp. enterica serovar Enteritidis | |
| ERR1756493 | Salmonella enterica subsp. enterica serovar Enteritidis | |
| ERR1756494 | Salmonella enterica subsp. enterica serovar Enteritidis | |
| ERR1756495 | Salmonella enterica subsp. enterica serovar Enteritidis | |
| ERR1756496 | Salmonella enterica subsp. enterica serovar Enteritidis | |
| ERR1756497 | Salmonella enterica subsp. enterica serovar Enteritidis | |
| ERR1756498 | Salmonella enterica subsp. enterica serovar Enteritidis | |
| ERR1756499 | Salmonella enterica subsp. enterica serovar Enteritidis | |
| ERR1756500 | Salmonella enterica subsp. enterica serovar Enteritidis | |
| ERR1756501 | Salmonella enterica subsp. enterica serovar Enteritidis | |
| ERR1757887 | Salmonella enterica subsp. enterica serovar Enteritidis | |
| ERR1757888 | Salmonella enterica subsp. enterica serovar Enteritidis | |
| ERR1757889 | Salmonella enterica subsp. enterica serovar Enteritidis | |
| ERR1757890 | Salmonella enterica subsp. enterica serovar Enteritidis | |
| ERR1757891 | Salmonella enterica subsp. enterica serovar Enteritidis | |
| ERR1757892 | Salmonella enterica subsp. enterica serovar Enteritidis | |
| ERR1757893 | Salmonella enterica subsp. enterica serovar Enteritidis | |
| ERR1757894 | Salmonella enterica subsp. enterica serovar Enteritidis | |
| ERR1757895 | Salmonella enterica subsp. enterica serovar Enteritidis | |
| ERR1757896 | Salmonella enterica subsp. enterica serovar Enteritidis | |
| ERR1757897 | Salmonella enterica subsp. enterica serovar Enteritidis | |
| ERR1757898 | Salmonella enterica subsp. enterica serovar Enteritidis | |
| ERR1757899 | Salmonella enterica subsp. enterica serovar Enteritidis | |
| ERR1757900 | Salmonella enterica subsp. enterica serovar Enteritidis | |
| ERR1757901 | Salmonella enterica subsp. enterica serovar Enteritidis | |
| ERR1757902 | Salmonella enterica subsp. enterica serovar Enteritidis | |
| ERR1757903 | Salmonella enterica subsp. enterica serovar Enteritidis | |
| ERR1757904 | Salmonella enterica subsp. enterica serovar Enteritidis | |
| ERR1757905 | Salmonella enterica subsp. enterica serovar Enteritidis | |
| ERR1757906 | Salmonella enterica subsp. enterica serovar Enteritidis | |
| ERR1758363 | Salmonella enterica subsp. enterica serovar Enteritidis | |
| ERR1758364 | Salmonella enterica subsp. enterica serovar Enteritidis | |
| ERR1758365 | Salmonella enterica subsp. enterica serovar Enteritidis | |
| ERR1758366 | Salmonella enterica subsp. enterica serovar Enteritidis | |
| ERR1758367 | Salmonella enterica subsp. enterica serovar Enteritidis | |
| ERR1758368 | Salmonella enterica subsp. enterica serovar Enteritidis | |
| ERR1758369 | Salmonella enterica subsp. enterica serovar Enteritidis | |
| ERR1758370 | Salmonella enterica subsp. enterica serovar Enteritidis | |
| ERR1758371 | Salmonella enterica subsp. enterica serovar Enteritidis | |
| ERR1758372 | Salmonella enterica subsp. enterica serovar Enteritidis | |
| ERR1758435 | Salmonella enterica subsp. enterica serovar Enteritidis | |
| ERR1758436 | Salmonella enterica subsp. enterica serovar Enteritidis | |
| ERR1758437 | Salmonella enterica subsp. enterica serovar Enteritidis | |
| ERR1758438 | Salmonella enterica subsp. enterica serovar Enteritidis | |
| ERR1758439 | Salmonella enterica subsp. enterica serovar Enteritidis | |
| ERR1758440 | Salmonella enterica subsp. enterica serovar Enteritidis | |
| ERR1758441 | Salmonella enterica subsp. enterica serovar Enteritidis | |
| ERR1758442 | Salmonella enterica subsp. enterica serovar Enteritidis | |
| ERR1758443 | Salmonella enterica subsp. enterica serovar Enteritidis | |
| ERR1758444 | Salmonella enterica subsp. enterica serovar Enteritidis | |
| ERR1758503 | Salmonella enterica subsp. enterica serovar Enteritidis | |
| ERR1758504 | Salmonella enterica subsp. enterica serovar Enteritidis | |
| ERR1758505 | Salmonella enterica subsp. enterica serovar Enteritidis | |
| ERR1758506 | Salmonella enterica subsp. enterica serovar Enteritidis | |
| ERR1758507 | Salmonella enterica subsp. enterica serovar Enteritidis | |
| ERR1758508 | Salmonella enterica subsp. enterica serovar Enteritidis | |
| ERR1758509 | Salmonella enterica subsp. enterica serovar Enteritidis | |
| ERR1758510 | Salmonella enterica subsp. enterica serovar Enteritidis | |
| ERR1758511 | Salmonella enterica subsp. enterica serovar Enteritidis | |
| ERR1758512 | Salmonella enterica subsp. enterica serovar Enteritidis | |
| ERR1759006 | Salmonella enterica subsp. enterica serovar Enteritidis | |
| ERR1759007 | Salmonella enterica subsp. enterica serovar Enteritidis | |
| ERR1759008 | Salmonella enterica subsp. enterica serovar Enteritidis | |
| ERR1759009 | Salmonella enterica subsp. enterica serovar Enteritidis | |
| ERR1759010 | Salmonella enterica subsp. enterica serovar Enteritidis | |
| ERR1759011 | Salmonella enterica subsp. enterica serovar Enteritidis | |
| ERR1759012 | Salmonella enterica subsp. enterica serovar Enteritidis | |
| ERR1759013 | Salmonella enterica subsp. enterica serovar Enteritidis | |
| ERR1759014 | Salmonella enterica subsp. enterica serovar Enteritidis | |
| ERR1759015 | Salmonella enterica subsp. enterica serovar Enteritidis | |
| ERR1759016 | Salmonella enterica subsp. enterica serovar Enteritidis | |
| ERR1759017 | Salmonella enterica subsp. enterica serovar Enteritidis | |
| ERR1759018 | Salmonella enterica subsp. enterica serovar Enteritidis | |
| ERR1759019 | Salmonella enterica subsp. enterica serovar Enteritidis | |
| ERR1759020 | Salmonella enterica subsp. enterica serovar Enteritidis | |
| ERR1759021 | Salmonella enterica subsp. enterica serovar Enteritidis | |
| ERR1759022 | Salmonella enterica subsp. enterica serovar Enteritidis | |
| ERR1759023 | Salmonella enterica subsp. enterica serovar Enteritidis | |
| ERR1759024 | Salmonella enterica subsp. enterica serovar Enteritidis | |
| ERR1759025 | Salmonella enterica subsp. enterica serovar Enteritidis | |
| ERR1759026 | Salmonella enterica subsp. enterica serovar Enteritidis | |
| ERR1759027 | Salmonella enterica subsp. enterica serovar Enteritidis | |
| ERR1759028 | Salmonella enterica subsp. enterica serovar Enteritidis | |
| ERR1759029 | Salmonella enterica subsp. enterica serovar Enteritidis | |
| ERR1759030 | Salmonella enterica subsp. enterica serovar Enteritidis | |
| ERR1759031 | Salmonella enterica subsp. enterica serovar Enteritidis | |
| ERR1759032 | Salmonella enterica subsp. enterica serovar Enteritidis | |
| ERR1759033 | Salmonella enterica subsp. enterica serovar Enteritidis | |
| ERR1759034 | Salmonella enterica subsp. enterica serovar Enteritidis | |
| ERR1759035 | Salmonella enterica subsp. enterica serovar Enteritidis | |
| ERR1759036 | Salmonella enterica subsp. enterica serovar Enteritidis | |
| ERR1759037 | Salmonella enterica subsp. enterica serovar Enteritidis | |
| ERR1759038 | Salmonella enterica subsp. enterica serovar Enteritidis | |
| ERR1759039 | Salmonella enterica subsp. enterica serovar Enteritidis | |
| ERR1759040 | Salmonella enterica subsp. enterica serovar Enteritidis | |
| ERR1759041 | Salmonella enterica subsp. enterica serovar Enteritidis | |
| ERR1759042 | Salmonella enterica subsp. enterica serovar Enteritidis | |
| ERR1759043 | Salmonella enterica subsp. enterica serovar Enteritidis | |
| ERR1759044 | Salmonella enterica subsp. enterica serovar Enteritidis | |
| ERR1759045 | Salmonella enterica subsp. enterica serovar Enteritidis | |
| ERR1759046 | Salmonella enterica subsp. enterica serovar Enteritidis | |
| ERR1759047 | Salmonella enterica subsp. enterica serovar Enteritidis | |
| ERR1759048 | Salmonella enterica subsp. enterica serovar Enteritidis | |
| ERR1759049 | Salmonella enterica subsp. enterica serovar Enteritidis | |
| ERR1759050 | Salmonella enterica subsp. enterica serovar Enteritidis | |
| ERR1759051 | Salmonella enterica subsp. enterica serovar Enteritidis | |
| ERR1759052 | Salmonella enterica subsp. enterica serovar Enteritidis | |
| ERR1759053 | Salmonella enterica subsp. enterica serovar Enteritidis | |
| ERR1759054 | Salmonella enterica subsp. enterica serovar Enteritidis | |
| ERR1759055 | Salmonella enterica subsp. enterica serovar Enteritidis | |
| ERR1759056 | Salmonella enterica subsp. enterica serovar Enteritidis | |
| ERR1759057 | Salmonella enterica subsp. enterica serovar Enteritidis | |
| ERR1759060 | Salmonella enterica subsp. enterica serovar Enteritidis | |
| ERR1759061 | Salmonella enterica subsp. enterica serovar Enteritidis | |
| ERR1759062 | Salmonella enterica subsp. enterica serovar Enteritidis | |
| ERR1759096 | Salmonella enterica subsp. enterica serovar Enteritidis | |
| ERR1759100 | Salmonella enterica subsp. enterica serovar Enteritidis | |
| ERR1759109 | Salmonella enterica subsp. enterica serovar Enteritidis | |
| ERR1759110 | Salmonella enterica subsp. enterica serovar Enteritidis | |
| ERR1759111 | Salmonella enterica subsp. enterica serovar Enteritidis | |
| ERR1759127 | Salmonella enterica subsp. enterica serovar Enteritidis | |
| ERR1759156 | Salmonella enterica subsp. enterica serovar Enteritidis | |
| ERR1759164 | Salmonella enterica subsp. enterica serovar Enteritidis | |
| ERR1759165 | Salmonella enterica subsp. enterica serovar Enteritidis | |
| ERR1759173 | Salmonella enterica subsp. enterica serovar Enteritidis | |
| ERR1759200 | Salmonella enterica subsp. enterica serovar Enteritidis | |
| ERR1759223 | Salmonella enterica subsp. enterica serovar Enteritidis | |
| ERR1759224 | Salmonella enterica subsp. enterica serovar Enteritidis | |
| ERR1759225 | Salmonella enterica subsp. enterica serovar Enteritidis | |
| ERR1759226 | Salmonella enterica subsp. enterica serovar Enteritidis | |
| ERR1759227 | Salmonella enterica subsp. enterica serovar Enteritidis | |
| ERR1759228 | Salmonella enterica subsp. enterica serovar Enteritidis | |
| ERR1759229 | Salmonella enterica subsp. enterica serovar Enteritidis | |
| ERR1759230 | Salmonella enterica subsp. enterica serovar Enteritidis | |
| ERR1759231 | Salmonella enterica subsp. enterica serovar Enteritidis | |
| ERR1759232 | Salmonella enterica subsp. enterica serovar Enteritidis | |
| ERR1759233 | Salmonella enterica subsp. enterica serovar Enteritidis | |
| ERR1759234 | Salmonella enterica subsp. enterica serovar Enteritidis | |
| ERR1759235 | Salmonella enterica subsp. enterica serovar Enteritidis | |
| ERR1759236 | Salmonella enterica subsp. enterica serovar Enteritidis | |
| ERR1759237 | Salmonella enterica subsp. enterica serovar Enteritidis | |
| ERR1759238 | Salmonella enterica subsp. enterica serovar Enteritidis | |
| ERR1759239 | Salmonella enterica subsp. enterica serovar Enteritidis | |
| ERR1759240 | Salmonella enterica subsp. enterica serovar Enteritidis | |
| ERR1759241 | Salmonella enterica subsp. enterica serovar Enteritidis | |
| ERR1759242 | Salmonella enterica subsp. enterica serovar Enteritidis | |
| ERR1759243 | Salmonella enterica subsp. enterica serovar Enteritidis | |
| ERR1759244 | Salmonella enterica subsp. enterica serovar Enteritidis | |
| ERR1759245 | Salmonella enterica subsp. enterica serovar Enteritidis | |
| ERR1759246 | Salmonella enterica subsp. enterica serovar Enteritidis | |
| ERR1759247 | Salmonella enterica subsp. enterica serovar Enteritidis | |
| ERR1759248 | Salmonella enterica subsp. enterica serovar Enteritidis | |
| ERR1759249 | Salmonella enterica subsp. enterica serovar Enteritidis | |
| ERR1759250 | Salmonella enterica subsp. enterica serovar Enteritidis | |
| ERR1759251 | Salmonella enterica subsp. enterica serovar Enteritidis | |
| ERR1759252 | Salmonella enterica subsp. enterica serovar Enteritidis | |
| ERR1759409 | Salmonella enterica subsp. enterica serovar Enteritidis | |
| ERR1759410 | Salmonella enterica subsp. enterica serovar Enteritidis | |
| ERR1759411 | Salmonella enterica subsp. enterica serovar Enteritidis | |
| ERR1759412 | Salmonella enterica subsp. enterica serovar Enteritidis | |
| ERR1759413 | Salmonella enterica subsp. enterica serovar Enteritidis | |
| ERR1759414 | Salmonella enterica subsp. enterica serovar Enteritidis | |
| ERR1759415 | Salmonella enterica subsp. enterica serovar Enteritidis | |
| ERR1759416 | Salmonella enterica subsp. enterica serovar Enteritidis | |
| ERR1759417 | Salmonella enterica subsp. enterica serovar Enteritidis | |
| ERR1759418 | Salmonella enterica subsp. enterica serovar Enteritidis | |
| ERR1759419 | Salmonella enterica subsp. enterica serovar Enteritidis | |
| ERR1759420 | Salmonella enterica subsp. enterica serovar Enteritidis | |
| ERR1759421 | Salmonella enterica subsp. enterica serovar Enteritidis | |
| ERR1759422 | Salmonella enterica subsp. enterica serovar Enteritidis | |
| ERR1759423 | Salmonella enterica subsp. enterica serovar Enteritidis | |
| ERR1759424 | Salmonella enterica subsp. enterica serovar Enteritidis | |
| ERR1759425 | Salmonella enterica subsp. enterica serovar Enteritidis | |
| ERR1759426 | Salmonella enterica subsp. enterica serovar Enteritidis | |
| ERR1759427 | Salmonella enterica subsp. enterica serovar Enteritidis | |
| ERR1759428 | Salmonella enterica subsp. enterica serovar Enteritidis | |
| ERR1767805 | Salmonella enterica subsp. enterica serovar Enteritidis | |
| ERR1767806 | Salmonella enterica subsp. enterica serovar Enteritidis | |
| ERR1767807 | Salmonella enterica subsp. enterica serovar Enteritidis | |
| ERR1767808 | Salmonella enterica subsp. enterica serovar Enteritidis | |
| ERR1767809 | Salmonella enterica subsp. enterica serovar Enteritidis | |
| ERR1767810 | Salmonella enterica subsp. enterica serovar Enteritidis | |
| ERR1767811 | Salmonella enterica subsp. enterica serovar Enteritidis | |
| ERR1767812 | Salmonella enterica subsp. enterica serovar Enteritidis | |
| ERR1767813 | Salmonella enterica subsp. enterica serovar Enteritidis | |
| ERR1767814 | Salmonella enterica subsp. enterica serovar Enteritidis | |
| ERR1767815 | Salmonella enterica subsp. enterica serovar Enteritidis | |
| ERR1767816 | Salmonella enterica subsp. enterica serovar Enteritidis | |
| ERR1767817 | Salmonella enterica subsp. enterica serovar Enteritidis | |
| ERR1767818 | Salmonella enterica subsp. enterica serovar Enteritidis | |
| ERR1767819 | Salmonella enterica subsp. enterica serovar Enteritidis | |
| ERR1767820 | Salmonella enterica subsp. enterica serovar Enteritidis | |
| ERR1767821 | Salmonella enterica subsp. enterica serovar Enteritidis | |
| ERR1767822 | Salmonella enterica subsp. enterica serovar Enteritidis | |
| ERR1767823 | Salmonella enterica subsp. enterica serovar Enteritidis | |
| ERR1767824 | Salmonella enterica subsp. enterica serovar Enteritidis | |
| ERR1767825 | Salmonella enterica subsp. enterica serovar Enteritidis | |
| ERR1767826 | Salmonella enterica subsp. enterica serovar Enteritidis | |
| ERR1767827 | Salmonella enterica subsp. enterica serovar Enteritidis | |
| ERR1797550 | Salmonella enterica subsp. enterica serovar Enteritidis | |
| ERR1797551 | Salmonella enterica subsp. enterica serovar Enteritidis | |
| ERR1802049 | Salmonella enterica subsp. enterica serovar Enteritidis | |
| ERR1802393 | Salmonella enterica subsp. enterica serovar Enteritidis | |
| ERR1802438 | Salmonella enterica subsp. enterica serovar Enteritidis | |
| ERR1802440 | Salmonella enterica subsp. enterica serovar Enteritidis | |
| ERR1802485 | Salmonella enterica subsp. enterica serovar Enteritidis | |
| ERR1802492 | Salmonella enterica subsp. enterica serovar Enteritidis | |
| ERR1806843 | Salmonella enterica subsp. enterica serovar Enteritidis | |
| ERR1806845 | Salmonella enterica subsp. enterica serovar Enteritidis | |
| ERR1810355 | Salmonella enterica subsp. enterica serovar Enteritidis | |
| ERR1810364 | Salmonella enterica subsp. enterica serovar Enteritidis | |
| ERR1810365 | Salmonella enterica subsp. enterica serovar Enteritidis | |
| ERR1810366 | Salmonella enterica subsp. enterica serovar Enteritidis | |
| ERR1815488 | Salmonella enterica subsp. enterica serovar Enteritidis | |
| ERR1815489 | Salmonella enterica subsp. enterica serovar Enteritidis | |
| ERR1815490 | Salmonella enterica subsp. enterica serovar Enteritidis | |
| ERR1815491 | Salmonella enterica subsp. enterica serovar Enteritidis | |
| ERR1815494 | Salmonella enterica subsp. enterica serovar Enteritidis | |
| ERR1815502 | Salmonella enterica subsp. enterica serovar Enteritidis | |
| ERR1815527 | Salmonella enterica subsp. enterica serovar Enteritidis | |
| ERR1815537 | Salmonella enterica subsp. enterica serovar Enteritidis | |
| ERR1815673 | Salmonella enterica subsp. enterica serovar Enteritidis | |
| ERR1815680 | Salmonella enterica subsp. enterica serovar Enteritidis | |
| ERR1815683 | Salmonella enterica subsp. enterica serovar Enteritidis | |
| ERR1815684 | Salmonella enterica subsp. enterica serovar Enteritidis | |
| ERR1815747 | Salmonella enterica subsp. enterica serovar Enteritidis | |
| ERR1816631 | Salmonella enterica subsp. enterica serovar Enteritidis | |
| ERR1816687 | Salmonella enterica subsp. enterica serovar Enteritidis | |
| ERR1816689 | Salmonella enterica subsp. enterica serovar Enteritidis | |
| ERR1816692 | Salmonella enterica subsp. enterica serovar Enteritidis | |
| ERR1816698 | Salmonella enterica subsp. enterica serovar Enteritidis | |
| ERR1816699 | Salmonella enterica subsp. enterica serovar Enteritidis | |
| ERR1816701 | Salmonella enterica subsp. enterica serovar Enteritidis | |
| ERR1816703 | Salmonella enterica subsp. enterica serovar Enteritidis | |
| ERR1816705 | Salmonella enterica subsp. enterica serovar Enteritidis | |
| ERR1816857 | Salmonella enterica subsp. enterica serovar Enteritidis | |
| ERR1816859 | Salmonella enterica subsp. enterica serovar Enteritidis | |
| ERR1816860 | Salmonella enterica subsp. enterica serovar Enteritidis | |
| ERR1816862 | Salmonella enterica subsp. enterica serovar Enteritidis | |
| ERR1816863 | Salmonella enterica subsp. enterica serovar Enteritidis | |
| ERR1816864 | Salmonella enterica subsp. enterica serovar Enteritidis | |
| ERR1817107 | Salmonella enterica subsp. enterica serovar Enteritidis | |
| ERR1817111 | Salmonella enterica subsp. enterica serovar Enteritidis | |
| ERR1817112 | Salmonella enterica subsp. enterica serovar Enteritidis | |
| ERR1817121 | Salmonella enterica subsp. enterica serovar Enteritidis | |
| ERR1817125 | Salmonella enterica subsp. enterica serovar Enteritidis | |
| ERR1817126 | Salmonella enterica subsp. enterica serovar Enteritidis | |
| ERR1817127 | Salmonella enterica subsp. enterica serovar Enteritidis | |
| ERR1817145 | Salmonella enterica subsp. enterica serovar Enteritidis | |
| ERR1817149 | Salmonella enterica subsp. enterica serovar Enteritidis | |
| ERR1817152 | Salmonella enterica subsp. enterica serovar Enteritidis | |
| ERR1817153 | Salmonella enterica subsp. enterica serovar Enteritidis | |
| ERR1817203 | Salmonella enterica subsp. enterica serovar Enteritidis | |
| ERR1817204 | Salmonella enterica subsp. enterica serovar Enteritidis | |
| ERR1817205 | Salmonella enterica subsp. enterica serovar Enteritidis | |
| ERR1817206 | Salmonella enterica subsp. enterica serovar Enteritidis | |
| ERR1817207 | Salmonella enterica subsp. enterica serovar Enteritidis | |
| ERR1817210 | Salmonella enterica subsp. enterica serovar Enteritidis | |
| ERR1817215 | Salmonella enterica subsp. enterica serovar Enteritidis | |
| ERR1817217 | Salmonella enterica subsp. enterica serovar Enteritidis | |
| ERR1817218 | Salmonella enterica subsp. enterica serovar Enteritidis | |
| ERR1817318 | Salmonella enterica subsp. enterica serovar Enteritidis | |
| ERR1817324 | Salmonella enterica subsp. enterica serovar Enteritidis | |
| ERR1817326 | Salmonella enterica subsp. enterica serovar Enteritidis | |
| ERR1817328 | Salmonella enterica subsp. enterica serovar Enteritidis | |
| ERR1817332 | Salmonella enterica subsp. enterica serovar Enteritidis | |
| ERR1817393 | Salmonella enterica subsp. enterica serovar Enteritidis | |
| ERR1817396 | Salmonella enterica subsp. enterica serovar Enteritidis | |
| ERR1817397 | Salmonella enterica subsp. enterica serovar Enteritidis | |
| ERR1817400 | Salmonella enterica subsp. enterica serovar Enteritidis | |
| ERR1817401 | Salmonella enterica subsp. enterica serovar Enteritidis | |
| ERR1817403 | Salmonella enterica subsp. enterica serovar Enteritidis | |
| ERR1817504 | Salmonella enterica subsp. enterica serovar Enteritidis | |
| ERR1817507 | Salmonella enterica subsp. enterica serovar Enteritidis | |
| ERR1817509 | Salmonella enterica subsp. enterica serovar Enteritidis | |
| ERR1817512 | Salmonella enterica subsp. enterica serovar Enteritidis | |
| ERR1817525 | Salmonella enterica subsp. enterica serovar Enteritidis | |
| ERR1817534 | Salmonella enterica subsp. enterica serovar Enteritidis | |
| ERR1817535 | Salmonella enterica subsp. enterica serovar Enteritidis | |
| ERR1817537 | Salmonella enterica subsp. enterica serovar Enteritidis | |
| ERR1818149 | Salmonella enterica subsp. enterica serovar Enteritidis | |
| ERR1818150 | Salmonella enterica subsp. enterica serovar Enteritidis | |
| ERR1822678 | Salmonella enterica subsp. enterica serovar Enteritidis | |
| ERR1822679 | Salmonella enterica subsp. enterica serovar Enteritidis | |
| ERR1822682 | Salmonella enterica subsp. enterica serovar Enteritidis | |
| ERR1822683 | Salmonella enterica subsp. enterica serovar Enteritidis | |
| ERR1823519 | Salmonella enterica subsp. enterica serovar Enteritidis | |
| ERR1823520 | Salmonella enterica subsp. enterica serovar Enteritidis | |
| ERR1823926 | Salmonella enterica subsp. enterica serovar Enteritidis | |
| ERR1823929 | Salmonella enterica subsp. enterica serovar Enteritidis | |
| ERR1823930 | Salmonella enterica subsp. enterica serovar Enteritidis | |
| ERR1823932 | Salmonella enterica subsp. enterica serovar Enteritidis | |
| ERR1824575 | Salmonella enterica subsp. enterica serovar Enteritidis | |
| ERR1828942 | Salmonella enterica subsp. enterica serovar Enteritidis | |
| ERR1828943 | Salmonella enterica subsp. enterica serovar Enteritidis | |
| ERR1828946 | Salmonella enterica subsp. enterica serovar Enteritidis | |
| ERR1828949 | Salmonella enterica subsp. enterica serovar Enteritidis | |
| ERR1829780 | Salmonella enterica subsp. enterica serovar Enteritidis | |
| ERR1829781 | Salmonella enterica subsp. enterica serovar Enteritidis | |
| ERR1829783 | Salmonella enterica subsp. enterica serovar Enteritidis | |
| ERR1829784 | Salmonella enterica subsp. enterica serovar Enteritidis | |
| ERR1829785 | Salmonella enterica subsp. enterica serovar Enteritidis | |
| ERR1832463 | Salmonella enterica subsp. enterica serovar Enteritidis | |
| ERR1832465 | Salmonella enterica subsp. enterica serovar Enteritidis | |
| ERR1832466 | Salmonella enterica subsp. enterica serovar Enteritidis | |
| ERR1832467 | Salmonella enterica subsp. enterica serovar Enteritidis | |
| ERR1832468 | Salmonella enterica subsp. enterica serovar Enteritidis | |
| ERR1832470 | Salmonella enterica subsp. enterica serovar Enteritidis | |
| ERR1832472 | Salmonella enterica subsp. enterica serovar Enteritidis | |
| ERR1837574 | Salmonella enterica subsp. enterica serovar Enteritidis | |
| ERR1837623 | Salmonella enterica subsp. enterica serovar Enteritidis | |
| ERR1837625 | Salmonella enterica subsp. enterica serovar Enteritidis | |
| ERR1857455 | Salmonella enterica subsp. enterica serovar Enteritidis | |
| ERR1857518 | Salmonella enterica subsp. enterica serovar Enteritidis | |
| ERR1857520 | Salmonella enterica subsp. enterica serovar Enteritidis | |
| ERR1865334 | Salmonella enterica subsp. enterica serovar Enteritidis | |
| ERR1865335 | Salmonella enterica subsp. enterica serovar Enteritidis | |
| ERR1890405 | Salmonella enterica subsp. enterica serovar Enteritidis | |
| ERR1890407 | Salmonella enterica subsp. enterica serovar Enteritidis | |
| ERR1890410 | Salmonella enterica subsp. enterica serovar Enteritidis | |
| ERR1910716 | Salmonella enterica subsp. enterica serovar Enteritidis | |
| ERR1910828 | Salmonella enterica subsp. enterica serovar Enteritidis | |
| ERR1910829 | Salmonella enterica subsp. enterica serovar Enteritidis | |
| ERR1948283 | Salmonella enterica subsp. enterica serovar Enteritidis | |
| ERR1948287 | Salmonella enterica subsp. enterica serovar Enteritidis | |
| ERR1948290 | Salmonella enterica subsp. enterica serovar Enteritidis | |
| ERR1948292 | Salmonella enterica subsp. enterica serovar Enteritidis | |
| ERR1948296 | Salmonella enterica subsp. enterica serovar Enteritidis | |
| ERR197399 | Salmonella enterica subsp. enterica serovar Enteritidis | |
| ERR197400 | Salmonella enterica subsp. enterica serovar Enteritidis | |
| ERR197401 | Salmonella enterica subsp. enterica serovar Enteritidis | |
| ERR197402 | Salmonella enterica subsp. enterica serovar Enteritidis | |
| ERR197403 | Salmonella enterica subsp. enterica serovar Enteritidis | |
| ERR197404 | Salmonella enterica subsp. enterica serovar Enteritidis | |
| ERR197405 | Salmonella enterica subsp. enterica serovar Enteritidis | |
| ERR197406 | Salmonella enterica subsp. enterica serovar Enteritidis | |
| ERR197407 | Salmonella enterica subsp. enterica serovar Enteritidis | |
| ERR197408 | Salmonella enterica subsp. enterica serovar Enteritidis | |
| ERR197409 | Salmonella enterica subsp. enterica serovar Enteritidis | |
| ERR197410 | Salmonella enterica subsp. enterica serovar Enteritidis | |
| ERR197411 | Salmonella enterica subsp. enterica serovar Enteritidis | |
| ERR197412 | Salmonella enterica subsp. enterica serovar Enteritidis | |
| ERR197413 | Salmonella enterica subsp. enterica serovar Enteritidis | |
| ERR197414 | Salmonella enterica subsp. enterica serovar Enteritidis | |
| ERR197415 | Salmonella enterica subsp. enterica serovar Enteritidis | |
| ERR197416 | Salmonella enterica subsp. enterica serovar Enteritidis | |
| ERR197417 | Salmonella enterica subsp. enterica serovar Enteritidis | |
| ERR197418 | Salmonella enterica subsp. enterica serovar Enteritidis | |
| ERR197419 | Salmonella enterica subsp. enterica serovar Enteritidis | |
| ERR197420 | Salmonella enterica subsp. enterica serovar Enteritidis | |
| ERR197421 | Salmonella enterica subsp. enterica serovar Enteritidis | |
| ERR197422 | Salmonella enterica subsp. enterica serovar Enteritidis | |
| ERR197423 | Salmonella enterica subsp. enterica serovar Enteritidis | |
| ERR197424 | Salmonella enterica subsp. enterica serovar Enteritidis | |
| ERR197425 | Salmonella enterica subsp. enterica serovar Enteritidis | |
| ERR197426 | Salmonella enterica subsp. enterica serovar Enteritidis | |
| ERR197427 | Salmonella enterica subsp. enterica serovar Enteritidis | |
| ERR197428 | Salmonella enterica subsp. enterica serovar Enteritidis | |
| ERR197429 | Salmonella enterica subsp. enterica serovar Enteritidis | |
| ERR197430 | Salmonella enterica subsp. enterica serovar Enteritidis | |
| ERR197431 | Salmonella enterica subsp. enterica serovar Enteritidis | |
| ERR197432 | Salmonella enterica subsp. enterica serovar Enteritidis | |
| ERR197433 | Salmonella enterica subsp. enterica serovar Enteritidis | |
| ERR197434 | Salmonella enterica subsp. enterica serovar Enteritidis | |
| ERR197435 | Salmonella enterica subsp. enterica serovar Enteritidis | |
| ERR197436 | Salmonella enterica subsp. enterica serovar Enteritidis | |
| ERR197437 | Salmonella enterica subsp. enterica serovar Enteritidis | |
| ERR197438 | Salmonella enterica subsp. enterica serovar Enteritidis | |
| ERR197439 | Salmonella enterica subsp. enterica serovar Enteritidis | |
| ERR197440 | Salmonella enterica subsp. enterica serovar Enteritidis | |
| ERR197441 | Salmonella enterica subsp. enterica serovar Enteritidis | |
| ERR197442 | Salmonella enterica subsp. enterica serovar Enteritidis | |
| ERR197443 | Salmonella enterica subsp. enterica serovar Enteritidis | |
| ERR197444 | Salmonella enterica subsp. enterica serovar Enteritidis | |
| ERR197445 | Salmonella enterica subsp. enterica serovar Enteritidis | |
| ERR197446 | Salmonella enterica subsp. enterica serovar Enteritidis | |
| ERR2004636 | Salmonella enterica subsp. enterica serovar Enteritidis | |
| ERR2020716 | Salmonella enterica subsp. enterica serovar Enteritidis | |
| ERR2023156 | Salmonella enterica subsp. enterica serovar Enteritidis | |
| ERR2030251 | Salmonella enterica subsp. enterica serovar Enteritidis | |
| ERR2030253 | Salmonella enterica subsp. enterica serovar Enteritidis | |
| ERR2071993 | Salmonella enterica subsp. enterica serovar Enteritidis | |
| ERR2114794 | Salmonella enterica subsp. enterica serovar Enteritidis | |
| ERR2114797 | Salmonella enterica subsp. enterica serovar Enteritidis | |
| ERR2114801 | Salmonella enterica subsp. enterica serovar Enteritidis | |
| ERR2114803 | Salmonella enterica subsp. enterica serovar Enteritidis | |
| ERR2115639 | Salmonella enterica subsp. enterica serovar Enteritidis | |
| ERR2115642 | Salmonella enterica subsp. enterica serovar Enteritidis | |
| ERR2115646 | Salmonella enterica subsp. enterica serovar Enteritidis | |
| ERR2115647 | Salmonella enterica subsp. enterica serovar Enteritidis | |
| ERR2115649 | Salmonella enterica subsp. enterica serovar Enteritidis | |
| ERR2115655 | Salmonella enterica subsp. enterica serovar Enteritidis | |
| ERR2116054 | Salmonella enterica subsp. enterica serovar Enteritidis | |
| ERR2116059 | Salmonella enterica subsp. enterica serovar Enteritidis | |
| ERR2116064 | Salmonella enterica subsp. enterica serovar Enteritidis | |
| ERR2116709 | Salmonella enterica subsp. enterica serovar Enteritidis | |
| ERR2116710 | Salmonella enterica subsp. enterica serovar Enteritidis | |
| ERR2116711 | Salmonella enterica subsp. enterica serovar Enteritidis | |
| ERR2116712 | Salmonella enterica subsp. enterica serovar Enteritidis | |
| ERR212512 | Salmonella enterica subsp. enterica serovar Enteritidis | |
| ERR212513 | Salmonella enterica subsp. enterica serovar Enteritidis | |
| ERR212519 | Salmonella enterica subsp. enterica serovar Enteritidis | |
| ERR212520 | Salmonella enterica subsp. enterica serovar Enteritidis | |
| ERR212524 | Salmonella enterica subsp. enterica serovar Enteritidis | |
| ERR212526 | Salmonella enterica subsp. enterica serovar Enteritidis | |
| ERR212531 | Salmonella enterica subsp. enterica serovar Enteritidis | |
| ERR212539 | Salmonella enterica subsp. enterica serovar Enteritidis | |
| ERR212543 | Salmonella enterica subsp. enterica serovar Enteritidis | |
| ERR212545 | Salmonella enterica subsp. enterica serovar Enteritidis | |
| ERR212550 | Salmonella enterica subsp. enterica serovar Enteritidis | |
| ERR212551 | Salmonella enterica subsp. enterica serovar Enteritidis | |
| ERR212553 | Salmonella enterica subsp. enterica serovar Enteritidis | |
| ERR212554 | Salmonella enterica subsp. enterica serovar Enteritidis | |
| ERR212594 | Salmonella enterica subsp. enterica serovar Enteritidis | |
| ERR212601 | Salmonella enterica subsp. enterica serovar Enteritidis | |
| ERR212602 | Salmonella enterica subsp. enterica serovar Enteritidis | |
| ERR212603 | Salmonella enterica subsp. enterica serovar Enteritidis | |
| ERR212607 | Salmonella enterica subsp. enterica serovar Enteritidis | |
| ERR212614 | Salmonella enterica subsp. enterica serovar Enteritidis | |
| ERR212618 | Salmonella enterica subsp. enterica serovar Enteritidis | |
| ERR212619 | Salmonella enterica subsp. enterica serovar Enteritidis | |
| ERR212620 | Salmonella enterica subsp. enterica serovar Enteritidis | |
| ERR212624 | Salmonella enterica subsp. enterica serovar Enteritidis | |
| ERR212625 | Salmonella enterica subsp. enterica serovar Enteritidis | |
| ERR212626 | Salmonella enterica subsp. enterica serovar Enteritidis | |
| ERR212628 | Salmonella enterica subsp. enterica serovar Enteritidis | |
| ERR212630 | Salmonella enterica subsp. enterica serovar Enteritidis | |
| ERR212633 | Salmonella enterica subsp. enterica serovar Enteritidis | |
| ERR212634 | Salmonella enterica subsp. enterica serovar Enteritidis | |
| ERR212635 | Salmonella enterica subsp. enterica serovar Enteritidis | |
| ERR212644 | Salmonella enterica subsp. enterica serovar Enteritidis | |
| ERR212649 | Salmonella enterica subsp. enterica serovar Enteritidis | |
| ERR212656 | Salmonella enterica subsp. enterica serovar Enteritidis | |
| ERR212658 | Salmonella enterica subsp. enterica serovar Enteritidis | |
| ERR212669 | Salmonella enterica subsp. enterica serovar Enteritidis | |
| ERR212670 | Salmonella enterica subsp. enterica serovar Enteritidis | |
| ERR212671 | Salmonella enterica subsp. enterica serovar Enteritidis | |
| ERR212673 | Salmonella enterica subsp. enterica serovar Enteritidis | |
| ERR212674 | Salmonella enterica subsp. enterica serovar Enteritidis | |
| ERR212676 | Salmonella enterica subsp. enterica serovar Enteritidis | |
| ERR2173655 | Salmonella enterica subsp. enterica serovar Enteritidis | |
| ERR2173659 | Salmonella enterica subsp. enterica serovar Enteritidis | |
| ERR2173662 | Salmonella enterica subsp. enterica serovar Enteritidis | |
| ERR2173664 | Salmonella enterica subsp. enterica serovar Enteritidis | |
| ERR2173666 | Salmonella enterica subsp. enterica serovar Enteritidis | |
| ERR2173670 | Salmonella enterica subsp. enterica serovar Enteritidis | |
| ERR2173671 | Salmonella enterica subsp. enterica serovar Enteritidis | |
| ERR2173674 | Salmonella enterica subsp. enterica serovar Enteritidis | |
| ERR2173681 | Salmonella enterica subsp. enterica serovar Enteritidis | |
| ERR2173683 | Salmonella enterica subsp. enterica serovar Enteritidis | |
| ERR2173687 | Salmonella enterica subsp. enterica serovar Enteritidis | |
| ERR2173691 | Salmonella enterica subsp. enterica serovar Enteritidis | |
| ERR2173698 | Salmonella enterica subsp. enterica serovar Enteritidis | |
| ERR2173700 | Salmonella enterica subsp. enterica serovar Enteritidis | |
| ERR217378 | Salmonella enterica subsp. enterica serovar Enteritidis | |
| ERR217379 | Salmonella enterica subsp. enterica serovar Enteritidis | |
| ERR2173854 | Salmonella enterica subsp. enterica serovar Enteritidis | |
| ERR217386 | Salmonella enterica subsp. enterica serovar Enteritidis | |
| ERR217394 | Salmonella enterica subsp. enterica serovar Enteritidis | |
| ERR217399 | Salmonella enterica subsp. enterica serovar Enteritidis | |
| ERR217402 | Salmonella enterica subsp. enterica serovar Enteritidis | |
| ERR217403 | Salmonella enterica subsp. enterica serovar Enteritidis | |
| ERR217405 | Salmonella enterica subsp. enterica serovar Enteritidis | |
| ERR217421 | Salmonella enterica subsp. enterica serovar Enteritidis | |
| ERR217422 | Salmonella enterica subsp. enterica serovar Enteritidis | |
| ERR217424 | Salmonella enterica subsp. enterica serovar Enteritidis | |
| ERR217426 | Salmonella enterica subsp. enterica serovar Enteritidis | |
| ERR217427 | Salmonella enterica subsp. enterica serovar Enteritidis | |
| ERR217431 | Salmonella enterica subsp. enterica serovar Enteritidis | |
| ERR217434 | Salmonella enterica subsp. enterica serovar Enteritidis | |
| ERR217435 | Salmonella enterica subsp. enterica serovar Enteritidis | |
| ERR217438 | Salmonella enterica subsp. enterica serovar Enteritidis | |
| ERR217439 | Salmonella enterica subsp. enterica serovar Enteritidis | |
| ERR217440 | Salmonella enterica subsp. enterica serovar Enteritidis | |
| ERR217441 | Salmonella enterica subsp. enterica serovar Enteritidis | |
| ERR217442 | Salmonella enterica subsp. enterica serovar Enteritidis | |
| ERR217443 | Salmonella enterica subsp. enterica serovar Enteritidis | |
| ERR217447 | Salmonella enterica subsp. enterica serovar Enteritidis | |
| ERR217448 | Salmonella enterica subsp. enterica serovar Enteritidis | |
| ERR217451 | Salmonella enterica subsp. enterica serovar Enteritidis | |
| ERR217453 | Salmonella enterica subsp. enterica serovar Enteritidis | |
| ERR217457 | Salmonella enterica subsp. enterica serovar Enteritidis | |
| ERR217460 | Salmonella enterica subsp. enterica serovar Enteritidis | |
| ERR217461 | Salmonella enterica subsp. enterica serovar Enteritidis | |
| ERR217462 | Salmonella enterica subsp. enterica serovar Enteritidis | |
| ERR217467 | Salmonella enterica subsp. enterica serovar Enteritidis | |
| ERR217470 | Salmonella enterica subsp. enterica serovar Enteritidis | |
| ERR2193126 | Salmonella enterica subsp. enterica serovar Enteritidis | |
| ERR2193129 | Salmonella enterica subsp. enterica serovar Enteritidis | |
| ERR2193134 | Salmonella enterica subsp. enterica serovar Enteritidis | |
| ERR2193138 | Salmonella enterica subsp. enterica serovar Enteritidis | |
| ERR2193172 | Salmonella enterica subsp. enterica serovar Enteritidis | |
| ERR2193175 | Salmonella enterica subsp. enterica serovar Enteritidis | |
| ERR2193179 | Salmonella enterica subsp. enterica serovar Enteritidis | |
| ERR2193180 | Salmonella enterica subsp. enterica serovar Enteritidis | |
| ERR2193182 | Salmonella enterica subsp. enterica serovar Enteritidis | |
| ERR2193185 | Salmonella enterica subsp. enterica serovar Enteritidis | |
| ERR2193193 | Salmonella enterica subsp. enterica serovar Enteritidis | |
| ERR2200229 | Salmonella enterica subsp. enterica serovar Enteritidis | |
| ERR2200230 | Salmonella enterica subsp. enterica serovar Enteritidis | |
| ERR2200231 | Salmonella enterica subsp. enterica serovar Enteritidis | |
| ERR2200232 | Salmonella enterica subsp. enterica serovar Enteritidis | |
| ERR2200233 | Salmonella enterica subsp. enterica serovar Enteritidis | |
| ERR2200234 | Salmonella enterica subsp. enterica serovar Enteritidis | |
| ERR2200235 | Salmonella enterica subsp. enterica serovar Enteritidis | |
| ERR2200236 | Salmonella enterica subsp. enterica serovar Enteritidis | |
| ERR2200237 | Salmonella enterica subsp. enterica serovar Enteritidis | |
| ERR2200238 | Salmonella enterica subsp. enterica serovar Enteritidis | |
| ERR2200239 | Salmonella enterica subsp. enterica serovar Enteritidis | |
| ERR2200240 | Salmonella enterica subsp. enterica serovar Enteritidis | |
| ERR2200241 | Salmonella enterica subsp. enterica serovar Enteritidis | |
| ERR2200242 | Salmonella enterica subsp. enterica serovar Enteritidis | |
| ERR2200243 | Salmonella enterica subsp. enterica serovar Enteritidis | |
| ERR2200244 | Salmonella enterica subsp. enterica serovar Enteritidis | |
| ERR2204202 | Salmonella enterica subsp. enterica serovar Enteritidis | |
| ERR2204208 | Salmonella enterica subsp. enterica serovar Enteritidis | |
| ERR2204210 | Salmonella enterica subsp. enterica serovar Enteritidis | |
| ERR2204218 | Salmonella enterica subsp. enterica serovar Enteritidis | |
| ERR2204222 | Salmonella enterica subsp. enterica serovar Enteritidis | |
| ERR2215166 | Salmonella enterica subsp. enterica serovar Enteritidis | |
| ERR2215170 | Salmonella enterica subsp. enterica serovar Enteritidis | |
| ERR2215172 | Salmonella enterica subsp. enterica serovar Enteritidis | |
| ERR2226222 | Salmonella enterica subsp. enterica serovar Enteritidis | |
| ERR2226225 | Salmonella enterica subsp. enterica serovar Enteritidis | |
| ERR2226227 | Salmonella enterica subsp. enterica serovar Enteritidis | |
| ERR2226231 | Salmonella enterica subsp. enterica serovar Enteritidis | |
| ERR226504 | Salmonella enterica subsp. enterica serovar Enteritidis | |
| ERR230374 | Salmonella enterica subsp. enterica serovar Enteritidis | |
| ERR230390 | Salmonella enterica subsp. enterica serovar Enteritidis | |
| ERR230394 | Salmonella enterica subsp. enterica serovar Enteritidis | |
| ERR230409 | Salmonella enterica subsp. enterica serovar Enteritidis | |
| ERR233475 | Salmonella enterica subsp. enterica serovar Enteritidis | |
| ERR233476 | Salmonella enterica subsp. enterica serovar Enteritidis | |
| ERR233477 | Salmonella enterica subsp. enterica serovar Enteritidis | |
| ERR233478 | Salmonella enterica subsp. enterica serovar Enteritidis | |
| ERR233479 | Salmonella enterica subsp. enterica serovar Enteritidis | |
| ERR233480 | Salmonella enterica subsp. enterica serovar Enteritidis | |
| ERR233481 | Salmonella enterica subsp. enterica serovar Enteritidis | |
| ERR233482 | Salmonella enterica subsp. enterica serovar Enteritidis | |
| ERR233483 | Salmonella enterica subsp. enterica serovar Enteritidis | |
| ERR233484 | Salmonella enterica subsp. enterica serovar Enteritidis | |
| ERR233485 | Salmonella enterica subsp. enterica serovar Enteritidis | |
| ERR233486 | Salmonella enterica subsp. enterica serovar Enteritidis | |
| ERR233487 | Salmonella enterica subsp. enterica serovar Enteritidis | |
| ERR233488 | Salmonella enterica subsp. enterica serovar Enteritidis | |
| ERR233489 | Salmonella enterica subsp. enterica serovar Enteritidis | |
| ERR233490 | Salmonella enterica subsp. enterica serovar Enteritidis | |
| ERR233491 | Salmonella enterica subsp. enterica serovar Enteritidis | |
| ERR233492 | Salmonella enterica subsp. enterica serovar Enteritidis | |
| ERR233511 | Salmonella enterica subsp. enterica serovar Enteritidis | |
| ERR233512 | Salmonella enterica subsp. enterica serovar Enteritidis | |
| ERR233513 | Salmonella enterica subsp. enterica serovar Enteritidis | |
| ERR233514 | Salmonella enterica subsp. enterica serovar Enteritidis | |
| ERR233515 | Salmonella enterica subsp. enterica serovar Enteritidis | |
| ERR233516 | Salmonella enterica subsp. enterica serovar Enteritidis | |
| ERR233517 | Salmonella enterica subsp. enterica serovar Enteritidis | |
| ERR233518 | Salmonella enterica subsp. enterica serovar Enteritidis | |
| ERR233519 | Salmonella enterica subsp. enterica serovar Enteritidis | |
| ERR233520 | Salmonella enterica subsp. enterica serovar Enteritidis | |
| ERR233521 | Salmonella enterica subsp. enterica serovar Enteritidis | |
| ERR233522 | Salmonella enterica subsp. enterica serovar Enteritidis | |
| ERR233523 | Salmonella enterica subsp. enterica serovar Enteritidis | |
| ERR233524 | Salmonella enterica subsp. enterica serovar Enteritidis | |
| ERR233525 | Salmonella enterica subsp. enterica serovar Enteritidis | |
| ERR233526 | Salmonella enterica subsp. enterica serovar Enteritidis | |
| ERR233527 | Salmonella enterica subsp. enterica serovar Enteritidis | |
| ERR233528 | Salmonella enterica subsp. enterica serovar Enteritidis | |
| ERR233547 | Salmonella enterica subsp. enterica serovar Enteritidis | |
| ERR233548 | Salmonella enterica subsp. enterica serovar Enteritidis | |
| ERR233549 | Salmonella enterica subsp. enterica serovar Enteritidis | |
| ERR233550 | Salmonella enterica subsp. enterica serovar Enteritidis | |
| ERR233551 | Salmonella enterica subsp. enterica serovar Enteritidis | |
| ERR233552 | Salmonella enterica subsp. enterica serovar Enteritidis | |
| ERR233553 | Salmonella enterica subsp. enterica serovar Enteritidis | |
| ERR233554 | Salmonella enterica subsp. enterica serovar Enteritidis | |
| ERR233555 | Salmonella enterica subsp. enterica serovar Enteritidis | |
| ERR233556 | Salmonella enterica subsp. enterica serovar Enteritidis | |
| ERR233557 | Salmonella enterica subsp. enterica serovar Enteritidis | |
| ERR233558 | Salmonella enterica subsp. enterica serovar Enteritidis | |
| ERR233559 | Salmonella enterica subsp. enterica serovar Enteritidis | |
| ERR233560 | Salmonella enterica subsp. enterica serovar Enteritidis | |
| ERR233561 | Salmonella enterica subsp. enterica serovar Enteritidis | |
| ERR233562 | Salmonella enterica subsp. enterica serovar Enteritidis | |
| ERR233563 | Salmonella enterica subsp. enterica serovar Enteritidis | |
| ERR233564 | Salmonella enterica subsp. enterica serovar Enteritidis | |
| ERR233583 | Salmonella enterica subsp. enterica serovar Enteritidis | |
| ERR233584 | Salmonella enterica subsp. enterica serovar Enteritidis | |
| ERR233585 | Salmonella enterica subsp. enterica serovar Enteritidis | |
| ERR233586 | Salmonella enterica subsp. enterica serovar Enteritidis | |
| ERR233587 | Salmonella enterica subsp. enterica serovar Enteritidis | |
| ERR233588 | Salmonella enterica subsp. enterica serovar Enteritidis | |
| ERR233589 | Salmonella enterica subsp. enterica serovar Enteritidis | |
| ERR233590 | Salmonella enterica subsp. enterica serovar Enteritidis | |
| ERR233591 | Salmonella enterica subsp. enterica serovar Enteritidis | |
| ERR233592 | Salmonella enterica subsp. enterica serovar Enteritidis | |
| ERR233593 | Salmonella enterica subsp. enterica serovar Enteritidis | |
| ERR233594 | Salmonella enterica subsp. enterica serovar Enteritidis | |
| ERR233595 | Salmonella enterica subsp. enterica serovar Enteritidis | |
| ERR233596 | Salmonella enterica subsp. enterica serovar Enteritidis | |
| ERR233597 | Salmonella enterica subsp. enterica serovar Enteritidis | |
| ERR233598 | Salmonella enterica subsp. enterica serovar Enteritidis | |
| ERR233599 | Salmonella enterica subsp. enterica serovar Enteritidis | |
| ERR233600 | Salmonella enterica subsp. enterica serovar Enteritidis | |
| ERR233619 | Salmonella enterica subsp. enterica serovar Enteritidis | |
| ERR233620 | Salmonella enterica subsp. enterica serovar Enteritidis | |
| ERR233621 | Salmonella enterica subsp. enterica serovar Enteritidis | |
| ERR233622 | Salmonella enterica subsp. enterica serovar Enteritidis | |
| ERR233623 | Salmonella enterica subsp. enterica serovar Enteritidis | |
| ERR233624 | Salmonella enterica subsp. enterica serovar Enteritidis | |
| ERR233625 | Salmonella enterica subsp. enterica serovar Enteritidis | |
| ERR233626 | Salmonella enterica subsp. enterica serovar Enteritidis | |
| ERR233627 | Salmonella enterica subsp. enterica serovar Enteritidis | |
| ERR233628 | Salmonella enterica subsp. enterica serovar Enteritidis | |
| ERR233629 | Salmonella enterica subsp. enterica serovar Enteritidis | |
| ERR233630 | Salmonella enterica subsp. enterica serovar Enteritidis | |
| ERR233631 | Salmonella enterica subsp. enterica serovar Enteritidis | |
| ERR233632 | Salmonella enterica subsp. enterica serovar Enteritidis | |
| ERR233633 | Salmonella enterica subsp. enterica serovar Enteritidis | |
| ERR233634 | Salmonella enterica subsp. enterica serovar Enteritidis | |
| ERR233635 | Salmonella enterica subsp. enterica serovar Enteritidis | |
| ERR233636 | Salmonella enterica subsp. enterica serovar Enteritidis | |
| ERR233655 | Salmonella enterica subsp. enterica serovar Enteritidis | |
| ERR233656 | Salmonella enterica subsp. enterica serovar Enteritidis | |
| ERR233657 | Salmonella enterica subsp. enterica serovar Enteritidis | |
| ERR233658 | Salmonella enterica subsp. enterica serovar Enteritidis | |
| ERR233659 | Salmonella enterica subsp. enterica serovar Enteritidis | |
| ERR233660 | Salmonella enterica subsp. enterica serovar Enteritidis | |
| ERR233661 | Salmonella enterica subsp. enterica serovar Enteritidis | |
| ERR233662 | Salmonella enterica subsp. enterica serovar Enteritidis | |
| ERR233663 | Salmonella enterica subsp. enterica serovar Enteritidis | |
| ERR233664 | Salmonella enterica subsp. enterica serovar Enteritidis | |
| ERR233665 | Salmonella enterica subsp. enterica serovar Enteritidis | |
| ERR233666 | Salmonella enterica subsp. enterica serovar Enteritidis | |
| ERR233667 | Salmonella enterica subsp. enterica serovar Enteritidis | |
| ERR233668 | Salmonella enterica subsp. enterica serovar Enteritidis | |
| ERR233669 | Salmonella enterica subsp. enterica serovar Enteritidis | |
| ERR233670 | Salmonella enterica subsp. enterica serovar Enteritidis | |
| ERR233671 | Salmonella enterica subsp. enterica serovar Enteritidis | |
| ERR233672 | Salmonella enterica subsp. enterica serovar Enteritidis | |
| ERR233691 | Salmonella enterica subsp. enterica serovar Enteritidis | |
| ERR233692 | Salmonella enterica subsp. enterica serovar Enteritidis | |
| ERR233693 | Salmonella enterica subsp. enterica serovar Enteritidis | |
| ERR233694 | Salmonella enterica subsp. enterica serovar Enteritidis | |
| ERR233695 | Salmonella enterica subsp. enterica serovar Enteritidis | |
| ERR233696 | Salmonella enterica subsp. enterica serovar Enteritidis | |
| ERR233697 | Salmonella enterica subsp. enterica serovar Enteritidis | |
| ERR233698 | Salmonella enterica subsp. enterica serovar Enteritidis | |
| ERR233699 | Salmonella enterica subsp. enterica serovar Enteritidis | |
| ERR233700 | Salmonella enterica subsp. enterica serovar Enteritidis | |
| ERR233701 | Salmonella enterica subsp. enterica serovar Enteritidis | |
| ERR233702 | Salmonella enterica subsp. enterica serovar Enteritidis | |
| ERR233703 | Salmonella enterica subsp. enterica serovar Enteritidis | |
| ERR233704 | Salmonella enterica subsp. enterica serovar Enteritidis | |
| ERR233705 | Salmonella enterica subsp. enterica serovar Enteritidis | |
| ERR233706 | Salmonella enterica subsp. enterica serovar Enteritidis | |
| ERR233707 | Salmonella enterica subsp. enterica serovar Enteritidis | |
| ERR233708 | Salmonella enterica subsp. enterica serovar Enteritidis | |
| ERR233727 | Salmonella enterica subsp. enterica serovar Enteritidis | |
| ERR233728 | Salmonella enterica subsp. enterica serovar Enteritidis | |
| ERR233729 | Salmonella enterica subsp. enterica serovar Enteritidis | |
| ERR233730 | Salmonella enterica subsp. enterica serovar Enteritidis | |
| ERR233731 | Salmonella enterica subsp. enterica serovar Enteritidis | |
| ERR233732 | Salmonella enterica subsp. enterica serovar Enteritidis | |
| ERR233733 | Salmonella enterica subsp. enterica serovar Enteritidis | |
| ERR233734 | Salmonella enterica subsp. enterica serovar Enteritidis | |
| ERR233735 | Salmonella enterica subsp. enterica serovar Enteritidis | |
| ERR233736 | Salmonella enterica subsp. enterica serovar Enteritidis | |
| ERR233737 | Salmonella enterica subsp. enterica serovar Enteritidis | |
| ERR233738 | Salmonella enterica subsp. enterica serovar Enteritidis | |
| ERR233739 | Salmonella enterica subsp. enterica serovar Enteritidis | |
| ERR233740 | Salmonella enterica subsp. enterica serovar Enteritidis | |
| ERR233741 | Salmonella enterica subsp. enterica serovar Enteritidis | |
| ERR233742 | Salmonella enterica subsp. enterica serovar Enteritidis | |
| ERR233743 | Salmonella enterica subsp. enterica serovar Enteritidis | |
| ERR233744 | Salmonella enterica subsp. enterica serovar Enteritidis | |
| ERR233763 | Salmonella enterica subsp. enterica serovar Enteritidis | |
| ERR233764 | Salmonella enterica subsp. enterica serovar Enteritidis | |
| ERR233765 | Salmonella enterica subsp. enterica serovar Enteritidis | |
| ERR233766 | Salmonella enterica subsp. enterica serovar Enteritidis | |
| ERR233767 | Salmonella enterica subsp. enterica serovar Enteritidis | |
| ERR233768 | Salmonella enterica subsp. enterica serovar Enteritidis | |
| ERR233769 | Salmonella enterica subsp. enterica serovar Enteritidis | |
| ERR233770 | Salmonella enterica subsp. enterica serovar Enteritidis | |
| ERR233771 | Salmonella enterica subsp. enterica serovar Enteritidis | |
| ERR233772 | Salmonella enterica subsp. enterica serovar Enteritidis | |
| ERR233773 | Salmonella enterica subsp. enterica serovar Enteritidis | |
| ERR233774 | Salmonella enterica subsp. enterica serovar Enteritidis | |
| ERR233775 | Salmonella enterica subsp. enterica serovar Enteritidis | |
| ERR233776 | Salmonella enterica subsp. enterica serovar Enteritidis | |
| ERR233777 | Salmonella enterica subsp. enterica serovar Enteritidis | |
| ERR233778 | Salmonella enterica subsp. enterica serovar Enteritidis | |
| ERR233779 | Salmonella enterica subsp. enterica serovar Enteritidis | |
| ERR233780 | Salmonella enterica subsp. enterica serovar Enteritidis | |
| ERR233799 | Salmonella enterica subsp. enterica serovar Enteritidis | |
| ERR233800 | Salmonella enterica subsp. enterica serovar Enteritidis | |
| ERR233801 | Salmonella enterica subsp. enterica serovar Enteritidis | |
| ERR233802 | Salmonella enterica subsp. enterica serovar Enteritidis | |
| ERR233803 | Salmonella enterica subsp. enterica serovar Enteritidis | |
| ERR233804 | Salmonella enterica subsp. enterica serovar Enteritidis | |
| ERR233805 | Salmonella enterica subsp. enterica serovar Enteritidis | |
| ERR233806 | Salmonella enterica subsp. enterica serovar Enteritidis | |
| ERR233807 | Salmonella enterica subsp. enterica serovar Enteritidis | |
| ERR233808 | Salmonella enterica subsp. enterica serovar Enteritidis | |
| ERR233809 | Salmonella enterica subsp. enterica serovar Enteritidis | |
| ERR233810 | Salmonella enterica subsp. enterica serovar Enteritidis | |
| ERR233811 | Salmonella enterica subsp. enterica serovar Enteritidis | |
| ERR233812 | Salmonella enterica subsp. enterica serovar Enteritidis | |
| ERR233813 | Salmonella enterica subsp. enterica serovar Enteritidis | |
| ERR233814 | Salmonella enterica subsp. enterica serovar Enteritidis | |
| ERR233815 | Salmonella enterica subsp. enterica serovar Enteritidis | |
| ERR233816 | Salmonella enterica subsp. enterica serovar Enteritidis | |
| ERR233835 | Salmonella enterica subsp. enterica serovar Enteritidis | |
| ERR233836 | Salmonella enterica subsp. enterica serovar Enteritidis | |
| ERR233837 | Salmonella enterica subsp. enterica serovar Enteritidis | |
| ERR233838 | Salmonella enterica subsp. enterica serovar Enteritidis | |
| ERR233839 | Salmonella enterica subsp. enterica serovar Enteritidis | |
| ERR233840 | Salmonella enterica subsp. enterica serovar Enteritidis | |
| ERR233841 | Salmonella enterica subsp. enterica serovar Enteritidis | |
| ERR233842 | Salmonella enterica subsp. enterica serovar Enteritidis | |
| ERR233843 | Salmonella enterica subsp. enterica serovar Enteritidis | |
| ERR233844 | Salmonella enterica subsp. enterica serovar Enteritidis | |
| ERR233845 | Salmonella enterica subsp. enterica serovar Enteritidis | |
| ERR233846 | Salmonella enterica subsp. enterica serovar Enteritidis | |
| ERR233847 | Salmonella enterica subsp. enterica serovar Enteritidis | |
| ERR233848 | Salmonella enterica subsp. enterica serovar Enteritidis | |
| ERR233849 | Salmonella enterica subsp. enterica serovar Enteritidis | |
| ERR233850 | Salmonella enterica subsp. enterica serovar Enteritidis | |
| ERR233851 | Salmonella enterica subsp. enterica serovar Enteritidis | |
| ERR233852 | Salmonella enterica subsp. enterica serovar Enteritidis | |
| ERR233871 | Salmonella enterica subsp. enterica serovar Enteritidis | |
| ERR233872 | Salmonella enterica subsp. enterica serovar Enteritidis | |
| ERR233873 | Salmonella enterica subsp. enterica serovar Enteritidis | |
| ERR233874 | Salmonella enterica subsp. enterica serovar Enteritidis | |
| ERR233875 | Salmonella enterica subsp. enterica serovar Enteritidis | |
| ERR233876 | Salmonella enterica subsp. enterica serovar Enteritidis | |
| ERR233877 | Salmonella enterica subsp. enterica serovar Enteritidis | |
| ERR233878 | Salmonella enterica subsp. enterica serovar Enteritidis | |
| ERR233879 | Salmonella enterica subsp. enterica serovar Enteritidis | |
| ERR233880 | Salmonella enterica subsp. enterica serovar Enteritidis | |
| ERR233881 | Salmonella enterica subsp. enterica serovar Enteritidis | |
| ERR233882 | Salmonella enterica subsp. enterica serovar Enteritidis | |
| ERR233883 | Salmonella enterica subsp. enterica serovar Enteritidis | |
| ERR233884 | Salmonella enterica subsp. enterica serovar Enteritidis | |
| ERR233885 | Salmonella enterica subsp. enterica serovar Enteritidis | |
| ERR233886 | Salmonella enterica subsp. enterica serovar Enteritidis | |
| ERR233887 | Salmonella enterica subsp. enterica serovar Enteritidis | |
| ERR233888 | Salmonella enterica subsp. enterica serovar Enteritidis | |
| ERR233907 | Salmonella enterica subsp. enterica serovar Enteritidis | |
| ERR233908 | Salmonella enterica subsp. enterica serovar Enteritidis | |
| ERR233909 | Salmonella enterica subsp. enterica serovar Enteritidis | |
| ERR233910 | Salmonella enterica subsp. enterica serovar Enteritidis | |
| ERR233911 | Salmonella enterica subsp. enterica serovar Enteritidis | |
| ERR233912 | Salmonella enterica subsp. enterica serovar Enteritidis | |
| ERR233913 | Salmonella enterica subsp. enterica serovar Enteritidis | |
| ERR233914 | Salmonella enterica subsp. enterica serovar Enteritidis | |
| ERR233915 | Salmonella enterica subsp. enterica serovar Enteritidis | |
| ERR233916 | Salmonella enterica subsp. enterica serovar Enteritidis | |
| ERR233917 | Salmonella enterica subsp. enterica serovar Enteritidis | |
| ERR233918 | Salmonella enterica subsp. enterica serovar Enteritidis | |
| ERR233919 | Salmonella enterica subsp. enterica serovar Enteritidis | |
| ERR233920 | Salmonella enterica subsp. enterica serovar Enteritidis | |
| ERR233921 | Salmonella enterica subsp. enterica serovar Enteritidis | |
| ERR233922 | Salmonella enterica subsp. enterica serovar Enteritidis | |
| ERR233923 | Salmonella enterica subsp. enterica serovar Enteritidis | |
| ERR233924 | Salmonella enterica subsp. enterica serovar Enteritidis | |
| ERR233943 | Salmonella enterica subsp. enterica serovar Enteritidis | |
| ERR233944 | Salmonella enterica subsp. enterica serovar Enteritidis | |
| ERR233945 | Salmonella enterica subsp. enterica serovar Enteritidis | |
| ERR233946 | Salmonella enterica subsp. enterica serovar Enteritidis | |
| ERR233947 | Salmonella enterica subsp. enterica serovar Enteritidis | |
| ERR233948 | Salmonella enterica subsp. enterica serovar Enteritidis | |
| ERR233949 | Salmonella enterica subsp. enterica serovar Enteritidis | |
| ERR233950 | Salmonella enterica subsp. enterica serovar Enteritidis | |
| ERR233951 | Salmonella enterica subsp. enterica serovar Enteritidis | |
| ERR233952 | Salmonella enterica subsp. enterica serovar Enteritidis | |
| ERR233953 | Salmonella enterica subsp. enterica serovar Enteritidis | |
| ERR233954 | Salmonella enterica subsp. enterica serovar Enteritidis | |
| ERR233955 | Salmonella enterica subsp. enterica serovar Enteritidis | |
| ERR233956 | Salmonella enterica subsp. enterica serovar Enteritidis | |
| ERR233957 | Salmonella enterica subsp. enterica serovar Enteritidis | |
| ERR233958 | Salmonella enterica subsp. enterica serovar Enteritidis | |
| ERR233959 | Salmonella enterica subsp. enterica serovar Enteritidis | |
| ERR233960 | Salmonella enterica subsp. enterica serovar Enteritidis | |
| ERR233979 | Salmonella enterica subsp. enterica serovar Enteritidis | |
| ERR233980 | Salmonella enterica subsp. enterica serovar Enteritidis | |
| ERR233981 | Salmonella enterica subsp. enterica serovar Enteritidis | |
| ERR233982 | Salmonella enterica subsp. enterica serovar Enteritidis | |
| ERR233983 | Salmonella enterica subsp. enterica serovar Enteritidis | |
| ERR233984 | Salmonella enterica subsp. enterica serovar Enteritidis | |
| ERR233985 | Salmonella enterica subsp. enterica serovar Enteritidis | |
| ERR233986 | Salmonella enterica subsp. enterica serovar Enteritidis | |
| ERR233987 | Salmonella enterica subsp. enterica serovar Enteritidis | |
| ERR233988 | Salmonella enterica subsp. enterica serovar Enteritidis | |
| ERR233989 | Salmonella enterica subsp. enterica serovar Enteritidis | |
| ERR233990 | Salmonella enterica subsp. enterica serovar Enteritidis | |
| ERR233991 | Salmonella enterica subsp. enterica serovar Enteritidis | |
| ERR233992 | Salmonella enterica subsp. enterica serovar Enteritidis | |
| ERR233993 | Salmonella enterica subsp. enterica serovar Enteritidis | |
| ERR233994 | Salmonella enterica subsp. enterica serovar Enteritidis | |
| ERR233995 | Salmonella enterica subsp. enterica serovar Enteritidis | |
| ERR233996 | Salmonella enterica subsp. enterica serovar Enteritidis | |
| ERR234015 | Salmonella enterica subsp. enterica serovar Enteritidis | |
| ERR234016 | Salmonella enterica subsp. enterica serovar Enteritidis | |
| ERR234017 | Salmonella enterica subsp. enterica serovar Enteritidis | |
| ERR234018 | Salmonella enterica subsp. enterica serovar Enteritidis | |
| ERR234019 | Salmonella enterica subsp. enterica serovar Enteritidis | |
| ERR234020 | Salmonella enterica subsp. enterica serovar Enteritidis | |
| ERR234021 | Salmonella enterica subsp. enterica serovar Enteritidis | |
| ERR234022 | Salmonella enterica subsp. enterica serovar Enteritidis | |
| ERR234023 | Salmonella enterica subsp. enterica serovar Enteritidis | |
| ERR234024 | Salmonella enterica subsp. enterica serovar Enteritidis | |
| ERR234025 | Salmonella enterica subsp. enterica serovar Enteritidis | |
| ERR234026 | Salmonella enterica subsp. enterica serovar Enteritidis | |
| ERR234027 | Salmonella enterica subsp. enterica serovar Enteritidis | |
| ERR234028 | Salmonella enterica subsp. enterica serovar Enteritidis | |
| ERR234029 | Salmonella enterica subsp. enterica serovar Enteritidis | |
| ERR234030 | Salmonella enterica subsp. enterica serovar Enteritidis | |
| ERR234031 | Salmonella enterica subsp. enterica serovar Enteritidis | |
| ERR234032 | Salmonella enterica subsp. enterica serovar Enteritidis | |
| ERR234051 | Salmonella enterica subsp. enterica serovar Enteritidis | |
| ERR234052 | Salmonella enterica subsp. enterica serovar Enteritidis | |
| ERR234055 | Salmonella enterica subsp. enterica serovar Enteritidis | |
| ERR234056 | Salmonella enterica subsp. enterica serovar Enteritidis | |
| ERR234061 | Salmonella enterica subsp. enterica serovar Enteritidis | |
| ERR234062 | Salmonella enterica subsp. enterica serovar Enteritidis | |
| ERR234065 | Salmonella enterica subsp. enterica serovar Enteritidis | |
| ERR234066 | Salmonella enterica subsp. enterica serovar Enteritidis | |
| ERR234067 | Salmonella enterica subsp. enterica serovar Enteritidis | |
| ERR234068 | Salmonella enterica subsp. enterica serovar Enteritidis | |
| ERR234071 | Salmonella enterica subsp. enterica serovar Enteritidis | |
| ERR234072 | Salmonella enterica subsp. enterica serovar Enteritidis | |
| ERR234077 | Salmonella enterica subsp. enterica serovar Enteritidis | |
| ERR234078 | Salmonella enterica subsp. enterica serovar Enteritidis | |
| ERR234081 | Salmonella enterica subsp. enterica serovar Enteritidis | |
| ERR234082 | Salmonella enterica subsp. enterica serovar Enteritidis | |
| ERR235102 | Salmonella enterica subsp. enterica serovar Enteritidis | |
| ERR235103 | Salmonella enterica subsp. enterica serovar Enteritidis str. P125109 | |
| ERR235104 | Salmonella enterica subsp. enterica serovar Enteritidis str. P125109 | |
| ERR235105 | Salmonella enterica subsp. enterica serovar Enteritidis | |
| ERR235106 | Salmonella enterica subsp. enterica serovar Enteritidis | |
| ERR235107 | Salmonella enterica subsp. enterica serovar Enteritidis | |
| ERR235108 | Salmonella enterica subsp. enterica serovar Enteritidis | |
| ERR235109 | Salmonella enterica subsp. enterica serovar Enteritidis | |
| ERR235110 | Salmonella enterica subsp. enterica serovar Enteritidis | |
| ERR235111 | Salmonella enterica subsp. enterica serovar Enteritidis | |
| ERR235112 | Salmonella enterica subsp. enterica serovar Enteritidis | |
| ERR235113 | Salmonella enterica subsp. enterica serovar Enteritidis | |
| ERR235114 | Salmonella enterica subsp. enterica serovar Enteritidis | |
| ERR235115 | Salmonella enterica subsp. enterica serovar Enteritidis | |
| ERR235116 | Salmonella enterica subsp. enterica serovar Enteritidis str. P125109 | |
| ERR235117 | Salmonella enterica subsp. enterica serovar Enteritidis str. P125109 | |
| ERR235118 | Salmonella enterica subsp. enterica serovar Enteritidis | |
| ERR235119 | Salmonella enterica subsp. enterica serovar Enteritidis | |
| ERR235120 | Salmonella enterica subsp. enterica serovar Enteritidis | |
| ERR235121 | Salmonella enterica subsp. enterica serovar Enteritidis str. P125109 | |
| ERR235122 | Salmonella enterica subsp. enterica serovar Enteritidis str. P125109 | |
| ERR235123 | Salmonella enterica subsp. enterica serovar Enteritidis | |
| ERR235124 | Salmonella enterica subsp. enterica serovar Enteritidis str. P125109 | |
| ERR235125 | Salmonella enterica subsp. enterica serovar Enteritidis | |
| ERR235126 | Salmonella enterica subsp. enterica serovar Enteritidis | |
| ERR235127 | Salmonella enterica subsp. enterica serovar Enteritidis | |
| ERR235128 | Salmonella enterica subsp. enterica serovar Enteritidis | |
| ERR235129 | Salmonella enterica subsp. enterica serovar Enteritidis | |
| ERR235130 | Salmonella enterica subsp. enterica serovar Enteritidis | |
| ERR235131 | Salmonella enterica subsp. enterica serovar Enteritidis | |
| ERR235132 | Salmonella enterica subsp. enterica serovar Enteritidis | |
| ERR235133 | Salmonella enterica subsp. enterica serovar Enteritidis | |
| ERR235134 | Salmonella enterica subsp. enterica serovar Enteritidis str. P125109 | |
| ERR235135 | Salmonella enterica subsp. enterica serovar Enteritidis | |
| ERR235136 | Salmonella enterica subsp. enterica serovar Enteritidis str. P125109 | |
| ERR235137 | Salmonella enterica subsp. enterica serovar Enteritidis str. P125109 | |
| ERR235138 | Salmonella enterica subsp. enterica serovar Enteritidis | |
| ERR235139 | Salmonella enterica subsp. enterica serovar Enteritidis | |
| ERR235140 | Salmonella enterica subsp. enterica serovar Enteritidis | |
| ERR235141 | Salmonella enterica subsp. enterica serovar Enteritidis | |
| ERR235142 | Salmonella enterica subsp. enterica serovar Enteritidis | |
| ERR235143 | Salmonella enterica subsp. enterica serovar Enteritidis str. P125109 | |
| ERR235144 | Salmonella enterica subsp. enterica serovar Enteritidis str. P125109 | |
| ERR235145 | Salmonella enterica subsp. enterica serovar Enteritidis | |
| ERR235146 | Salmonella enterica subsp. enterica serovar Enteritidis | |
| ERR235147 | Salmonella enterica subsp. enterica serovar Enteritidis | |
| ERR235148 | Salmonella enterica subsp. enterica serovar Enteritidis | |
| ERR235149 | Salmonella enterica subsp. enterica serovar Enteritidis | |
| ERR235150 | Salmonella enterica subsp. enterica serovar Enteritidis | |
| ERR235151 | Salmonella enterica subsp. enterica serovar Enteritidis | |
| ERR235152 | Salmonella enterica subsp. enterica serovar Enteritidis | |
| ERR235153 | Salmonella enterica subsp. enterica serovar Enteritidis str. P125109 | |
| ERR235154 | Salmonella enterica subsp. enterica serovar Enteritidis | |
| ERR235155 | Salmonella enterica subsp. enterica serovar Enteritidis | |
| ERR235156 | Salmonella enterica subsp. enterica serovar Enteritidis | |
| ERR235157 | Salmonella enterica subsp. enterica serovar Enteritidis str. P125109 | |
| ERR235158 | Salmonella enterica subsp. enterica serovar Enteritidis | |
| ERR235159 | Salmonella enterica subsp. enterica serovar Enteritidis | |
| ERR235160 | Salmonella enterica subsp. enterica serovar Enteritidis | |
| ERR235161 | Salmonella enterica subsp. enterica serovar Enteritidis | |
| ERR235162 | Salmonella enterica subsp. enterica serovar Enteritidis str. P125109 | |
| ERR235163 | Salmonella enterica subsp. enterica serovar Enteritidis | |
| ERR235164 | Salmonella enterica subsp. enterica serovar Enteritidis str. P125109 | |
| ERR235165 | Salmonella enterica subsp. enterica serovar Enteritidis | |
| ERR235166 | Salmonella enterica subsp. enterica serovar Enteritidis | |
| ERR235167 | Salmonella enterica subsp. enterica serovar Enteritidis | |
| ERR235168 | Salmonella enterica subsp. enterica serovar Enteritidis | |
| ERR235169 | Salmonella enterica subsp. enterica serovar Enteritidis | |
| ERR235170 | Salmonella enterica subsp. enterica serovar Enteritidis | |
| ERR235171 | Salmonella enterica subsp. enterica serovar Enteritidis | |
| ERR235172 | Salmonella enterica subsp. enterica serovar Enteritidis str. P125109 | |
| ERR235173 | Salmonella enterica subsp. enterica serovar Enteritidis | |
| ERR235174 | Salmonella enterica subsp. enterica serovar Enteritidis | |
| ERR235175 | Salmonella enterica subsp. enterica serovar Enteritidis | |
| ERR235176 | Salmonella enterica subsp. enterica serovar Enteritidis | |
| ERR235177 | Salmonella enterica subsp. enterica serovar Enteritidis | |
| ERR235178 | Salmonella enterica subsp. enterica serovar Enteritidis | |
| ERR235179 | Salmonella enterica subsp. enterica serovar Enteritidis str. P125109 | |
| ERR235180 | Salmonella enterica subsp. enterica serovar Enteritidis | |
| ERR235181 | Salmonella enterica subsp. enterica serovar Enteritidis | |
| ERR235182 | Salmonella enterica subsp. enterica serovar Enteritidis str. P125109 | |
| ERR235183 | Salmonella enterica subsp. enterica serovar Enteritidis str. P125109 | |
| ERR235184 | Salmonella enterica subsp. enterica serovar Enteritidis | |
| ERR235185 | Salmonella enterica subsp. enterica serovar Enteritidis str. P125109 | |
| ERR235186 | Salmonella enterica subsp. enterica serovar Enteritidis | |
| ERR235187 | Salmonella enterica subsp. enterica serovar Enteritidis str. P125109 | |
| ERR235188 | Salmonella enterica subsp. enterica serovar Enteritidis | |
| ERR235189 | Salmonella enterica subsp. enterica serovar Enteritidis | |
| ERR235190 | Salmonella enterica subsp. enterica serovar Enteritidis | |
| ERR235191 | Salmonella enterica subsp. enterica serovar Enteritidis | |
| ERR235192 | Salmonella enterica subsp. enterica serovar Enteritidis | |
| ERR235193 | Salmonella enterica subsp. enterica serovar Enteritidis | |
| ERR235194 | Salmonella enterica subsp. enterica serovar Enteritidis | |
| ERR235195 | Salmonella enterica subsp. enterica serovar Enteritidis | |
| ERR235196 | Salmonella enterica subsp. enterica serovar Enteritidis | |
| ERR235290 | Salmonella enterica subsp. enterica serovar Enteritidis | |
| ERR235291 | Salmonella enterica subsp. enterica serovar Enteritidis | |
| ERR235292 | Salmonella enterica subsp. enterica serovar Enteritidis | |
| ERR235293 | Salmonella enterica subsp. enterica serovar Enteritidis | |
| ERR235294 | Salmonella enterica subsp. enterica serovar Enteritidis | |
| ERR235295 | Salmonella enterica subsp. enterica serovar Enteritidis | |
| ERR235296 | Salmonella enterica subsp. enterica serovar Enteritidis | |
| ERR235297 | Salmonella enterica subsp. enterica serovar Enteritidis | |
| ERR235298 | Salmonella enterica subsp. enterica serovar Enteritidis | |
| ERR235299 | Salmonella enterica subsp. enterica serovar Enteritidis | |
| ERR235300 | Salmonella enterica subsp. enterica serovar Enteritidis | |
| ERR235301 | Salmonella enterica subsp. enterica serovar Enteritidis | |
| ERR235302 | Salmonella enterica subsp. enterica serovar Enteritidis | |
| ERR235303 | Salmonella enterica subsp. enterica serovar Enteritidis | |
| ERR235304 | Salmonella enterica subsp. enterica serovar Enteritidis | |
| ERR235305 | Salmonella enterica subsp. enterica serovar Enteritidis | |
| ERR235306 | Salmonella enterica subsp. enterica serovar Enteritidis | |
| ERR235307 | Salmonella enterica subsp. enterica serovar Enteritidis | |
| ERR235308 | Salmonella enterica subsp. enterica serovar Enteritidis | |
| ERR235309 | Salmonella enterica subsp. enterica serovar Enteritidis | |
| ERR235310 | Salmonella enterica subsp. enterica serovar Enteritidis | |
| ERR235311 | Salmonella enterica subsp. enterica serovar Enteritidis | |
| ERR235312 | Salmonella enterica subsp. enterica serovar Enteritidis | |
| ERR235313 | Salmonella enterica subsp. enterica serovar Enteritidis | |
| ERR235314 | Salmonella enterica subsp. enterica serovar Enteritidis | |
| ERR235315 | Salmonella enterica subsp. enterica serovar Enteritidis | |
| ERR235316 | Salmonella enterica subsp. enterica serovar Enteritidis | |
| ERR235317 | Salmonella enterica subsp. enterica serovar Enteritidis | |
| ERR235318 | Salmonella enterica subsp. enterica serovar Enteritidis | |
| ERR235319 | Salmonella enterica subsp. enterica serovar Enteritidis | |
| ERR235320 | Salmonella enterica subsp. enterica serovar Enteritidis | |
| ERR235321 | Salmonella enterica subsp. enterica serovar Enteritidis | |
| ERR235322 | Salmonella enterica subsp. enterica serovar Enteritidis | |
| ERR235323 | Salmonella enterica subsp. enterica serovar Enteritidis | |
| ERR235324 | Salmonella enterica subsp. enterica serovar Enteritidis | |
| ERR235325 | Salmonella enterica subsp. enterica serovar Enteritidis | |
| ERR235326 | Salmonella enterica subsp. enterica serovar Enteritidis | |
| ERR235327 | Salmonella enterica subsp. enterica serovar Enteritidis | |
| ERR235328 | Salmonella enterica subsp. enterica serovar Enteritidis | |
| ERR235329 | Salmonella enterica subsp. enterica serovar Enteritidis | |
| ERR235330 | Salmonella enterica subsp. enterica serovar Enteritidis | |
| ERR235331 | Salmonella enterica subsp. enterica serovar Enteritidis str. P125109 | |
| ERR235332 | Salmonella enterica subsp. enterica serovar Enteritidis | |
| ERR235333 | Salmonella enterica subsp. enterica serovar Enteritidis | |
| ERR235334 | Salmonella enterica subsp. enterica serovar Enteritidis | |
| ERR235335 | Salmonella enterica subsp. enterica serovar Enteritidis | |
| ERR235336 | Salmonella enterica subsp. enterica serovar Enteritidis | |
| ERR235337 | Salmonella enterica subsp. enterica serovar Enteritidis | |
| ERR235338 | Salmonella enterica subsp. enterica serovar Enteritidis | |
| ERR235339 | Salmonella enterica subsp. enterica serovar Enteritidis | |
| ERR235340 | Salmonella enterica subsp. enterica serovar Enteritidis | |
| ERR235341 | Salmonella enterica subsp. enterica serovar Enteritidis | |
| ERR235342 | Salmonella enterica subsp. enterica serovar Enteritidis str. P125109 | |
| ERR235343 | Salmonella enterica subsp. enterica serovar Enteritidis | |
| ERR235344 | Salmonella enterica subsp. enterica serovar Enteritidis | |
| ERR235345 | Salmonella enterica subsp. enterica serovar Enteritidis | |
| ERR235346 | Salmonella enterica subsp. enterica serovar Enteritidis | |
| ERR235347 | Salmonella enterica subsp. enterica serovar Enteritidis | |
| ERR235348 | Salmonella enterica subsp. enterica serovar Enteritidis | |
| ERR235349 | Salmonella enterica subsp. enterica serovar Enteritidis | |
| ERR235350 | Salmonella enterica subsp. enterica serovar Enteritidis | |
| ERR235351 | Salmonella enterica subsp. enterica serovar Enteritidis | |
| ERR235352 | Salmonella enterica subsp. enterica serovar Enteritidis | |
| ERR235353 | Salmonella enterica subsp. enterica serovar Enteritidis | |
| ERR235354 | Salmonella enterica subsp. enterica serovar Enteritidis | |
| ERR235355 | Salmonella enterica subsp. enterica serovar Enteritidis | |
| ERR235356 | Salmonella enterica subsp. enterica serovar Enteritidis | |
| ERR235357 | Salmonella enterica subsp. enterica serovar Enteritidis | |
| ERR235358 | Salmonella enterica subsp. enterica serovar Enteritidis | |
| ERR235359 | Salmonella enterica subsp. enterica serovar Enteritidis | |
| ERR235360 | Salmonella enterica subsp. enterica serovar Enteritidis | |
| ERR235361 | Salmonella enterica subsp. enterica serovar Enteritidis | |
| ERR235362 | Salmonella enterica subsp. enterica serovar Enteritidis | |
| ERR235380 | Salmonella enterica subsp. enterica serovar Enteritidis | |
| ERR235384 | Salmonella enterica subsp. enterica serovar Enteritidis | |
| ERR304803 | Salmonella enterica subsp. enterica serovar Enteritidis | |
| ERR304806 | Salmonella enterica subsp. enterica serovar Enteritidis | |
| ERR304817 | Salmonella enterica subsp. enterica serovar Enteritidis | |
| ERR304820 | Salmonella enterica subsp. enterica serovar Enteritidis | |
| ERR304828 | Salmonella enterica subsp. enterica serovar Enteritidis | |
| ERR304829 | Salmonella enterica subsp. enterica serovar Enteritidis | |
| ERR304833 | Salmonella enterica subsp. enterica serovar Enteritidis | |
| ERR304840 | Salmonella enterica subsp. enterica serovar Enteritidis | |
| ERR304845 | Salmonella enterica subsp. enterica serovar Enteritidis | |
| ERR304847 | Salmonella enterica subsp. enterica serovar Enteritidis | |
| ERR304849 | Salmonella enterica subsp. enterica serovar Enteritidis | |
| ERR304851 | Salmonella enterica subsp. enterica serovar Enteritidis | |
| ERR304852 | Salmonella enterica subsp. enterica serovar Enteritidis | |
| ERR304854 | Salmonella enterica subsp. enterica serovar Enteritidis | |
| ERR304864 | Salmonella enterica subsp. enterica serovar Enteritidis | |
| ERR304869 | Salmonella enterica subsp. enterica serovar Enteritidis | |
| ERR305491 | Salmonella enterica subsp. enterica serovar Enteritidis | |
| ERR305492 | Salmonella enterica subsp. enterica serovar Enteritidis | |
| ERR305493 | Salmonella enterica subsp. enterica serovar Enteritidis | |
| ERR305494 | Salmonella enterica subsp. enterica serovar Enteritidis | |
| ERR305495 | Salmonella enterica subsp. enterica serovar Enteritidis | |
| ERR305496 | Salmonella enterica subsp. enterica serovar Enteritidis | |
| ERR305497 | Salmonella enterica subsp. enterica serovar Enteritidis | |
| ERR305498 | Salmonella enterica subsp. enterica serovar Enteritidis | |
| ERR305510 | Salmonella enterica subsp. enterica serovar Enteritidis | |
| ERR305511 | Salmonella enterica subsp. enterica serovar Enteritidis | |
| ERR311254 | Salmonella enterica subsp. enterica serovar Enteritidis | |
| ERR311255 | Salmonella enterica subsp. enterica serovar Enteritidis | |
| ERR311256 | Salmonella enterica subsp. enterica serovar Enteritidis | |
| ERR311257 | Salmonella enterica subsp. enterica serovar Enteritidis | |
| ERR311258 | Salmonella enterica subsp. enterica serovar Enteritidis | |
| ERR311259 | Salmonella enterica subsp. enterica serovar Enteritidis | |
| ERR311260 | Salmonella enterica subsp. enterica serovar Enteritidis | |
| ERR311261 | Salmonella enterica subsp. enterica serovar Enteritidis | |
| ERR311262 | Salmonella enterica subsp. enterica serovar Enteritidis | |
| ERR311263 | Salmonella enterica subsp. enterica serovar Enteritidis | |
| ERR311264 | Salmonella enterica subsp. enterica serovar Enteritidis | |
| ERR311265 | Salmonella enterica subsp. enterica serovar Enteritidis | |
| ERR311266 | Salmonella enterica subsp. enterica serovar Enteritidis | |
| ERR311267 | Salmonella enterica subsp. enterica serovar Enteritidis | |
| ERR311268 | Salmonella enterica subsp. enterica serovar Enteritidis | |
| ERR311269 | Salmonella enterica subsp. enterica serovar Enteritidis | |
| ERR311270 | Salmonella enterica subsp. enterica serovar Enteritidis | |
| ERR311271 | Salmonella enterica subsp. enterica serovar Enteritidis | |
| ERR311272 | Salmonella enterica subsp. enterica serovar Enteritidis | |
| ERR311273 | Salmonella enterica subsp. enterica serovar Enteritidis | |
| ERR311274 | Salmonella enterica subsp. enterica serovar Enteritidis | |
| ERR311275 | Salmonella enterica subsp. enterica serovar Enteritidis | |
| ERR311276 | Salmonella enterica subsp. enterica serovar Enteritidis | |
| ERR311277 | Salmonella enterica subsp. enterica serovar Enteritidis | |
| ERR311278 | Salmonella enterica subsp. enterica serovar Enteritidis | |
| ERR311279 | Salmonella enterica subsp. enterica serovar Enteritidis | |
| ERR311280 | Salmonella enterica subsp. enterica serovar Enteritidis | |
| ERR311281 | Salmonella enterica subsp. enterica serovar Enteritidis | |
| ERR311282 | Salmonella enterica subsp. enterica serovar Enteritidis | |
| ERR311283 | Salmonella enterica subsp. enterica serovar Enteritidis | |
| ERR311284 | Salmonella enterica subsp. enterica serovar Enteritidis | |
| ERR311285 | Salmonella enterica subsp. enterica serovar Enteritidis | |
| ERR311286 | Salmonella enterica subsp. enterica serovar Enteritidis | |
| ERR311287 | Salmonella enterica subsp. enterica serovar Enteritidis | |
| ERR311288 | Salmonella enterica subsp. enterica serovar Enteritidis | |
| ERR311289 | Salmonella enterica subsp. enterica serovar Enteritidis | |
| ERR311290 | Salmonella enterica subsp. enterica serovar Enteritidis | |
| ERR311291 | Salmonella enterica subsp. enterica serovar Enteritidis | |
| ERR311292 | Salmonella enterica subsp. enterica serovar Enteritidis | |
| ERR311293 | Salmonella enterica subsp. enterica serovar Enteritidis | |
| ERR311294 | Salmonella enterica subsp. enterica serovar Enteritidis | |
| ERR311295 | Salmonella enterica subsp. enterica serovar Enteritidis | |
| ERR311296 | Salmonella enterica subsp. enterica serovar Enteritidis | |
| ERR311297 | Salmonella enterica subsp. enterica serovar Enteritidis | |
| ERR311298 | Salmonella enterica subsp. enterica serovar Enteritidis | |
| ERR311299 | Salmonella enterica subsp. enterica serovar Enteritidis | |
| ERR311300 | Salmonella enterica subsp. enterica serovar Enteritidis | |
| ERR311301 | Salmonella enterica subsp. enterica serovar Enteritidis | |
| ERR311302 | Salmonella enterica subsp. enterica serovar Enteritidis | |
| ERR311303 | Salmonella enterica subsp. enterica serovar Enteritidis | |
| ERR311304 | Salmonella enterica subsp. enterica serovar Enteritidis | |
| ERR311305 | Salmonella enterica subsp. enterica serovar Enteritidis | |
| ERR311306 | Salmonella enterica subsp. enterica serovar Enteritidis | |
| ERR311307 | Salmonella enterica subsp. enterica serovar Enteritidis | |
| ERR311308 | Salmonella enterica subsp. enterica serovar Enteritidis | |
| ERR311309 | Salmonella enterica subsp. enterica serovar Enteritidis | |
| ERR311310 | Salmonella enterica subsp. enterica serovar Enteritidis | |
| ERR311311 | Salmonella enterica subsp. enterica serovar Enteritidis | |
| ERR317020 | Salmonella enterica subsp. enterica serovar Enteritidis | |
| ERR317021 | Salmonella enterica subsp. enterica serovar Enteritidis | |
| ERR317022 | Salmonella enterica subsp. enterica serovar Enteritidis | |
| ERR317023 | Salmonella enterica subsp. enterica serovar Enteritidis | |
| ERR317024 | Salmonella enterica subsp. enterica serovar Enteritidis | |
| ERR317025 | Salmonella enterica subsp. enterica serovar Enteritidis | |
| ERR317026 | Salmonella enterica subsp. enterica serovar Enteritidis | |
| ERR317027 | Salmonella enterica subsp. enterica serovar Enteritidis | |
| ERR317028 | Salmonella enterica subsp. enterica serovar Enteritidis | |
| ERR317029 | Salmonella enterica subsp. enterica serovar Enteritidis | |
| ERR317030 | Salmonella enterica subsp. enterica serovar Enteritidis | |
| ERR317031 | Salmonella enterica subsp. enterica serovar Enteritidis | |
| ERR317032 | Salmonella enterica subsp. enterica serovar Enteritidis | |
| ERR317033 | Salmonella enterica subsp. enterica serovar Enteritidis | |
| ERR317034 | Salmonella enterica subsp. enterica serovar Enteritidis | |
| ERR317035 | Salmonella enterica subsp. enterica serovar Enteritidis | |
| ERR317036 | Salmonella enterica subsp. enterica serovar Enteritidis | |
| ERR317037 | Salmonella enterica subsp. enterica serovar Enteritidis | |
| ERR317038 | Salmonella enterica subsp. enterica serovar Enteritidis | |
| ERR317039 | Salmonella enterica subsp. enterica serovar Enteritidis | |
| ERR317040 | Salmonella enterica subsp. enterica serovar Enteritidis | |
| ERR317041 | Salmonella enterica subsp. enterica serovar Enteritidis | |
| ERR317042 | Salmonella enterica subsp. enterica serovar Enteritidis | |
| ERR317043 | Salmonella enterica subsp. enterica serovar Enteritidis | |
| ERR317044 | Salmonella enterica subsp. enterica serovar Enteritidis | |
| ERR317045 | Salmonella enterica subsp. enterica serovar Enteritidis | |
| ERR317046 | Salmonella enterica subsp. enterica serovar Enteritidis | |
| ERR317047 | Salmonella enterica subsp. enterica serovar Enteritidis | |
| ERR317048 | Salmonella enterica subsp. enterica serovar Enteritidis | |
| ERR317049 | Salmonella enterica subsp. enterica serovar Enteritidis | |
| ERR317050 | Salmonella enterica subsp. enterica serovar Enteritidis | |
| ERR317051 | Salmonella enterica subsp. enterica serovar Enteritidis | |
| ERR317052 | Salmonella enterica subsp. enterica serovar Enteritidis | |
| ERR317053 | Salmonella enterica subsp. enterica serovar Enteritidis | |
| ERR317054 | Salmonella enterica subsp. enterica serovar Enteritidis | |
| ERR317055 | Salmonella enterica subsp. enterica serovar Enteritidis | |
| ERR317056 | Salmonella enterica subsp. enterica serovar Enteritidis | |
| ERR317057 | Salmonella enterica subsp. enterica serovar Enteritidis | |
| ERR317058 | Salmonella enterica subsp. enterica serovar Enteritidis | |
| ERR317059 | Salmonella enterica subsp. enterica serovar Enteritidis | |
| ERR317060 | Salmonella enterica subsp. enterica serovar Enteritidis | |
| ERR317061 | Salmonella enterica subsp. enterica serovar Enteritidis | |
| ERR317062 | Salmonella enterica subsp. enterica serovar Enteritidis | |
| ERR317063 | Salmonella enterica subsp. enterica serovar Enteritidis | |
| ERR317064 | Salmonella enterica subsp. enterica serovar Enteritidis | |
| ERR317065 | Salmonella enterica subsp. enterica serovar Enteritidis | |
| ERR317066 | Salmonella enterica subsp. enterica serovar Enteritidis | |
| ERR317067 | Salmonella enterica subsp. enterica serovar Enteritidis | |
| ERR317068 | Salmonella enterica subsp. enterica serovar Enteritidis | |
| ERR317069 | Salmonella enterica subsp. enterica serovar Enteritidis | |
| ERR317070 | Salmonella enterica subsp. enterica serovar Enteritidis | |
| ERR317071 | Salmonella enterica subsp. enterica serovar Enteritidis | |
| ERR317072 | Salmonella enterica subsp. enterica serovar Enteritidis | |
| ERR317073 | Salmonella enterica subsp. enterica serovar Enteritidis | |
| ERR317074 | Salmonella enterica subsp. enterica serovar Enteritidis | |
| ERR317075 | Salmonella enterica subsp. enterica serovar Enteritidis | |
| ERR317076 | Salmonella enterica subsp. enterica serovar Enteritidis | |
| ERR317077 | Salmonella enterica subsp. enterica serovar Enteritidis | |
| ERR317078 | Salmonella enterica subsp. enterica serovar Enteritidis | |
| ERR338252 | Salmonella enterica subsp. enterica serovar Enteritidis | |
| ERR338253 | Salmonella enterica subsp. enterica serovar Enteritidis | |
| ERR338254 | Salmonella enterica subsp. enterica serovar Enteritidis | |
| ERR338255 | Salmonella enterica subsp. enterica serovar Enteritidis | |
| ERR338256 | Salmonella enterica subsp. enterica serovar Enteritidis | |
| ERR338257 | Salmonella enterica subsp. enterica serovar Enteritidis | |
| ERR338258 | Salmonella enterica subsp. enterica serovar Enteritidis | |
| ERR338259 | Salmonella enterica subsp. enterica serovar Enteritidis | |
| ERR338260 | Salmonella enterica subsp. enterica serovar Enteritidis | |
| ERR338261 | Salmonella enterica subsp. enterica serovar Enteritidis | |
| ERR338262 | Salmonella enterica subsp. enterica serovar Enteritidis | |
| ERR338263 | Salmonella enterica subsp. enterica serovar Enteritidis | |
| ERR338264 | Salmonella enterica subsp. enterica serovar Enteritidis | |
| ERR338265 | Salmonella enterica subsp. enterica serovar Enteritidis | |
| ERR338266 | Salmonella enterica subsp. enterica serovar Enteritidis | |
| ERR338267 | Salmonella enterica subsp. enterica serovar Enteritidis | |
| ERR338268 | Salmonella enterica subsp. enterica serovar Enteritidis | |
| ERR338269 | Salmonella enterica subsp. enterica serovar Enteritidis | |
| ERR338270 | Salmonella enterica subsp. enterica serovar Enteritidis | |
| ERR338271 | Salmonella enterica subsp. enterica serovar Enteritidis | |
| ERR338272 | Salmonella enterica subsp. enterica serovar Enteritidis | |
| ERR338273 | Salmonella enterica subsp. enterica serovar Enteritidis | |
| ERR338274 | Salmonella enterica subsp. enterica serovar Enteritidis | |
| ERR338275 | Salmonella enterica subsp. enterica serovar Enteritidis | |
| ERR338276 | Salmonella enterica subsp. enterica serovar Enteritidis | |
| ERR338277 | Salmonella enterica subsp. enterica serovar Enteritidis | |
| ERR338278 | Salmonella enterica subsp. enterica serovar Enteritidis | |
| ERR338279 | Salmonella enterica subsp. enterica serovar Enteritidis | |
| ERR338280 | Salmonella enterica subsp. enterica serovar Enteritidis | |
| ERR338281 | Salmonella enterica subsp. enterica serovar Enteritidis | |
| ERR338282 | Salmonella enterica subsp. enterica serovar Enteritidis | |
| ERR338283 | Salmonella enterica subsp. enterica serovar Enteritidis | |
| ERR338284 | Salmonella enterica subsp. enterica serovar Enteritidis | |
| ERR338285 | Salmonella enterica subsp. enterica serovar Enteritidis | |
| ERR338286 | Salmonella enterica subsp. enterica serovar Enteritidis | |
| ERR338287 | Salmonella enterica subsp. enterica serovar Enteritidis | |
| ERR338288 | Salmonella enterica subsp. enterica serovar Enteritidis | |
| ERR338289 | Salmonella enterica subsp. enterica serovar Enteritidis | |
| ERR338290 | Salmonella enterica subsp. enterica serovar Enteritidis | |
| ERR338291 | Salmonella enterica subsp. enterica serovar Enteritidis | |
| ERR338292 | Salmonella enterica subsp. enterica serovar Enteritidis | |
| ERR338293 | Salmonella enterica subsp. enterica serovar Enteritidis | |
| ERR338294 | Salmonella enterica subsp. enterica serovar Enteritidis | |
| ERR338295 | Salmonella enterica subsp. enterica serovar Enteritidis | |
| ERR338296 | Salmonella enterica subsp. enterica serovar Enteritidis | |
| ERR338297 | Salmonella enterica subsp. enterica serovar Enteritidis | |
| ERR338298 | Salmonella enterica subsp. enterica serovar Enteritidis | |
| ERR338299 | Salmonella enterica subsp. enterica serovar Enteritidis | |
| ERR338300 | Salmonella enterica subsp. enterica serovar Enteritidis | |
| ERR338301 | Salmonella enterica subsp. enterica serovar Enteritidis | |
| ERR338302 | Salmonella enterica subsp. enterica serovar Enteritidis | |
| ERR338303 | Salmonella enterica subsp. enterica serovar Enteritidis | |
| ERR338304 | Salmonella enterica subsp. enterica serovar Enteritidis | |
| ERR338305 | Salmonella enterica subsp. enterica serovar Enteritidis | |
| ERR338306 | Salmonella enterica subsp. enterica serovar Enteritidis | |
| ERR338307 | Salmonella enterica subsp. enterica serovar Enteritidis | |
| ERR338308 | Salmonella enterica subsp. enterica serovar Enteritidis | |
| ERR338309 | Salmonella enterica subsp. enterica serovar Enteritidis | |
| ERR338310 | Salmonella enterica subsp. enterica serovar Enteritidis | |
| ERR338311 | Salmonella enterica subsp. enterica serovar Enteritidis | |
| ERR338312 | Salmonella enterica subsp. enterica serovar Enteritidis | |
| ERR338313 | Salmonella enterica subsp. enterica serovar Enteritidis | |
| ERR338314 | Salmonella enterica subsp. enterica serovar Enteritidis | |
| ERR338315 | Salmonella enterica subsp. enterica serovar Enteritidis | |
| ERR338316 | Salmonella enterica subsp. enterica serovar Enteritidis | |
| ERR338317 | Salmonella enterica subsp. enterica serovar Enteritidis | |
| ERR338318 | Salmonella enterica subsp. enterica serovar Enteritidis | |
| ERR338905 | Salmonella enterica subsp. enterica serovar Enteritidis | |
| ERR338906 | Salmonella enterica subsp. enterica serovar Enteritidis | |
| ERR338907 | Salmonella enterica subsp. enterica serovar Enteritidis | |
| ERR338908 | Salmonella enterica subsp. enterica serovar Enteritidis | |
| ERR338909 | Salmonella enterica subsp. enterica serovar Enteritidis | |
| ERR338910 | Salmonella enterica subsp. enterica serovar Enteritidis | |
| ERR338911 | Salmonella enterica subsp. enterica serovar Enteritidis | |
| ERR338912 | Salmonella enterica subsp. enterica serovar Enteritidis | |
| ERR338913 | Salmonella enterica subsp. enterica serovar Enteritidis | |
| ERR338914 | Salmonella enterica subsp. enterica serovar Enteritidis | |
| ERR338915 | Salmonella enterica subsp. enterica serovar Enteritidis | |
| ERR338916 | Salmonella enterica subsp. enterica serovar Enteritidis | |
| ERR338917 | Salmonella enterica subsp. enterica serovar Enteritidis | |
| ERR338918 | Salmonella enterica subsp. enterica serovar Enteritidis | |
| ERR338919 | Salmonella enterica subsp. enterica serovar Enteritidis | |
| ERR338920 | Salmonella enterica subsp. enterica serovar Enteritidis | |
| ERR338921 | Salmonella enterica subsp. enterica serovar Enteritidis | |
| ERR338922 | Salmonella enterica subsp. enterica serovar Enteritidis | |
| ERR338923 | Salmonella enterica subsp. enterica serovar Enteritidis | |
| ERR338924 | Salmonella enterica subsp. enterica serovar Enteritidis | |
| ERR338925 | Salmonella enterica subsp. enterica serovar Enteritidis | |
| ERR338926 | Salmonella enterica subsp. enterica serovar Enteritidis | |
| ERR338927 | Salmonella enterica subsp. enterica serovar Enteritidis | |
| ERR338928 | Salmonella enterica subsp. enterica serovar Enteritidis | |
| ERR338929 | Salmonella enterica subsp. enterica serovar Enteritidis | |
| ERR338930 | Salmonella enterica subsp. enterica serovar Enteritidis | |
| ERR338931 | Salmonella enterica subsp. enterica serovar Enteritidis | |
| ERR338932 | Salmonella enterica subsp. enterica serovar Enteritidis | |
| ERR338933 | Salmonella enterica subsp. enterica serovar Enteritidis | |
| ERR338934 | Salmonella enterica subsp. enterica serovar Enteritidis | |
| ERR338935 | Salmonella enterica subsp. enterica serovar Enteritidis | |
| ERR338936 | Salmonella enterica subsp. enterica serovar Enteritidis | |
| ERR338937 | Salmonella enterica subsp. enterica serovar Enteritidis | |
| ERR338938 | Salmonella enterica subsp. enterica serovar Enteritidis | |
| ERR338939 | Salmonella enterica subsp. enterica serovar Enteritidis | |
| ERR338940 | Salmonella enterica subsp. enterica serovar Enteritidis | |
| ERR338941 | Salmonella enterica subsp. enterica serovar Enteritidis | |
| ERR338942 | Salmonella enterica subsp. enterica serovar Enteritidis | |
| ERR338943 | Salmonella enterica subsp. enterica serovar Enteritidis | |
| ERR338944 | Salmonella enterica subsp. enterica serovar Enteritidis | |
| ERR338945 | Salmonella enterica subsp. enterica serovar Enteritidis | |
| ERR338946 | Salmonella enterica subsp. enterica serovar Enteritidis | |
| ERR338947 | Salmonella enterica subsp. enterica serovar Enteritidis | |
| ERR338948 | Salmonella enterica subsp. enterica serovar Enteritidis | |
| ERR338949 | Salmonella enterica subsp. enterica serovar Enteritidis | |
| ERR338950 | Salmonella enterica subsp. enterica serovar Enteritidis | |
| ERR338951 | Salmonella enterica subsp. enterica serovar Enteritidis | |
| ERR338952 | Salmonella enterica subsp. enterica serovar Enteritidis | |
| ERR338953 | Salmonella enterica subsp. enterica serovar Enteritidis | |
| ERR338954 | Salmonella enterica subsp. enterica serovar Enteritidis | |
| ERR338955 | Salmonella enterica subsp. enterica serovar Enteritidis | |
| ERR338956 | Salmonella enterica subsp. enterica serovar Enteritidis | |
| ERR338957 | Salmonella enterica subsp. enterica serovar Enteritidis | |
| ERR338958 | Salmonella enterica subsp. enterica serovar Enteritidis | |
| ERR338959 | Salmonella enterica subsp. enterica serovar Enteritidis | |
| ERR338960 | Salmonella enterica subsp. enterica serovar Enteritidis | |
| ERR338961 | Salmonella enterica subsp. enterica serovar Enteritidis | |
| ERR338962 | Salmonella enterica subsp. enterica serovar Enteritidis | |
| ERR338963 | Salmonella enterica subsp. enterica serovar Enteritidis | |
| ERR338964 | Salmonella enterica subsp. enterica serovar Enteritidis | |
| ERR338965 | Salmonella enterica subsp. enterica serovar Enteritidis | |
| ERR338966 | Salmonella enterica subsp. enterica serovar Enteritidis | |
| ERR338967 | Salmonella enterica subsp. enterica serovar Enteritidis | |
| ERR338968 | Salmonella enterica subsp. enterica serovar Enteritidis | |
| ERR338969 | Salmonella enterica subsp. enterica serovar Enteritidis | |
| ERR338970 | Salmonella enterica subsp. enterica serovar Enteritidis | |
| ERR338971 | Salmonella enterica subsp. enterica serovar Enteritidis | |
| ERR341097 | Salmonella enterica subsp. enterica serovar Enteritidis | |
| ERR341098 | Salmonella enterica subsp. enterica serovar Enteritidis | |
| ERR341099 | Salmonella enterica subsp. enterica serovar Enteritidis | |
| ERR341100 | Salmonella enterica subsp. enterica serovar Enteritidis | |
| ERR341101 | Salmonella enterica subsp. enterica serovar Enteritidis | |
| ERR341102 | Salmonella enterica subsp. enterica serovar Enteritidis | |
| ERR341103 | Salmonella enterica subsp. enterica serovar Enteritidis | |
| ERR352120 | Salmonella enterica subsp. enterica serovar Enteritidis | |
| ERR352121 | Salmonella enterica subsp. enterica serovar Enteritidis | |
| ERR352122 | Salmonella enterica subsp. enterica serovar Enteritidis | |
| ERR352173 | Salmonella enterica subsp. enterica serovar Enteritidis | |
| ERR353344 | Salmonella enterica subsp. enterica serovar Enteritidis | |
| ERR353345 | Salmonella enterica subsp. enterica serovar Enteritidis | |
| ERR353346 | Salmonella enterica subsp. enterica serovar Enteritidis | |
| ERR353347 | Salmonella enterica subsp. enterica serovar Enteritidis | |
| ERR353348 | Salmonella enterica subsp. enterica serovar Enteritidis | |
| ERR353349 | Salmonella enterica subsp. enterica serovar Enteritidis | |
| ERR353350 | Salmonella enterica subsp. enterica serovar Enteritidis | |
| ERR353351 | Salmonella enterica subsp. enterica serovar Enteritidis | |
| ERR357430 | Salmonella enterica subsp. enterica serovar Enteritidis | |
| ERR357431 | Salmonella enterica subsp. enterica serovar Enteritidis | |
| ERR357432 | Salmonella enterica subsp. enterica serovar Enteritidis | |
| ERR357433 | Salmonella enterica subsp. enterica serovar Enteritidis | |
| ERR357434 | Salmonella enterica subsp. enterica serovar Enteritidis | |
| ERR357435 | Salmonella enterica subsp. enterica serovar Enteritidis | |
| ERR357436 | Salmonella enterica subsp. enterica serovar Enteritidis | |
| ERR357437 | Salmonella enterica subsp. enterica serovar Enteritidis | |
| ERR369341 | Salmonella enterica subsp. enterica serovar Enteritidis | |
| ERR369342 | Salmonella enterica subsp. enterica serovar Enteritidis | |
| ERR369343 | Salmonella enterica subsp. enterica serovar Enteritidis | |
| ERR369344 | Salmonella enterica subsp. enterica serovar Enteritidis | |
| ERR369345 | Salmonella enterica subsp. enterica serovar Enteritidis | |
| ERR369346 | Salmonella enterica subsp. enterica serovar Enteritidis | |
| ERR369347 | Salmonella enterica subsp. enterica serovar Enteritidis | |
| ERR369348 | Salmonella enterica subsp. enterica serovar Enteritidis | |
| ERR369349 | Salmonella enterica subsp. enterica serovar Enteritidis | |
| ERR369350 | Salmonella enterica subsp. enterica serovar Enteritidis | |
| ERR369351 | Salmonella enterica subsp. enterica serovar Enteritidis | |
| ERR369352 | Salmonella enterica subsp. enterica serovar Enteritidis | |
| ERR369353 | Salmonella enterica subsp. enterica serovar Enteritidis | |
| ERR369354 | Salmonella enterica subsp. enterica serovar Enteritidis | |
| ERR369355 | Salmonella enterica subsp. enterica serovar Enteritidis | |
| ERR369356 | Salmonella enterica subsp. enterica serovar Enteritidis | |
| ERR369357 | Salmonella enterica subsp. enterica serovar Enteritidis | |
| ERR369358 | Salmonella enterica subsp. enterica serovar Enteritidis | |
| ERR369359 | Salmonella enterica subsp. enterica serovar Enteritidis | |
| ERR369360 | Salmonella enterica subsp. enterica serovar Enteritidis | |
| ERR369361 | Salmonella enterica subsp. enterica serovar Enteritidis | |
| ERR369362 | Salmonella enterica subsp. enterica serovar Enteritidis | |
| ERR369363 | Salmonella enterica subsp. enterica serovar Enteritidis | |
| ERR369364 | Salmonella enterica subsp. enterica serovar Enteritidis | |
| ERR369365 | Salmonella enterica subsp. enterica serovar Enteritidis | |
| ERR369366 | Salmonella enterica subsp. enterica serovar Enteritidis | |
| ERR369367 | Salmonella enterica subsp. enterica serovar Enteritidis | |
| ERR369368 | Salmonella enterica subsp. enterica serovar Enteritidis | |
| ERR369369 | Salmonella enterica subsp. enterica serovar Enteritidis | |
| ERR369370 | Salmonella enterica subsp. enterica serovar Enteritidis | |
| ERR369371 | Salmonella enterica subsp. enterica serovar Enteritidis | |
| ERR369372 | Salmonella enterica subsp. enterica serovar Enteritidis | |
| ERR369373 | Salmonella enterica subsp. enterica serovar Enteritidis | |
| ERR369374 | Salmonella enterica subsp. enterica serovar Enteritidis | |
| ERR369375 | Salmonella enterica subsp. enterica serovar Enteritidis | |
| ERR369376 | Salmonella enterica subsp. enterica serovar Enteritidis | |
| ERR369377 | Salmonella enterica subsp. enterica serovar Enteritidis | |
| ERR369378 | Salmonella enterica subsp. enterica serovar Enteritidis | |
| ERR369379 | Salmonella enterica subsp. enterica serovar Enteritidis | |
| ERR369380 | Salmonella enterica subsp. enterica serovar Enteritidis | |
| ERR369381 | Salmonella enterica subsp. enterica serovar Enteritidis | |
| ERR369382 | Salmonella enterica subsp. enterica serovar Enteritidis | |
| ERR369383 | Salmonella enterica subsp. enterica serovar Enteritidis | |
| ERR369384 | Salmonella enterica subsp. enterica serovar Enteritidis | |
| ERR369385 | Salmonella enterica subsp. enterica serovar Enteritidis | |
| ERR369386 | Salmonella enterica subsp. enterica serovar Enteritidis | |
| ERR369387 | Salmonella enterica subsp. enterica serovar Enteritidis | |
| ERR369388 | Salmonella enterica subsp. enterica serovar Enteritidis | |
| ERR369389 | Salmonella enterica subsp. enterica serovar Enteritidis | |
| ERR369390 | Salmonella enterica subsp. enterica serovar Enteritidis | |
| ERR369391 | Salmonella enterica subsp. enterica serovar Enteritidis | |
| ERR369392 | Salmonella enterica subsp. enterica serovar Enteritidis | |
| ERR369393 | Salmonella enterica subsp. enterica serovar Enteritidis | |
| ERR369394 | Salmonella enterica subsp. enterica serovar Enteritidis | |
| ERR369395 | Salmonella enterica subsp. enterica serovar Enteritidis | |
| ERR369396 | Salmonella enterica subsp. enterica serovar Enteritidis | |
| ERR369397 | Salmonella enterica subsp. enterica serovar Enteritidis | |
| ERR369398 | Salmonella enterica subsp. enterica serovar Enteritidis | |
| ERR369399 | Salmonella enterica subsp. enterica serovar Enteritidis | |
| ERR369400 | Salmonella enterica subsp. enterica serovar Enteritidis | |
| ERR369401 | Salmonella enterica subsp. enterica serovar Enteritidis | |
| ERR369402 | Salmonella enterica subsp. enterica serovar Enteritidis | |
| ERR369403 | Salmonella enterica subsp. enterica serovar Enteritidis | |
| ERR369404 | Salmonella enterica subsp. enterica serovar Enteritidis | |
| ERR369405 | Salmonella enterica subsp. enterica serovar Enteritidis | |
| ERR369406 | Salmonella enterica subsp. enterica serovar Enteritidis | |
| ERR369407 | Salmonella enterica subsp. enterica serovar Enteritidis | |
| ERR369408 | Salmonella enterica subsp. enterica serovar Enteritidis | |
| ERR369409 | Salmonella enterica subsp. enterica serovar Enteritidis | |
| ERR369410 | Salmonella enterica subsp. enterica serovar Enteritidis | |
| ERR369411 | Salmonella enterica subsp. enterica serovar Enteritidis | |
| ERR369412 | Salmonella enterica subsp. enterica serovar Enteritidis | |
| ERR369413 | Salmonella enterica subsp. enterica serovar Enteritidis | |
| ERR369414 | Salmonella enterica subsp. enterica serovar Enteritidis | |
| ERR369415 | Salmonella enterica subsp. enterica serovar Enteritidis | |
| ERR369416 | Salmonella enterica subsp. enterica serovar Enteritidis | |
| ERR369417 | Salmonella enterica subsp. enterica serovar Enteritidis | |
| ERR369418 | Salmonella enterica subsp. enterica serovar Enteritidis | |
| ERR369419 | Salmonella enterica subsp. enterica serovar Enteritidis | |
| ERR369420 | Salmonella enterica subsp. enterica serovar Enteritidis | |
| ERR369421 | Salmonella enterica subsp. enterica serovar Enteritidis | |
| ERR374207 | Salmonella enterica subsp. enterica serovar Enteritidis | |
| ERR374208 | Salmonella enterica subsp. enterica serovar Enteritidis | |
| ERR374209 | Salmonella enterica subsp. enterica serovar Enteritidis | |
| ERR374210 | Salmonella enterica subsp. enterica serovar Enteritidis | |
| ERR374211 | Salmonella enterica subsp. enterica serovar Enteritidis | |
| ERR374212 | Salmonella enterica subsp. enterica serovar Enteritidis | |
| ERR374213 | Salmonella enterica subsp. enterica serovar Enteritidis | |
| ERR374214 | Salmonella enterica subsp. enterica serovar Enteritidis | |
| ERR374215 | Salmonella enterica subsp. enterica serovar Enteritidis | |
| ERR374216 | Salmonella enterica subsp. enterica serovar Enteritidis | |
| ERR374217 | Salmonella enterica subsp. enterica serovar Enteritidis | |
| ERR374218 | Salmonella enterica subsp. enterica serovar Enteritidis | |
| ERR374219 | Salmonella enterica subsp. enterica serovar Enteritidis | |
| ERR374220 | Salmonella enterica subsp. enterica serovar Enteritidis | |
| ERR374221 | Salmonella enterica subsp. enterica serovar Enteritidis | |
| ERR374222 | Salmonella enterica subsp. enterica serovar Enteritidis | |
| ERR374223 | Salmonella enterica subsp. enterica serovar Enteritidis | |
| ERR374224 | Salmonella enterica subsp. enterica serovar Enteritidis | |
| ERR374225 | Salmonella enterica subsp. enterica serovar Enteritidis | |
| ERR374226 | Salmonella enterica subsp. enterica serovar Enteritidis | |
| ERR374227 | Salmonella enterica subsp. enterica serovar Enteritidis | |
| ERR374228 | Salmonella enterica subsp. enterica serovar Enteritidis | |
| ERR374229 | Salmonella enterica subsp. enterica serovar Enteritidis | |
| ERR374230 | Salmonella enterica subsp. enterica serovar Enteritidis | |
| ERR374231 | Salmonella enterica subsp. enterica serovar Enteritidis | |
| ERR374232 | Salmonella enterica subsp. enterica serovar Enteritidis | |
| ERR374233 | Salmonella enterica subsp. enterica serovar Enteritidis | |
| ERR374234 | Salmonella enterica subsp. enterica serovar Enteritidis | |
| ERR374235 | Salmonella enterica subsp. enterica serovar Enteritidis | |
| ERR374236 | Salmonella enterica subsp. enterica serovar Enteritidis | |
| ERR374237 | Salmonella enterica subsp. enterica serovar Enteritidis | |
| ERR374238 | Salmonella enterica subsp. enterica serovar Enteritidis | |
| ERR374239 | Salmonella enterica subsp. enterica serovar Enteritidis | |
| ERR374240 | Salmonella enterica subsp. enterica serovar Enteritidis | |
| ERR374241 | Salmonella enterica subsp. enterica serovar Enteritidis | |
| ERR374242 | Salmonella enterica subsp. enterica serovar Enteritidis | |
| ERR374243 | Salmonella enterica subsp. enterica serovar Enteritidis | |
| ERR374244 | Salmonella enterica subsp. enterica serovar Enteritidis | |
| ERR374245 | Salmonella enterica subsp. enterica serovar Enteritidis | |
| ERR374246 | Salmonella enterica subsp. enterica serovar Enteritidis | |
| ERR374247 | Salmonella enterica subsp. enterica serovar Enteritidis | |
| ERR374248 | Salmonella enterica subsp. enterica serovar Enteritidis | |
| ERR387650 | Salmonella enterica subsp. enterica serovar Enteritidis | |
| ERR387651 | Salmonella enterica subsp. enterica serovar Enteritidis | |
| ERR387653 | Salmonella enterica subsp. enterica serovar Enteritidis | |
| ERR387654 | Salmonella enterica subsp. enterica serovar Enteritidis | |
| ERR387658 | Salmonella enterica subsp. enterica serovar Enteritidis | |
| ERR387659 | Salmonella enterica subsp. enterica serovar Enteritidis | |
| ERR387660 | Salmonella enterica subsp. enterica serovar Enteritidis | |
| ERR387661 | Salmonella enterica subsp. enterica serovar Enteritidis | |
| ERR387662 | Salmonella enterica subsp. enterica serovar Enteritidis | |
| ERR387663 | Salmonella enterica subsp. enterica serovar Enteritidis | |
| ERR387664 | Salmonella enterica subsp. enterica serovar Enteritidis | |
| ERR387665 | Salmonella enterica subsp. enterica serovar Enteritidis | |
| ERR387666 | Salmonella enterica subsp. enterica serovar Enteritidis | |
| ERR387667 | Salmonella enterica subsp. enterica serovar Enteritidis | |
| ERR387668 | Salmonella enterica subsp. enterica serovar Enteritidis | |
| ERR387669 | Salmonella enterica subsp. enterica serovar Enteritidis | |
| ERR387670 | Salmonella enterica subsp. enterica serovar Enteritidis | |
| ERR387671 | Salmonella enterica subsp. enterica serovar Enteritidis | |
| ERR387672 | Salmonella enterica subsp. enterica serovar Enteritidis | |
| ERR387673 | Salmonella enterica subsp. enterica serovar Enteritidis | |
| ERR387674 | Salmonella enterica subsp. enterica serovar Enteritidis | |
| ERR387675 | Salmonella enterica subsp. enterica serovar Enteritidis | |
| ERR387676 | Salmonella enterica subsp. enterica serovar Enteritidis | |
| ERR387677 | Salmonella enterica subsp. enterica serovar Enteritidis | |
| ERR387678 | Salmonella enterica subsp. enterica serovar Enteritidis | |
| ERR387679 | Salmonella enterica subsp. enterica serovar Enteritidis | |
| ERR387680 | Salmonella enterica subsp. enterica serovar Enteritidis | |
| ERR387681 | Salmonella enterica subsp. enterica serovar Enteritidis | |
| ERR387682 | Salmonella enterica subsp. enterica serovar Enteritidis | |
| ERR387683 | Salmonella enterica subsp. enterica serovar Enteritidis | |
| ERR387684 | Salmonella enterica subsp. enterica serovar Enteritidis | |
| ERR387685 | Salmonella enterica subsp. enterica serovar Enteritidis | |
| ERR387686 | Salmonella enterica subsp. enterica serovar Enteritidis | |
| ERR387687 | Salmonella enterica subsp. enterica serovar Enteritidis | |
| ERR387688 | Salmonella enterica subsp. enterica serovar Enteritidis | |
| ERR387689 | Salmonella enterica subsp. enterica serovar Enteritidis | |
| ERR387690 | Salmonella enterica subsp. enterica serovar Enteritidis | |
| ERR387691 | Salmonella enterica subsp. enterica serovar Enteritidis | |
| ERR387692 | Salmonella enterica subsp. enterica serovar Enteritidis | |
| ERR387693 | Salmonella enterica subsp. enterica serovar Enteritidis | |
| ERR387694 | Salmonella enterica subsp. enterica serovar Enteritidis | |
| ERR387695 | Salmonella enterica subsp. enterica serovar Enteritidis | |
| ERR387696 | Salmonella enterica subsp. enterica serovar Enteritidis | |
| ERR387723 | Salmonella enterica subsp. enterica serovar Enteritidis | |
| ERR410274 | Salmonella enterica subsp. enterica serovar Enteritidis | |
| ERR410275 | Salmonella enterica subsp. enterica serovar Enteritidis | |
| ERR420431 | Salmonella enterica subsp. enterica serovar Enteritidis | |
| ERR424894 | Salmonella enterica subsp. enterica serovar Enteritidis | |
| ERR424898 | Salmonella enterica subsp. enterica serovar Enteritidis | |
| ERR424900 | Salmonella enterica subsp. enterica serovar Enteritidis | |
| ERR424903 | Salmonella enterica subsp. enterica serovar Enteritidis | |
| ERR424904 | Salmonella enterica subsp. enterica serovar Enteritidis | |
| ERR424905 | Salmonella enterica subsp. enterica serovar Enteritidis | |
| ERR424906 | Salmonella enterica subsp. enterica serovar Enteritidis | |
| ERR424909 | Salmonella enterica subsp. enterica serovar Enteritidis | |
| ERR424912 | Salmonella enterica subsp. enterica serovar Enteritidis | |
| ERR424913 | Salmonella enterica subsp. enterica serovar Enteritidis | |
| ERR425332 | Salmonella enterica subsp. enterica serovar Enteritidis | |
| ERR425333 | Salmonella enterica subsp. enterica serovar Enteritidis | |
| ERR425334 | Salmonella enterica subsp. enterica serovar Enteritidis | |
| ERR425335 | Salmonella enterica subsp. enterica serovar Enteritidis | |
| ERR425336 | Salmonella enterica subsp. enterica serovar Enteritidis | |
| ERR425337 | Salmonella enterica subsp. enterica serovar Enteritidis | |
| ERR433221 | Salmonella enterica subsp. enterica serovar Enteritidis | |
| ERR433222 | Salmonella enterica subsp. enterica serovar Enteritidis | |
| ERR433223 | Salmonella enterica subsp. enterica serovar Enteritidis | |
| ERR433224 | Salmonella enterica subsp. enterica serovar Enteritidis | |
| ERR433225 | Salmonella enterica subsp. enterica serovar Enteritidis | |
| ERR433226 | Salmonella enterica subsp. enterica serovar Enteritidis | |
| ERR433227 | Salmonella enterica subsp. enterica serovar Enteritidis | |
| ERR433228 | Salmonella enterica subsp. enterica serovar Enteritidis | |
| ERR501633 | Salmonella enterica subsp. enterica serovar Enteritidis | |
| ERR501634 | Salmonella enterica subsp. enterica serovar Enteritidis | |
| ERR501635 | Salmonella enterica subsp. enterica serovar Enteritidis | |
| ERR501636 | Salmonella enterica subsp. enterica serovar Enteritidis | |
| ERR501637 | Salmonella enterica subsp. enterica serovar Enteritidis | |
| ERR582672 | Salmonella enterica subsp. enterica serovar Enteritidis | |
| ERR582673 | Salmonella enterica subsp. enterica serovar Enteritidis | |
| ERR582674 | Salmonella enterica subsp. enterica serovar Enteritidis | |
| ERR582675 | Salmonella enterica subsp. enterica serovar Enteritidis | |
| ERR582676 | Salmonella enterica subsp. enterica serovar Enteritidis | |
| ERR582677 | Salmonella enterica subsp. enterica serovar Enteritidis | |
| ERR582678 | Salmonella enterica subsp. enterica serovar Enteritidis | |
| ERR582679 | Salmonella enterica subsp. enterica serovar Enteritidis | |
| ERR638347 | Salmonella enterica subsp. enterica serovar Enteritidis | |
| ERR638348 | Salmonella enterica subsp. enterica serovar Enteritidis | |
| ERR638349 | Salmonella enterica subsp. enterica serovar Enteritidis | |
| ERR638350 | Salmonella enterica subsp. enterica serovar Enteritidis | |
| ERR638351 | Salmonella enterica subsp. enterica serovar Enteritidis | |
| ERR638352 | Salmonella enterica subsp. enterica serovar Enteritidis | |
| ERR638353 | Salmonella enterica subsp. enterica serovar Enteritidis | |
| ERR638354 | Salmonella enterica subsp. enterica serovar Enteritidis | |
| ERR638355 | Salmonella enterica subsp. enterica serovar Enteritidis | |
| ERR638356 | Salmonella enterica subsp. enterica serovar Enteritidis | |
| ERR638357 | Salmonella enterica subsp. enterica serovar Enteritidis | |
| ERR638358 | Salmonella enterica subsp. enterica serovar Enteritidis | |
| ERR638359 | Salmonella enterica subsp. enterica serovar Enteritidis | |
| ERR638360 | Salmonella enterica subsp. enterica serovar Enteritidis | |
| ERR638361 | Salmonella enterica subsp. enterica serovar Enteritidis | |
| ERR638362 | Salmonella enterica subsp. enterica serovar Enteritidis | |
| ERR653270 | Salmonella enterica subsp. enterica serovar Enteritidis | |
| ERR653271 | Salmonella enterica subsp. enterica serovar Enteritidis | |
| ERR653272 | Salmonella enterica subsp. enterica serovar Enteritidis | |
| ERR653273 | Salmonella enterica subsp. enterica serovar Enteritidis | |
| ERR653274 | Salmonella enterica subsp. enterica serovar Enteritidis | |
| ERR653275 | Salmonella enterica subsp. enterica serovar Enteritidis | |
| ERR653276 | Salmonella enterica subsp. enterica serovar Enteritidis | |
| ERR653277 | Salmonella enterica subsp. enterica serovar Enteritidis | |
| ERR653278 | Salmonella enterica subsp. enterica serovar Enteritidis | |
| ERR653279 | Salmonella enterica subsp. enterica serovar Enteritidis | |
| ERR653280 | Salmonella enterica subsp. enterica serovar Enteritidis | |
| ERR653281 | Salmonella enterica subsp. enterica serovar Enteritidis | |
| ERR653282 | Salmonella enterica subsp. enterica serovar Enteritidis | |
| ERR653283 | Salmonella enterica subsp. enterica serovar Enteritidis | |
| ERR653284 | Salmonella enterica subsp. enterica serovar Enteritidis | |
| ERR653285 | Salmonella enterica subsp. enterica serovar Enteritidis | |
| ERR653286 | Salmonella enterica subsp. enterica serovar Enteritidis | |
| ERR653287 | Salmonella enterica subsp. enterica serovar Enteritidis | |
| ERR653288 | Salmonella enterica subsp. enterica serovar Enteritidis | |
| ERR653289 | Salmonella enterica subsp. enterica serovar Enteritidis | |
| ERR653290 | Salmonella enterica subsp. enterica serovar Enteritidis | |
| ERR653291 | Salmonella enterica subsp. enterica serovar Enteritidis | |
| ERR653292 | Salmonella enterica subsp. enterica serovar Enteritidis | |
| ERR653293 | Salmonella enterica subsp. enterica serovar Enteritidis | |
| ERR653294 | Salmonella enterica subsp. enterica serovar Enteritidis | |
| ERR653295 | Salmonella enterica subsp. enterica serovar Enteritidis | |
| ERR653296 | Salmonella enterica subsp. enterica serovar Enteritidis | |
| ERR653297 | Salmonella enterica subsp. enterica serovar Enteritidis | |
| ERR653298 | Salmonella enterica subsp. enterica serovar Enteritidis | |
| ERR653299 | Salmonella enterica subsp. enterica serovar Enteritidis | |
| ERR653300 | Salmonella enterica subsp. enterica serovar Enteritidis | |
| ERR653301 | Salmonella enterica subsp. enterica serovar Enteritidis | |
| ERR653302 | Salmonella enterica subsp. enterica serovar Enteritidis | |
| ERR653303 | Salmonella enterica subsp. enterica serovar Enteritidis | |
| ERR653304 | Salmonella enterica subsp. enterica serovar Enteritidis | |
| ERR653305 | Salmonella enterica subsp. enterica serovar Enteritidis | |
| ERR653306 | Salmonella enterica subsp. enterica serovar Enteritidis | |
| ERR653307 | Salmonella enterica subsp. enterica serovar Enteritidis | |
| ERR653308 | Salmonella enterica subsp. enterica serovar Enteritidis | |
| ERR653309 | Salmonella enterica subsp. enterica serovar Enteritidis | |
| ERR653310 | Salmonella enterica subsp. enterica serovar Enteritidis | |
| ERR653315 | Salmonella enterica subsp. enterica serovar Enteritidis | |
| ERR653316 | Salmonella enterica subsp. enterica serovar Enteritidis | |
| ERR653317 | Salmonella enterica subsp. enterica serovar Enteritidis | |
| ERR653318 | Salmonella enterica subsp. enterica serovar Enteritidis | |
| ERR653319 | Salmonella enterica subsp. enterica serovar Enteritidis | |
| ERR653320 | Salmonella enterica subsp. enterica serovar Enteritidis | |
| ERR653321 | Salmonella enterica subsp. enterica serovar Enteritidis | |
| ERR653322 | Salmonella enterica subsp. enterica serovar Enteritidis | |
| ERR653323 | Salmonella enterica subsp. enterica serovar Enteritidis | |
| ERR653324 | Salmonella enterica subsp. enterica serovar Enteritidis | |
| ERR653325 | Salmonella enterica subsp. enterica serovar Enteritidis | |
| ERR653326 | Salmonella enterica subsp. enterica serovar Enteritidis | |
| ERR653327 | Salmonella enterica subsp. enterica serovar Enteritidis | |
| ERR653328 | Salmonella enterica subsp. enterica serovar Enteritidis | |
| ERR653329 | Salmonella enterica subsp. enterica serovar Enteritidis | |
| ERR653330 | Salmonella enterica subsp. enterica serovar Enteritidis | |
| ERR653331 | Salmonella enterica subsp. enterica serovar Enteritidis | |
| ERR653332 | Salmonella enterica subsp. enterica serovar Enteritidis | |
| ERR653333 | Salmonella enterica subsp. enterica serovar Enteritidis | |
| ERR653334 | Salmonella enterica subsp. enterica serovar Enteritidis | |
| ERR653983 | Salmonella enterica subsp. enterica serovar Enteritidis | |
| ERR653984 | Salmonella enterica subsp. enterica serovar Enteritidis | |
| ERR653985 | Salmonella enterica subsp. enterica serovar Enteritidis | |
| ERR653986 | Salmonella enterica subsp. enterica serovar Enteritidis | |
| ERR653987 | Salmonella enterica subsp. enterica serovar Enteritidis | |
| ERR653988 | Salmonella enterica subsp. enterica serovar Enteritidis | |
| ERR653989 | Salmonella enterica subsp. enterica serovar Enteritidis | |
| ERR653990 | Salmonella enterica subsp. enterica serovar Enteritidis | |
| ERR653991 | Salmonella enterica subsp. enterica serovar Enteritidis | |
| ERR653992 | Salmonella enterica subsp. enterica serovar Enteritidis | |
| ERR653993 | Salmonella enterica subsp. enterica serovar Enteritidis | |
| ERR653994 | Salmonella enterica subsp. enterica serovar Enteritidis | |
| ERR653995 | Salmonella enterica subsp. enterica serovar Enteritidis | |
| ERR653996 | Salmonella enterica subsp. enterica serovar Enteritidis | |
| ERR653997 | Salmonella enterica subsp. enterica serovar Enteritidis | |
| ERR653998 | Salmonella enterica subsp. enterica serovar Enteritidis | |
| ERR653999 | Salmonella enterica subsp. enterica serovar Enteritidis | |
| ERR654000 | Salmonella enterica subsp. enterica serovar Enteritidis | |
| ERR654001 | Salmonella enterica subsp. enterica serovar Enteritidis | |
| ERR654002 | Salmonella enterica subsp. enterica serovar Enteritidis | |
| ERR654003 | Salmonella enterica subsp. enterica serovar Enteritidis | |
| ERR654004 | Salmonella enterica subsp. enterica serovar Enteritidis | |
| ERR654005 | Salmonella enterica subsp. enterica serovar Enteritidis | |
| ERR654006 | Salmonella enterica subsp. enterica serovar Enteritidis | |
| ERR654489 | Salmonella enterica subsp. enterica serovar Enteritidis | |
| ERR654490 | Salmonella enterica subsp. enterica serovar Enteritidis | |
| ERR654491 | Salmonella enterica subsp. enterica serovar Enteritidis | |
| ERR654492 | Salmonella enterica subsp. enterica serovar Enteritidis | |
| ERR654493 | Salmonella enterica subsp. enterica serovar Enteritidis | |
| ERR654494 | Salmonella enterica subsp. enterica serovar Enteritidis | |
| ERR654495 | Salmonella enterica subsp. enterica serovar Enteritidis | |
| ERR654496 | Salmonella enterica subsp. enterica serovar Enteritidis | |
| ERR654502 | Salmonella enterica subsp. enterica serovar Enteritidis | |
| ERR654503 | Salmonella enterica subsp. enterica serovar Enteritidis | |
| ERR654504 | Salmonella enterica subsp. enterica serovar Enteritidis | |
| ERR654505 | Salmonella enterica subsp. enterica serovar Enteritidis | |
| ERR654506 | Salmonella enterica subsp. enterica serovar Enteritidis | |
| ERR654507 | Salmonella enterica subsp. enterica serovar Enteritidis | |
| ERR772449 | Salmonella enterica subsp. enterica serovar Enteritidis | |
| ERR776484 | Salmonella enterica subsp. enterica serovar Enteritidis | |
| ERR776485 | Salmonella enterica subsp. enterica serovar Enteritidis | |
| ERR778722 | Salmonella enterica subsp. enterica serovar Enteritidis str. LK5 | |
| ERR984704 | Salmonella enterica subsp. enterica serovar Enteritidis | |
| ERR984706 | Salmonella enterica subsp. enterica serovar Enteritidis | |
| ERR984719 | Salmonella enterica subsp. enterica serovar Enteritidis | |
| ERR984722 | Salmonella enterica subsp. enterica serovar Enteritidis | |
| ERR984732 | Salmonella enterica subsp. enterica serovar Enteritidis | |
| ERR984757 | Salmonella enterica subsp. enterica serovar Enteritidis | |
| ERR984764 | Salmonella enterica subsp. enterica serovar Enteritidis | |
| ERR984775 | Salmonella enterica subsp. enterica serovar Enteritidis | |
| ERR984785 | Salmonella enterica subsp. enterica serovar Enteritidis | |
| ERR984800 | Salmonella enterica subsp. enterica serovar Enteritidis | |
| ERR984803 | Salmonella enterica subsp. enterica serovar Enteritidis | |
| ERR984812 | Salmonella enterica subsp. enterica serovar Enteritidis | |
| ERR984822 | Salmonella enterica subsp. enterica serovar Enteritidis | |
| ERR984845 | Salmonella enterica subsp. enterica serovar Enteritidis | |
| ERR984871 | Salmonella enterica subsp. enterica serovar Enteritidis | |
| ERR984873 | Salmonella enterica subsp. enterica serovar Enteritidis | |
| ERR984884 | Salmonella enterica subsp. enterica serovar Enteritidis | |
| ERR984919 | Salmonella enterica subsp. enterica serovar Enteritidis | |
| ERR984964 | Salmonella enterica subsp. enterica serovar Enteritidis | |
| ERR998484 | Salmonella enterica subsp. enterica serovar Enteritidis | |
| ERR998502 | Salmonella enterica subsp. enterica serovar Enteritidis | |
| SRR1033744 | Salmonella enterica subsp. enterica serovar Enteritidis | |
| SRR1033745 | Salmonella enterica subsp. enterica serovar Enteritidis | |
| SRR1033746 | Salmonella enterica subsp. enterica serovar Enteritidis | |
| SRR1033747 | Salmonella enterica subsp. enterica serovar Enteritidis | |
| SRR1033748 | Salmonella enterica subsp. enterica serovar Enteritidis | |
| SRR1033749 | Salmonella enterica subsp. enterica serovar Enteritidis | |
| SRR1033750 | Salmonella enterica subsp. enterica serovar Enteritidis | |
| SRR1033751 | Salmonella enterica subsp. enterica serovar Enteritidis | |
| SRR1033752 | Salmonella enterica subsp. enterica serovar Enteritidis | |
| SRR1033753 | Salmonella enterica subsp. enterica serovar Enteritidis | |
| SRR1033754 | Salmonella enterica subsp. enterica serovar Enteritidis | |
| SRR1033755 | Salmonella enterica subsp. enterica serovar Enteritidis | |
| SRR1060547 | Salmonella enterica subsp. enterica serovar Enteritidis | |
| SRR1060653 | Salmonella enterica subsp. enterica serovar Enteritidis | |
| SRR1060654 | Salmonella enterica subsp. enterica serovar Enteritidis | |
| SRR1060706 | Salmonella enterica subsp. enterica serovar Enteritidis | |
| SRR1060743 | Salmonella enterica subsp. enterica serovar Enteritidis | |
| SRR1106360 | Salmonella enterica subsp. enterica serovar Enteritidis | |
| SRR1106373 | Salmonella enterica subsp. enterica serovar Enteritidis | |
| SRR1106383 | Salmonella enterica subsp. enterica serovar Enteritidis | |
| SRR1106494 | Salmonella enterica subsp. enterica serovar Enteritidis | |
| SRR1106495 | Salmonella enterica subsp. enterica serovar Enteritidis | |
| SRR1106496 | Salmonella enterica subsp. enterica serovar Enteritidis | |
| SRR1106497 | Salmonella enterica subsp. enterica serovar Enteritidis | |
| SRR1106498 | Salmonella enterica subsp. enterica serovar Enteritidis | |
| SRR1106499 | Salmonella enterica subsp. enterica serovar Enteritidis | |
| SRR1106500 | Salmonella enterica subsp. enterica serovar Enteritidis | |
| SRR1106501 | Salmonella enterica subsp. enterica serovar Enteritidis | |
| SRR1118722 | Salmonella enterica subsp. enterica serovar Enteritidis | |
| SRR1122490 | Salmonella enterica subsp. enterica serovar Enteritidis | |
| SRR1122491 | Salmonella enterica subsp. enterica serovar Enteritidis | |
| SRR1122492 | Salmonella enterica subsp. enterica serovar Enteritidis | |
| SRR1122659 | Salmonella enterica subsp. enterica serovar Enteritidis | |
| SRR1122665 | Salmonella enterica subsp. enterica serovar Enteritidis | |
| SRR1122704 | Salmonella enterica subsp. enterica serovar Enteritidis | |
| SRR1122738 | Salmonella enterica subsp. enterica serovar Enteritidis | |
| SRR1183736 | Salmonella enterica subsp. enterica serovar Enteritidis str. EC20110354 | |
| SRR1183737 | Salmonella enterica subsp. enterica serovar Enteritidis str. EC20110356 | |
| SRR1183738 | Salmonella enterica subsp. enterica serovar Enteritidis str. EC20110357 | |
| SRR1183739 | Salmonella enterica subsp. enterica serovar Enteritidis str. EC20110358 | |
| SRR1183740 | Salmonella enterica subsp. enterica serovar Enteritidis str. EC20110359 | |
| SRR1183741 | Salmonella enterica subsp. enterica serovar Enteritidis str. EC20110223 | |
| SRR1183742 | Salmonella enterica subsp. enterica serovar Enteritidis str. EC20110360 | |
| SRR1183743 | Salmonella enterica subsp. enterica serovar Enteritidis str. EC20110361 | |
| SRR1183744 | Salmonella enterica subsp. enterica serovar Enteritidis str. EC20111095 | |
| SRR1183745 | Salmonella enterica subsp. enterica serovar Enteritidis str. SA19992322 | |
| SRR1183746 | Salmonella enterica subsp. enterica serovar Enteritidis str. EC20110353 | |
| SRR1183747 | Salmonella enterica subsp. enterica serovar Enteritidis str. EC20110355 | |
| SRR1183748 | Salmonella enterica subsp. enterica serovar Enteritidis str. EC20090641 | |
| SRR1183749 | Salmonella enterica subsp. enterica serovar Enteritidis str. EC20090698 | |
| SRR1183750 | Salmonella enterica subsp. enterica serovar Enteritidis str. EC20110221 | |
| SRR1183751 | Salmonella enterica subsp. enterica serovar Enteritidis str. EC20110222 | |
| SRR1183752 | Salmonella enterica subsp. enterica serovar Enteritidis str. EC20111175 | |
| SRR1183753 | Salmonella enterica subsp. enterica serovar Enteritidis str. EC20111174 | |
| SRR1183754 | Salmonella enterica subsp. enterica serovar Enteritidis str. EC20100101 | |
| SRR1183755 | Salmonella enterica subsp. enterica serovar Enteritidis str. EC20100103 | |
| SRR1183756 | Salmonella enterica subsp. enterica serovar Enteritidis str. EC20090193 | |
| SRR1183757 | Salmonella enterica subsp. enterica serovar Enteritidis str. EC20090135 | |
| SRR1183758 | Salmonella enterica subsp. enterica serovar Enteritidis str. EC20090332 | |
| SRR1183759 | Salmonella enterica subsp. enterica serovar Enteritidis str. EC20090531 | |
| SRR1183760 | Salmonella enterica subsp. enterica serovar Enteritidis str. EC20090884 | |
| SRR1183761 | Salmonella enterica subsp. enterica serovar Enteritidis str. EC20100130 | |
| SRR1183762 | Salmonella enterica subsp. enterica serovar Enteritidis str. EC20100134 | |
| SRR1183763 | Salmonella enterica subsp. enterica serovar Enteritidis str. EC20120002 | |
| SRR1183764 | Salmonella enterica subsp. enterica serovar Enteritidis str. EC20120003 | |
| SRR1183765 | Salmonella enterica subsp. enterica serovar Enteritidis str. EC20120005 | |
| SRR1183766 | Salmonella enterica subsp. enterica serovar Enteritidis str. EC20120007 | |
| SRR1183767 | Salmonella enterica subsp. enterica serovar Enteritidis str. EC20120008 | |
| SRR1183768 | Salmonella enterica subsp. enterica serovar Enteritidis str. EC20120009 | |
| SRR1183769 | Salmonella enterica subsp. enterica serovar Enteritidis str. SA19980677 | |
| SRR1183770 | Salmonella enterica subsp. enterica serovar Enteritidis str. SA19970510 | |
| SRR1183771 | Salmonella enterica subsp. enterica serovar Enteritidis str. SA19970769 | |
| SRR1183772 | Salmonella enterica subsp. enterica serovar Enteritidis str. SA20093266 | |
| SRR1183773 | Salmonella enterica subsp. enterica serovar Enteritidis str. SA20094682 | |
| SRR1183774 | Salmonella enterica subsp. enterica serovar Enteritidis str. SA20084384 | |
| SRR1183775 | Salmonella enterica subsp. enterica serovar Enteritidis str. SA20084824 | |
| SRR1183776 | Salmonella enterica subsp. enterica serovar Enteritidis str. SA20094177 | |
| SRR1183777 | Salmonella enterica subsp. enterica serovar Enteritidis str. SA20084644 | |
| SRR1183778 | Salmonella enterica subsp. enterica serovar Enteritidis str. SA20100239 | |
| SRR1183779 | Salmonella enterica subsp. enterica serovar Enteritidis str. SA20100349 | |
| SRR1183780 | Salmonella enterica subsp. enterica serovar Enteritidis str. SA20121703 | |
| SRR1183781 | Salmonella enterica subsp. enterica serovar Enteritidis str. SA20094521 | |
| SRR1183782 | Salmonella enterica subsp. enterica serovar Enteritidis str. SA19981522 | |
| SRR1183783 | Salmonella enterica subsp. enterica serovar Enteritidis str. SA19981857 | |
| SRR1183784 | Salmonella enterica subsp. enterica serovar Enteritidis str. SA19960848 | |
| SRR1183785 | Salmonella enterica subsp. enterica serovar Enteritidis str. SA20082034 | |
| SRR1183786 | Salmonella enterica subsp. enterica serovar Enteritidis str. SA20085285 | |
| SRR1183787 | Salmonella enterica subsp. enterica serovar Enteritidis str. SA20083636 | |
| SRR1183788 | Salmonella enterica subsp. enterica serovar Enteritidis str. SA20094352 | |
| SRR1183789 | Salmonella enterica subsp. enterica serovar Enteritidis str. SA20095440 | |
| SRR1183790 | Salmonella enterica subsp. enterica serovar Enteritidis str. SA19942384 | |
| SRR1183791 | Salmonella enterica subsp. enterica serovar Enteritidis str. SA19943269 | |
| SRR1183792 | Salmonella enterica subsp. enterica serovar Enteritidis str. SA20123395 | |
| SRR1183793 | Salmonella enterica subsp. enterica serovar Enteritidis str. SA19971331 | |
| SRR1183794 | Salmonella enterica subsp. enterica serovar Enteritidis str. SA19961622 | |
| SRR1183795 | Salmonella enterica subsp. enterica serovar Enteritidis str. SA19983126 | |
| SRR1183796 | Salmonella enterica subsp. enterica serovar Enteritidis str. SA19994216 | |
| SRR1183797 | Salmonella enterica subsp. enterica serovar Enteritidis str. SA19930684 | |
| SRR1183799 | Salmonella enterica subsp. enterica serovar Enteritidis str. SA19982831 | |
| SRR1183800 | Salmonella enterica subsp. enterica serovar Enteritidis str. SA20094803 | |
| SRR1183801 | Salmonella enterica subsp. enterica serovar Enteritidis str. SA20094350 | |
| SRR1183802 | Salmonella enterica subsp. enterica serovar Enteritidis str. SA20094301 | |
| SRR1183803 | Salmonella enterica subsp. enterica serovar Enteritidis str. SA20083456 | |
| SRR1183804 | Salmonella enterica subsp. enterica serovar Enteritidis str. SA20095309 | |
| SRR1183805 | Salmonella enterica subsp. enterica serovar Enteritidis str. SA20092320 | |
| SRR1183806 | Salmonella enterica subsp. enterica serovar Enteritidis str. SA20093784 | |
| SRR1183807 | Salmonella enterica subsp. enterica serovar Enteritidis str. SA20093977 | |
| SRR1183808 | Salmonella enterica subsp. enterica serovar Enteritidis str. SA20093788 | |
| SRR1183809 | Salmonella enterica subsp. enterica serovar Enteritidis str. SA20093430 | |
| SRR1183810 | Salmonella enterica subsp. enterica serovar Enteritidis str. SA20090435 | |
| SRR1183811 | Salmonella enterica subsp. enterica serovar Enteritidis str. SA20093421 | |
| SRR1183812 | Salmonella enterica subsp. enterica serovar Enteritidis str. SA20094389 | |
| SRR1183813 | Salmonella enterica subsp. enterica serovar Enteritidis str. SA20094383 | |
| SRR1183814 | Salmonella enterica subsp. enterica serovar Enteritidis str. SA20090419 | |
| SRR1183815 | Salmonella enterica subsp. enterica serovar Enteritidis str. SA20094642 | |
| SRR1183816 | Salmonella enterica subsp. enterica serovar Enteritidis str. SA20093950 | |
| SRR1183817 | Salmonella enterica subsp. enterica serovar Enteritidis str. SA20093543 | |
| SRR1183818 | Salmonella enterica subsp. enterica serovar Enteritidis str. SA20090877 | |
| SRR1183819 | Salmonella enterica subsp. enterica serovar Enteritidis str. SA20093538 | |
| SRR1183820 | Salmonella enterica subsp. enterica serovar Enteritidis str. SA20091739 | |
| SRR1183821 | Salmonella enterica subsp. enterica serovar Enteritidis str. SA20094079 | |
| SRR1183822 | Salmonella enterica subsp. enterica serovar Enteritidis str. EC20121541 | |
| SRR1183823 | Salmonella enterica subsp. enterica serovar Enteritidis str. EC20121825 | |
| SRR1183824 | Salmonella enterica subsp. enterica serovar Enteritidis str. EC20121826 | |
| SRR1183825 | Salmonella enterica subsp. enterica serovar Enteritidis str. EC20120213 | |
| SRR1183826 | Salmonella enterica subsp. enterica serovar Enteritidis str. EC20120200 | |
| SRR1183827 | Salmonella enterica subsp. enterica serovar Enteritidis str. EC20121004 | |
| SRR1183828 | Salmonella enterica subsp. enterica serovar Enteritidis str. EC20120963 | |
| SRR1183829 | Salmonella enterica subsp. enterica serovar Enteritidis str. EC20120776 | |
| SRR1183830 | Salmonella enterica subsp. enterica serovar Enteritidis str. EC20120775 | |
| SRR1183831 | Salmonella enterica subsp. enterica serovar Enteritidis str. EC20120685 | |
| SRR1183832 | Salmonella enterica subsp. enterica serovar Enteritidis str. EC20111576 | |
| SRR1183833 | Salmonella enterica subsp. enterica serovar Enteritidis str. EC20120544 | |
| SRR1183834 | Salmonella enterica subsp. enterica serovar Enteritidis str. EC20120528 | |
| SRR1183835 | Salmonella enterica subsp. enterica serovar Enteritidis str. EC20111515 | |
| SRR1183836 | Salmonella enterica subsp. enterica serovar Enteritidis str. EC20111514 | |
| SRR1183837 | Salmonella enterica subsp. enterica serovar Enteritidis str. EC20121751 | |
| SRR1183838 | Salmonella enterica subsp. enterica serovar Enteritidis str. EC20121750 | |
| SRR1183839 | Salmonella enterica subsp. enterica serovar Enteritidis str. EC20120970 | |
| SRR1183840 | Salmonella enterica subsp. enterica serovar Enteritidis str. EC20120548 | |
| SRR1183841 | Salmonella enterica subsp. enterica serovar Enteritidis str. EC20120505 | |
| SRR1183842 | Salmonella enterica subsp. enterica serovar Enteritidis str. EC20120498 | |
| SRR1183843 | Salmonella enterica subsp. enterica serovar Enteritidis str. EC20120240 | |
| SRR1183844 | Salmonella enterica subsp. enterica serovar Enteritidis str. EC20120229 | |
| SRR1183845 | Salmonella enterica subsp. enterica serovar Enteritidis str. EC20120219 | |
| SRR1183846 | Salmonella enterica subsp. enterica serovar Enteritidis str. EC20111510 | |
| SRR1183847 | Salmonella enterica subsp. enterica serovar Enteritidis str. EC20120722 | |
| SRR1183848 | Salmonella enterica subsp. enterica serovar Enteritidis str. EC20121753 | |
| SRR1183849 | Salmonella enterica subsp. enterica serovar Enteritidis str. EC20120469 | |
| SRR1183850 | Salmonella enterica subsp. enterica serovar Enteritidis str. EC20120697 | |
| SRR1183851 | Salmonella enterica subsp. enterica serovar Enteritidis str. EC20121744 | |
| SRR1183852 | Salmonella enterica subsp. enterica serovar Enteritidis str. EC20121765 | |
| SRR1183853 | Salmonella enterica subsp. enterica serovar Enteritidis str. EC20120496 | |
| SRR1183854 | Salmonella enterica subsp. enterica serovar Enteritidis str. EC20121542 | |
| SRR1183855 | Salmonella enterica subsp. enterica serovar Enteritidis str. EC20120968 | |
| SRR1183856 | Salmonella enterica subsp. enterica serovar Enteritidis str. EC20120677 | |
| SRR1183857 | Salmonella enterica subsp. enterica serovar Enteritidis str. EC20121748 | |
| SRR1183858 | Salmonella enterica subsp. enterica serovar Enteritidis str. EC20121672 | |
| SRR1183859 | Salmonella enterica subsp. enterica serovar Enteritidis str. EC20121747 | |
| SRR1183860 | Salmonella enterica subsp. enterica serovar Enteritidis str. EC20121746 | |
| SRR1183861 | Salmonella enterica subsp. enterica serovar Enteritidis str. EC20120765 | |
| SRR1183862 | Salmonella enterica subsp. enterica serovar Enteritidis str. EC20121671 | |
| SRR1183863 | Salmonella enterica subsp. enterica serovar Enteritidis str. EC20120555 | |
| SRR1183864 | Salmonella enterica subsp. enterica serovar Enteritidis str. EC20111554 | |
| SRR1183865 | Salmonella enterica subsp. enterica serovar Enteritidis str. EC20120497 | |
| SRR1183866 | Salmonella enterica subsp. enterica serovar Enteritidis str. EC20120994 | |
| SRR1183867 | Salmonella enterica subsp. enterica serovar Enteritidis str. EC20120916 | |
| SRR1183868 | Salmonella enterica subsp. enterica serovar Enteritidis str. EC20121812 | |
| SRR1183869 | Salmonella enterica subsp. enterica serovar Enteritidis str. EC20111561 | |
| SRR1183870 | Salmonella enterica subsp. enterica serovar Enteritidis str. EC20122045 | |
| SRR1183871 | Salmonella enterica subsp. enterica serovar Enteritidis str. EC20122033 | |
| SRR1183872 | Salmonella enterica subsp. enterica serovar Enteritidis str. EC20122031 | |
| SRR1183873 | Salmonella enterica subsp. enterica serovar Enteritidis str. EC20122026 | |
| SRR1183874 | Salmonella enterica subsp. enterica serovar Enteritidis str. EC20122022 | |
| SRR1183875 | Salmonella enterica subsp. enterica serovar Enteritidis str. EC20121990 | |
| SRR1183876 | Salmonella enterica subsp. enterica serovar Enteritidis str. EC20121989 | |
| SRR1183877 | Salmonella enterica subsp. enterica serovar Enteritidis str. EC20121986 | |
| SRR1183878 | Salmonella enterica subsp. enterica serovar Enteritidis str. EC20121976 | |
| SRR1183879 | Salmonella enterica subsp. enterica serovar Enteritidis str. EC20121970 | |
| SRR1183880 | Salmonella enterica subsp. enterica serovar Enteritidis str. EC20121969 | |
| SRR1183881 | Salmonella enterica subsp. enterica serovar Enteritidis str. EC20100131 | |
| SRR1183882 | Salmonella enterica subsp. enterica serovar Enteritidis str. EC20090530 | |
| SRR1183883 | Salmonella enterica subsp. enterica serovar Enteritidis str. EC20100100 | |
| SRR1183884 | Salmonella enterica subsp. enterica serovar Enteritidis str. EC20090195 | |
| SRR1183885 | Salmonella enterica subsp. enterica serovar Enteritidis str. EC20130345 | |
| SRR1183886 | Salmonella enterica subsp. enterica serovar Enteritidis str. EC20130346 | |
| SRR1183887 | Salmonella enterica subsp. enterica serovar Enteritidis str. EC20130347 | |
| SRR1183888 | Salmonella enterica subsp. enterica serovar Enteritidis str. EC20130348 | |
| SRR1183889 | Salmonella enterica subsp. enterica serovar Enteritidis str. EC20121175 | |
| SRR1183890 | Salmonella enterica subsp. enterica serovar Enteritidis str. EC20121176 | |
| SRR1183891 | Salmonella enterica subsp. enterica serovar Enteritidis str. EC20121177 | |
| SRR1183892 | Salmonella enterica subsp. enterica serovar Enteritidis str. EC20121178 | |
| SRR1183893 | Salmonella enterica subsp. enterica serovar Enteritidis str. EC20121179 | |
| SRR1183894 | Salmonella enterica subsp. enterica serovar Enteritidis str. EC20121180 | |
| SRR1183895 | Salmonella enterica subsp. enterica serovar Enteritidis str. EC20100088 | |
| SRR1183896 | Salmonella enterica subsp. enterica serovar Enteritidis str. EC20100089 | |
| SRR1183897 | Salmonella enterica subsp. enterica serovar Enteritidis str. EC20100325 | |
| SRR1183898 | Salmonella enterica subsp. enterica serovar Enteritidis str. EC20120051 | |
| SRR1183899 | Salmonella enterica subsp. enterica serovar Enteritidis str. EC20120356 | |
| SRR1183900 | Salmonella enterica subsp. enterica serovar Enteritidis str. EC20120580 | |
| SRR1183901 | Salmonella enterica subsp. enterica serovar Enteritidis str. EC20120581 | |
| SRR1183902 | Salmonella enterica subsp. enterica serovar Enteritidis str. EC20120590 | |
| SRR1183903 | Salmonella enterica subsp. enterica serovar Enteritidis str. EC20120597 | |
| SRR1183904 | Salmonella enterica subsp. enterica serovar Enteritidis str. EC20120686 | |
| SRR1183905 | Salmonella enterica subsp. enterica serovar Enteritidis str. EC20120687 | |
| SRR1183906 | Salmonella enterica subsp. enterica serovar Enteritidis str. EC20120734 | |
| SRR1183907 | Salmonella enterica subsp. enterica serovar Enteritidis str. EC20120738 | |
| SRR1183908 | Salmonella enterica subsp. enterica serovar Enteritidis str. EC20120773 | |
| SRR1183909 | Salmonella enterica subsp. enterica serovar Enteritidis str. EC20120774 | |
| SRR1183910 | Salmonella enterica subsp. enterica serovar Enteritidis str. EC20120917 | |
| SRR1183911 | Salmonella enterica subsp. enterica serovar Enteritidis str. EC20120918 | |
| SRR1183912 | Salmonella enterica subsp. enterica serovar Enteritidis str. EC20120925 | |
| SRR1183913 | Salmonella enterica subsp. enterica serovar Enteritidis str. EC20121689 | |
| SRR1183914 | Salmonella enterica subsp. enterica serovar Enteritidis str. EC20120927 | |
| SRR1183915 | Salmonella enterica subsp. enterica serovar Enteritidis str. EC20120929 | |
| SRR1183916 | Salmonella enterica subsp. enterica serovar Enteritidis str. EC20120969 | |
| SRR1183970 | Salmonella enterica subsp. enterica serovar Enteritidis str. EC20110354 | |
| SRR1183971 | Salmonella enterica subsp. enterica serovar Enteritidis str. EC20110356 | |
| SRR1183972 | Salmonella enterica subsp. enterica serovar Enteritidis str. EC20110357 | |
| SRR1183973 | Salmonella enterica subsp. enterica serovar Enteritidis str. EC20110358 | |
| SRR1183974 | Salmonella enterica subsp. enterica serovar Enteritidis str. EC20110359 | |
| SRR1183975 | Salmonella enterica subsp. enterica serovar Enteritidis str. EC20110223 | |
| SRR1183976 | Salmonella enterica subsp. enterica serovar Enteritidis str. EC20110360 | |
| SRR1183977 | Salmonella enterica subsp. enterica serovar Enteritidis str. EC20110361 | |
| SRR1183978 | Salmonella enterica subsp. enterica serovar Enteritidis str. EC20111095 | |
| SRR1183979 | Salmonella enterica subsp. enterica serovar Enteritidis str. EC20110353 | |
| SRR1183980 | Salmonella enterica subsp. enterica serovar Enteritidis str. EC20110355 | |
| SRR1183981 | Salmonella enterica subsp. enterica serovar Enteritidis str. EC20090641 | |
| SRR1183982 | Salmonella enterica subsp. enterica serovar Enteritidis str. EC20090698 | |
| SRR1183983 | Salmonella enterica subsp. enterica serovar Enteritidis str. EC20110221 | |
| SRR1183984 | Salmonella enterica subsp. enterica serovar Enteritidis str. EC20111175 | |
| SRR1183985 | Salmonella enterica subsp. enterica serovar Enteritidis str. EC20111174 | |
| SRR1183986 | Salmonella enterica subsp. enterica serovar Enteritidis str. EC20100101 | |
| SRR1183987 | Salmonella enterica subsp. enterica serovar Enteritidis str. EC20100103 | |
| SRR1183988 | Salmonella enterica subsp. enterica serovar Enteritidis str. EC20090193 | |
| SRR1183989 | Salmonella enterica subsp. enterica serovar Enteritidis str. EC20090135 | |
| SRR1183990 | Salmonella enterica subsp. enterica serovar Enteritidis str. EC20090332 | |
| SRR1183991 | Salmonella enterica subsp. enterica serovar Enteritidis str. EC20090531 | |
| SRR1183992 | Salmonella enterica subsp. enterica serovar Enteritidis str. EC20090884 | |
| SRR1183993 | Salmonella enterica subsp. enterica serovar Enteritidis str. EC20100130 | |
| SRR1183994 | Salmonella enterica subsp. enterica serovar Enteritidis str. EC20100134 | |
| SRR1183995 | Salmonella enterica subsp. enterica serovar Enteritidis str. EC20120002 | |
| SRR1183996 | Salmonella enterica subsp. enterica serovar Enteritidis str. EC20120003 | |
| SRR1183997 | Salmonella enterica subsp. enterica serovar Enteritidis str. EC20120005 | |
| SRR1183998 | Salmonella enterica subsp. enterica serovar Enteritidis str. EC20120007 | |
| SRR1183999 | Salmonella enterica subsp. enterica serovar Enteritidis str. EC20120008 | |
| SRR1184000 | Salmonella enterica subsp. enterica serovar Enteritidis str. EC20120009 | |
| SRR1206205 | Salmonella enterica subsp. enterica serovar Enteritidis str. CFSAN004342 | |
| SRR1532569 | Salmonella enterica subsp. enterica serovar Enteritidis str. 08-1080 | |
| SRR1532570 | Salmonella enterica subsp. enterica serovar Enteritidis str. 2009K0958 | |
| SRR1532571 | Salmonella enterica subsp. enterica serovar Enteritidis str. 2009K1651 | |
| SRR1532572 | Salmonella enterica subsp. enterica serovar Enteritidis str. 2010K-0271 | |
| SRR1532573 | Salmonella enterica subsp. enterica serovar Enteritidis str. 2010K-0297 | |
| SRR1532574 | Salmonella enterica subsp. enterica serovar Enteritidis str. 2010K-0300 | |
| SRR1532575 | Salmonella enterica subsp. enterica serovar Enteritidis str. 2010K-0302 | |
| SRR1532576 | Salmonella enterica subsp. enterica serovar Enteritidis str. 2010K-0313 | |
| SRR1532577 | Salmonella enterica subsp. enterica serovar Enteritidis str. 2010K-0329 | |
| SRR1532578 | Salmonella enterica subsp. enterica serovar Enteritidis str. 2010K-1923 | |
| SRR1532579 | Salmonella enterica subsp. enterica serovar Enteritidis str. 2010K-0286 | |
| SRR1532580 | Salmonella enterica subsp. enterica serovar Enteritidis str. 2010K-0287 | |
| SRR1532581 | Salmonella enterica subsp. enterica serovar Enteritidis str. 2010K-0302 | |
| SRR1532583 | Salmonella enterica subsp. enterica serovar Enteritidis str. 2009K1726 | |
| SRR1532706 | Salmonella enterica subsp. enterica serovar Enteritidis str. 2010K-0262 | |
| SRR1532707 | Salmonella enterica subsp. enterica serovar Enteritidis str. 2010K-2029 | |
| SRR1532708 | Salmonella enterica subsp. enterica serovar Enteritidis str. 54-2220 | |
| SRR1532709 | Salmonella enterica subsp. enterica serovar Enteritidis str. 54-2220 | |
| SRR1532710 | Salmonella enterica subsp. enterica serovar Enteritidis str. 75-2732 | |
| SRR1532711 | Salmonella enterica subsp. enterica serovar Enteritidis str. 75-2732 | |
| SRR1532712 | Salmonella enterica subsp. enterica serovar Enteritidis str. 84-1226 | |
| SRR1532713 | Salmonella enterica subsp. enterica serovar Enteritidis str. 93-0063 | |
| SRR1532714 | Salmonella enterica subsp. enterica serovar Enteritidis str. 04-0307 | |
| SRR1532715 | Salmonella enterica subsp. enterica serovar Enteritidis str. 2009K0477 | |
| SRR1532716 | Salmonella enterica subsp. enterica serovar Enteritidis str. 2009K0477 | |
| SRR1532717 | Salmonella enterica subsp. enterica serovar Enteritidis str. 2009K0479 | |
| SRR1532718 | Salmonella enterica subsp. enterica serovar Enteritidis str. 2009K1324 | |
| SRR1532719 | Salmonella enterica subsp. enterica serovar Enteritidis str. 2010K-0263 | |
| SRR1532721 | Salmonella enterica subsp. enterica serovar Enteritidis str. 2010K-0264 | |
| SRR1532722 | Salmonella enterica subsp. enterica serovar Enteritidis str. 2010K-0264 | |
| SRR1532723 | Salmonella enterica subsp. enterica serovar Enteritidis str. 2010K-0268 | |
| SRR1532724 | Salmonella enterica subsp. enterica serovar Enteritidis str. 2010K-0277 | |
| SRR1532725 | Salmonella enterica subsp. enterica serovar Enteritidis str. 2010K-0351 | |
| SRR1532726 | Salmonella enterica subsp. enterica serovar Enteritidis str. 08-1080 | |
| SRR1532727 | Salmonella enterica subsp. enterica serovar Enteritidis str. 2010K-0267 | |
| SRR1532728 | Salmonella enterica subsp. enterica serovar Enteritidis str. 2010K-0284 | |
| SRR1532729 | Salmonella enterica subsp. enterica serovar Enteritidis str. 2010K-0286 | |
| SRR1532730 | Salmonella enterica subsp. enterica serovar Enteritidis str. 2010K-0303 | |
| SRR1532731 | Salmonella enterica subsp. enterica serovar Enteritidis str. 93-0063 | |
| SRR1532732 | Salmonella enterica subsp. enterica serovar Enteritidis str. 98-0467 | |
| SRR1532733 | Salmonella enterica subsp. enterica serovar Enteritidis str. 2009K1324 | |
| SRR1532734 | Salmonella enterica subsp. enterica serovar Enteritidis str. 2010K-0301 | |
| SRR1532735 | Salmonella enterica subsp. enterica serovar Enteritidis str. 2010K-0301 | |
| SRR1532737 | Salmonella enterica subsp. enterica serovar Enteritidis str. 2010K-0345 | |
| SRR1533150 | Salmonella enterica subsp. enterica serovar Enteritidis | |
| SRR1533454 | Salmonella enterica subsp. enterica serovar Enteritidis | |
| SRR1533455 | Salmonella enterica subsp. enterica serovar Enteritidis | |
| SRR1533456 | Salmonella enterica subsp. enterica serovar Enteritidis | |
| SRR1533457 | Salmonella enterica subsp. enterica serovar Enteritidis | |
| SRR1533592 | Salmonella enterica subsp. enterica serovar Enteritidis | |
| SRR1533624 | Salmonella enterica subsp. enterica serovar Enteritidis | |
| SRR1533832 | Salmonella enterica subsp. enterica serovar Enteritidis | |
| SRR1533833 | Salmonella enterica subsp. enterica serovar Enteritidis | |
| SRR1533834 | Salmonella enterica subsp. enterica serovar Enteritidis | |
| SRR1533835 | Salmonella enterica subsp. enterica serovar Enteritidis | |
| SRR1536806 | Salmonella enterica subsp. enterica serovar Enteritidis str. 2010K-1832 | |
| SRR1536807 | Salmonella enterica subsp. enterica serovar Enteritidis str. 77-0915 | |
| SRR1536808 | Salmonella enterica subsp. enterica serovar Enteritidis str. UC10 | |
| SRR1536809 | Salmonella enterica subsp. enterica serovar Enteritidis str. J0903 | |
| SRR1536812 | Salmonella enterica subsp. enterica serovar Enteritidis str. 82631 | |
| SRR1536813 | Salmonella enterica subsp. enterica serovar Enteritidis str. 81748 | |
| SRR1536814 | Salmonella enterica subsp. enterica serovar Enteritidis str. 77320 | |
| SRR1536815 | Salmonella enterica subsp. enterica serovar Enteritidis str. 81748 | |
| SRR1536817 | Salmonella enterica subsp. enterica serovar Enteritidis str. UC07 | |
| SRR1536818 | Salmonella enterica subsp. enterica serovar Enteritidis str. UC07 | |
| SRR1536819 | Salmonella enterica subsp. enterica serovar Enteritidis str. 07-0056 | |
| SRR1536821 | Salmonella enterica subsp. enterica serovar Enteritidis str. 77320 | |
| SRR1536824 | Salmonella enterica subsp. enterica serovar Enteritidis str. 77-0915 | |
| SRR1536826 | Salmonella enterica subsp. enterica serovar Enteritidis str. S-380 | |
| SRR1536827 | Salmonella enterica subsp. enterica serovar Enteritidis str. 55795 | |
| SRR1536828 | Salmonella enterica subsp. enterica serovar Enteritidis str. 78296 | |
| SRR1536829 | Salmonella enterica subsp. enterica serovar Enteritidis str. 97569 | |
| SRR1536830 | Salmonella enterica subsp. enterica serovar Enteritidis str. 93-7922A | |
| SRR1536831 | Salmonella enterica subsp. enterica serovar Enteritidis str. 9810102B | |
| SRR1536832 | Salmonella enterica subsp. enterica serovar Enteritidis str. 76-0331 | |
| SRR1536834 | Salmonella enterica subsp. enterica serovar Enteritidis str. 9810102B | |
| SRR1536837 | Salmonella enterica subsp. enterica serovar Enteritidis str. 0804789B | |
| SRR1544518 | Salmonella enterica subsp. enterica serovar Enteritidis str. UC03 | |
| SRR1544519 | Salmonella enterica subsp. enterica serovar Enteritidis str. UC10 | |
| SRR1544520 | Salmonella enterica subsp. enterica serovar Enteritidis str. UC02 | |
| SRR1544535 | Salmonella enterica subsp. enterica serovar Enteritidis str. 85366 | |
| SRR1544536 | Salmonella enterica subsp. enterica serovar Enteritidis str. 61979 | |
| SRR1544537 | Salmonella enterica subsp. enterica serovar Enteritidis str. 97569 | |
| SRR1544538 | Salmonella enterica subsp. enterica serovar Enteritidis str. 93-2836A | |
| SRR1544539 | Salmonella enterica subsp. enterica serovar Enteritidis str. 0804789B | |
| SRR1544540 | Salmonella enterica subsp. enterica serovar Enteritidis str. UC03 | |
| SRR1544541 | Salmonella enterica subsp. enterica serovar Enteritidis str. UC11 | |
| SRR1544542 | Salmonella enterica subsp. enterica serovar Enteritidis str. UC13 | |
| SRR1544543 | Salmonella enterica subsp. enterica serovar Enteritidis str. UC13 | |
| SRR1544544 | Salmonella enterica subsp. enterica serovar Enteritidis str. 39997 | |
| SRR1544545 | Salmonella enterica subsp. enterica serovar Enteritidis str. 2010K-1028 | |
| SRR1544546 | Salmonella enterica subsp. enterica serovar Enteritidis str. 2010K-1369 | |
| SRR1544547 | Salmonella enterica subsp. enterica serovar Enteritidis str. 2010K-1554 | |
| SRR1544548 | Salmonella enterica subsp. enterica serovar Enteritidis str. 76-0331 | |
| SRR1544549 | Salmonella enterica subsp. enterica serovar Enteritidis str. J0828 | |
| SRR1544550 | Salmonella enterica subsp. enterica serovar Enteritidis str. S-277 | |
| SRR1544551 | Salmonella enterica subsp. enterica serovar Enteritidis str. 61080 | |
| SRR1544552 | Salmonella enterica subsp. enterica serovar Enteritidis str. 93-6175B | |
| SRR1544553 | Salmonella enterica subsp. enterica serovar Enteritidis str. 93-7741 | |
| SRR1544554 | Salmonella enterica subsp. enterica serovar Enteritidis str. 02-2966 | |
| SRR1544555 | Salmonella enterica subsp. enterica serovar Enteritidis str. 0502571 | |
| SRR1544556 | Salmonella enterica subsp. enterica serovar Enteritidis str. 0701376-4 | |
| SRR1544557 | Salmonella enterica subsp. enterica serovar Enteritidis str. 0811210F | |
| SRR1544558 | Salmonella enterica subsp. enterica serovar Enteritidis str. UC02 | |
| SRR1544559 | Salmonella enterica subsp. enterica serovar Enteritidis str. UC12 | |
| SRR1544560 | Salmonella enterica subsp. enterica serovar Enteritidis str. UC12 | |
| SRR1544561 | Salmonella enterica subsp. enterica serovar Enteritidis str. J0915 | |
| SRR1544564 | Salmonella enterica subsp. enterica serovar Enteritidis str. 07-0056 | |
| SRR1544565 | Salmonella enterica subsp. enterica serovar Enteritidis str. 98961 | |
| SRR1544566 | Salmonella enterica subsp. enterica serovar Enteritidis str. 93-7922A | |
| SRR1544567 | Salmonella enterica subsp. enterica serovar Enteritidis str. 98-9534 | |
| SRR1544568 | Salmonella enterica subsp. enterica serovar Enteritidis str. 98-9534 | |
| SRR1544569 | Salmonella enterica subsp. enterica serovar Enteritidis str. 2010K-1028 | |
| SRR1544570 | Salmonella enterica subsp. enterica serovar Enteritidis str. 2010K-1554 | |
| SRR1544571 | Salmonella enterica subsp. enterica serovar Enteritidis str. J0903 | |
| SRR1544572 | Salmonella enterica subsp. enterica serovar Enteritidis str. 34986 | |
| SRR1544573 | Salmonella enterica subsp. enterica serovar Enteritidis str. 93-6175B | |
| SRR1544574 | Salmonella enterica subsp. enterica serovar Enteritidis str. 93-7741 | |
| SRR1544575 | Salmonella enterica subsp. enterica serovar Enteritidis str. 02-2966 | |
| SRR1544576 | Salmonella enterica subsp. enterica serovar Enteritidis str. 0502571 | |
| SRR1544577 | Salmonella enterica subsp. enterica serovar Enteritidis str. 0811210F | |
| SRR1544578 | Salmonella enterica subsp. enterica serovar Enteritidis str. 1102933A | |
| SRR1544579 | Salmonella enterica subsp. enterica serovar Enteritidis str. UC11 | |
| SRR1544581 | Salmonella enterica subsp. enterica serovar Enteritidis str. 93-2836A | |
| SRR1544582 | Salmonella enterica subsp. enterica serovar Enteritidis str. 1102933A | |
| SRR1544583 | Salmonella enterica subsp. enterica serovar Enteritidis str. 0701376-4 | |
| SRR1633495 | Salmonella enterica subsp. enterica serovar Enteritidis | |
| SRR1635060 | Salmonella enterica subsp. enterica serovar Enteritidis | |
| SRR1635065 | Salmonella enterica subsp. enterica serovar Enteritidis | |
| SRR1635066 | Salmonella enterica subsp. enterica serovar Enteritidis | |
| SRR1635072 | Salmonella enterica subsp. enterica serovar Enteritidis | |
| SRR1635087 | Salmonella enterica subsp. enterica serovar Enteritidis | |
| SRR1635092 | Salmonella enterica subsp. enterica serovar Enteritidis | |
| SRR1635097 | Salmonella enterica subsp. enterica serovar Enteritidis | |
| SRR1635101 | Salmonella enterica subsp. enterica serovar Enteritidis | |
| SRR1635103 | Salmonella enterica subsp. enterica serovar Enteritidis | |
| SRR1635113 | Salmonella enterica subsp. enterica serovar Enteritidis | |
| SRR1635114 | Salmonella enterica subsp. enterica serovar Enteritidis | |
| SRR1635115 | Salmonella enterica subsp. enterica serovar Enteritidis | |
| SRR1635116 | Salmonella enterica subsp. enterica serovar Enteritidis | |
| SRR1635118 | Salmonella enterica subsp. enterica serovar Enteritidis | |
| SRR1635128 | Salmonella enterica subsp. enterica serovar Enteritidis | |
| SRR1786099 | Salmonella enterica subsp. enterica serovar Enteritidis str. 77-1427 | |
| SRR1786143 | Salmonella enterica subsp. enterica serovar Enteritidis str. 77-1427 | |
| SRR1786144 | Salmonella enterica subsp. enterica serovar Enteritidis str. 77-1427 | |
| SRR1792675 | Salmonella enterica subsp. enterica serovar Enteritidis str. CDC_2010K_0968 | |
| SRR1792676 | Salmonella enterica subsp. enterica serovar Enteritidis str. CDC_2010K_0968 | |
| SRR1792894 | Salmonella enterica subsp. enterica serovar Enteritidis str. CDC_2010K_0968 | |
| SRR1792895 | Salmonella enterica subsp. enterica serovar Enteritidis str. CDC_2010K_0968 | |
| SRR1792896 | Salmonella enterica subsp. enterica serovar Enteritidis str. CDC_2010K_0968 | |
| SRR1792898 | Salmonella enterica subsp. enterica serovar Enteritidis str. CDC_2010K_0968 | |
| SRR1792899 | Salmonella enterica subsp. enterica serovar Enteritidis str. CDC_2010K_0968 | |
| SRR1792900 | Salmonella enterica subsp. enterica serovar Enteritidis str. CDC_2010K_0968 | |
| SRR1792901 | Salmonella enterica subsp. enterica serovar Enteritidis str. CDC_2010K_0968 | |
| SRR1792902 | Salmonella enterica subsp. enterica serovar Enteritidis str. CDC_2010K_0968 | |
| SRR1840580 | Salmonella enterica subsp. enterica serovar Enteritidis | |
| SRR1840611 | Salmonella enterica subsp. enterica serovar Enteritidis | |
| SRR1840636 | Salmonella enterica subsp. enterica serovar Enteritidis | |
| SRR1840654 | Salmonella enterica subsp. enterica serovar Enteritidis | |
| SRR1840655 | Salmonella enterica subsp. enterica serovar Enteritidis | |
| SRR1840656 | Salmonella enterica subsp. enterica serovar Enteritidis | |
| SRR1840657 | Salmonella enterica subsp. enterica serovar Enteritidis | |
| SRR1840718 | Salmonella enterica subsp. enterica serovar Enteritidis | |
| SRR1840719 | Salmonella enterica subsp. enterica serovar Enteritidis | |
| SRR1840733 | Salmonella enterica subsp. enterica serovar Enteritidis | |
| SRR1922895 | Salmonella enterica subsp. enterica serovar Enteritidis str. 607307-2 | |
| SRR1922896 | Salmonella enterica subsp. enterica serovar Enteritidis str. 607307-2 | |
| SRR1922897 | Salmonella enterica subsp. enterica serovar Enteritidis str. 607307-2 | |
| SRR1922898 | Salmonella enterica subsp. enterica serovar Enteritidis str. 607307-2 | |
| SRR1923037 | Salmonella enterica subsp. enterica serovar Enteritidis str. 607307-2 | |
| SRR1923039 | Salmonella enterica subsp. enterica serovar Enteritidis str. 607307-2 | |
| SRR1923040 | Salmonella enterica subsp. enterica serovar Enteritidis str. 607307-2 | |
| SRR2138480 | Salmonella enterica subsp. enterica serovar Enteritidis | |
| SRR2138565 | Salmonella enterica subsp. enterica serovar Enteritidis | |
| SRR2140426 | Salmonella enterica subsp. enterica serovar Enteritidis | |
| SRR2140427 | Salmonella enterica subsp. enterica serovar Enteritidis | |
| SRR2140428 | Salmonella enterica subsp. enterica serovar Enteritidis | |
| SRR2140429 | Salmonella enterica subsp. enterica serovar Enteritidis | |
| SRR2140430 | Salmonella enterica subsp. enterica serovar Enteritidis | |
| SRR2140431 | Salmonella enterica subsp. enterica serovar Enteritidis | |
| SRR2140432 | Salmonella enterica subsp. enterica serovar Enteritidis | |
| SRR2140433 | Salmonella enterica subsp. enterica serovar Enteritidis | |
| SRR2140434 | Salmonella enterica subsp. enterica serovar Enteritidis | |
| SRR2176194 | Salmonella enterica subsp. enterica serovar Enteritidis | |
| SRR2176195 | Salmonella enterica subsp. enterica serovar Enteritidis | |
| SRR2176196 | Salmonella enterica subsp. enterica serovar Enteritidis | |
| SRR2176197 | Salmonella enterica subsp. enterica serovar Enteritidis | |
| SRR2176198 | Salmonella enterica subsp. enterica serovar Enteritidis | |
| SRR2176199 | Salmonella enterica subsp. enterica serovar Enteritidis | |
| SRR2176200 | Salmonella enterica subsp. enterica serovar Enteritidis | |
| SRR2176201 | Salmonella enterica subsp. enterica serovar Enteritidis | |
| SRR2176202 | Salmonella enterica subsp. enterica serovar Enteritidis | |
| SRR2176205 | Salmonella enterica subsp. enterica serovar Enteritidis | |
| SRR2176219 | Salmonella enterica subsp. enterica serovar Enteritidis | |
| SRR2176220 | Salmonella enterica subsp. enterica serovar Enteritidis | |
| SRR2176221 | Salmonella enterica subsp. enterica serovar Enteritidis | |
| SRR2176222 | Salmonella enterica subsp. enterica serovar Enteritidis | |
| SRR2176223 | Salmonella enterica subsp. enterica serovar Enteritidis | |
| SRR2176224 | Salmonella enterica subsp. enterica serovar Enteritidis | |
| SRR2176225 | Salmonella enterica subsp. enterica serovar Enteritidis | |
| SRR2544673 | Salmonella enterica subsp. enterica serovar Enteritidis str. 18569 | |
| SRR2566860 | Salmonella enterica subsp. enterica serovar Enteritidis | |
| SRR2566862 | Salmonella enterica subsp. enterica serovar Enteritidis | |
| SRR2566868 | Salmonella enterica subsp. enterica serovar Enteritidis | |
| SRR2566869 | Salmonella enterica subsp. enterica serovar Enteritidis | |
| SRR2566875 | Salmonella enterica subsp. enterica serovar Enteritidis | |
| SRR2566879 | Salmonella enterica subsp. enterica serovar Enteritidis | |
| SRR2566881 | Salmonella enterica subsp. enterica serovar Enteritidis | |
| SRR2566887 | Salmonella enterica subsp. enterica serovar Enteritidis | |
| SRR2566907 | Salmonella enterica subsp. enterica serovar Enteritidis | |
| SRR2566908 | Salmonella enterica subsp. enterica serovar Enteritidis | |
| SRR2566923 | Salmonella enterica subsp. enterica serovar Enteritidis | |
| SRR2566954 | Salmonella enterica subsp. enterica serovar Enteritidis | |
| SRR2566956 | Salmonella enterica subsp. enterica serovar Enteritidis | |
| SRR2566998 | Salmonella enterica subsp. enterica serovar Enteritidis | |
| SRR2567003 | Salmonella enterica subsp. enterica serovar Enteritidis | |
| SRR2567019 | Salmonella enterica subsp. enterica serovar Enteritidis | |
| SRR2567020 | Salmonella enterica subsp. enterica serovar Enteritidis | |
| SRR2567059 | Salmonella enterica subsp. enterica serovar Enteritidis | |
| SRR2567070 | Salmonella enterica subsp. enterica serovar Enteritidis | |
| SRR2567091 | Salmonella enterica subsp. enterica serovar Enteritidis | |
| SRR2567106 | Salmonella enterica subsp. enterica serovar Enteritidis | |
| SRR2567108 | Salmonella enterica subsp. enterica serovar Enteritidis | |
| SRR2567113 | Salmonella enterica subsp. enterica serovar Enteritidis | |
| SRR2567129 | Salmonella enterica subsp. enterica serovar Enteritidis | |
| SRR2567130 | Salmonella enterica subsp. enterica serovar Enteritidis | |
| SRR2567202 | Salmonella enterica subsp. enterica serovar Enteritidis | |
| SRR2567203 | Salmonella enterica subsp. enterica serovar Enteritidis | |
| SRR3110500 | Salmonella enterica subsp. enterica serovar Enteritidis | |
| SRR3203402 | Salmonella enterica subsp. enterica serovar Enteritidis str. SA19940857 | |
| SRR3205728 | Salmonella enterica subsp. enterica serovar Enteritidis str. SA19940857 | |
| SRR3226960 | Salmonella enterica subsp. enterica serovar Enteritidis | |
| SRR3226961 | Salmonella enterica subsp. enterica serovar Enteritidis | |
| SRR3664627 | Salmonella enterica subsp. enterica serovar Enteritidis | |
| SRR3665044 | Salmonella enterica subsp. enterica serovar Enteritidis | |
| SRR3665115 | Salmonella enterica subsp. enterica serovar Enteritidis | |
| SRR3665132 | Salmonella enterica subsp. enterica serovar Enteritidis | |
| SRR3904752 | Salmonella enterica subsp. enterica serovar Enteritidis | |
| SRR3904777 | Salmonella enterica subsp. enterica serovar Enteritidis | |
| SRR3907854 | Salmonella enterica subsp. enterica serovar Enteritidis | |
| SRR3907856 | Salmonella enterica subsp. enterica serovar Enteritidis | |
| SRR3908215 | Salmonella enterica subsp. enterica serovar Enteritidis | |
| SRR3910226 | Salmonella enterica subsp. enterica serovar Enteritidis | |
| SRR3910449 | Salmonella enterica subsp. enterica serovar Enteritidis | |
| SRR3910575 | Salmonella enterica subsp. enterica serovar Enteritidis | |
| SRR3910909 | Salmonella enterica subsp. enterica serovar Enteritidis | |
| SRR3910910 | Salmonella enterica subsp. enterica serovar Enteritidis | |
| SRR3910911 | Salmonella enterica subsp. enterica serovar Enteritidis | |
| SRR3910914 | Salmonella enterica subsp. enterica serovar Enteritidis | |
| SRR4114380 | Salmonella enterica subsp. enterica serovar Enteritidis | |
| SRR5017483 | Salmonella enterica subsp. enterica serovar Enteritidis | |
| SRR5063208 | Salmonella enterica subsp. enterica serovar Enteritidis | |
| SRR5063209 | Salmonella enterica subsp. enterica serovar Enteritidis | |
| SRR5063210 | Salmonella enterica subsp. enterica serovar Enteritidis | |
| SRR5063211 | Salmonella enterica subsp. enterica serovar Enteritidis | |
| SRR5063216 | Salmonella enterica subsp. enterica serovar Enteritidis | |
| SRR5064765 | Salmonella enterica subsp. enterica serovar Enteritidis | |
| SRR5064766 | Salmonella enterica subsp. enterica serovar Enteritidis | |
| SRR5064767 | Salmonella enterica subsp. enterica serovar Enteritidis | |
| SRR5064768 | Salmonella enterica subsp. enterica serovar Enteritidis | |
| SRR5064769 | Salmonella enterica subsp. enterica serovar Enteritidis | |
| SRR5064771 | Salmonella enterica subsp. enterica serovar Enteritidis | |
| SRR5064772 | Salmonella enterica subsp. enterica serovar Enteritidis | |
| SRR5064773 | Salmonella enterica subsp. enterica serovar Enteritidis | |
| SRR5064774 | Salmonella enterica subsp. enterica serovar Enteritidis | |
| SRR5064775 | Salmonella enterica subsp. enterica serovar Enteritidis | |
| SRR5064776 | Salmonella enterica subsp. enterica serovar Enteritidis | |
| SRR5064777 | Salmonella enterica subsp. enterica serovar Enteritidis | |
| SRR5064779 | Salmonella enterica subsp. enterica serovar Enteritidis | |
| SRR5064781 | Salmonella enterica subsp. enterica serovar Enteritidis | |
| SRR5064782 | Salmonella enterica subsp. enterica serovar Enteritidis | |
| SRR5064783 | Salmonella enterica subsp. enterica serovar Enteritidis | |
| SRR5064784 | Salmonella enterica subsp. enterica serovar Enteritidis | |
| SRR5064785 | Salmonella enterica subsp. enterica serovar Enteritidis | |
| SRR5064786 | Salmonella enterica subsp. enterica serovar Enteritidis | |
| SRR5064787 | Salmonella enterica subsp. enterica serovar Enteritidis | |
| SRR5064788 | Salmonella enterica subsp. enterica serovar Enteritidis | |
| SRR5064790 | Salmonella enterica subsp. enterica serovar Enteritidis | |
| SRR5064792 | Salmonella enterica subsp. enterica serovar Enteritidis | |
| SRR5064794 | Salmonella enterica subsp. enterica serovar Enteritidis | |
| SRR5064795 | Salmonella enterica subsp. enterica serovar Enteritidis | |
| SRR5064797 | Salmonella enterica subsp. enterica serovar Enteritidis | |
| SRR5064799 | Salmonella enterica subsp. enterica serovar Enteritidis | |
| SRR5064800 | Salmonella enterica subsp. enterica serovar Enteritidis | |
| SRR5064801 | Salmonella enterica subsp. enterica serovar Enteritidis | |
| SRR5064802 | Salmonella enterica subsp. enterica serovar Enteritidis | |
| SRR5064803 | Salmonella enterica subsp. enterica serovar Enteritidis | |
| SRR5064804 | Salmonella enterica subsp. enterica serovar Enteritidis | |
| SRR5064806 | Salmonella enterica subsp. enterica serovar Enteritidis | |
| SRR5064809 | Salmonella enterica subsp. enterica serovar Enteritidis | |
| SRR5064810 | Salmonella enterica subsp. enterica serovar Enteritidis | |
| SRR5064811 | Salmonella enterica subsp. enterica serovar Enteritidis | |
| SRR5064812 | Salmonella enterica subsp. enterica serovar Enteritidis | |
| SRR5064813 | Salmonella enterica subsp. enterica serovar Enteritidis | |
| SRR5064814 | Salmonella enterica subsp. enterica serovar Enteritidis | |
| SRR5064815 | Salmonella enterica subsp. enterica serovar Enteritidis | |
| SRR5064816 | Salmonella enterica subsp. enterica serovar Enteritidis | |
| SRR5064817 | Salmonella enterica subsp. enterica serovar Enteritidis | |
| SRR5064818 | Salmonella enterica subsp. enterica serovar Enteritidis | |
| SRR5064820 | Salmonella enterica subsp. enterica serovar Enteritidis | |
| SRR5064821 | Salmonella enterica subsp. enterica serovar Enteritidis | |
| SRR5064854 | Salmonella enterica subsp. enterica serovar Enteritidis | |
| SRR5064855 | Salmonella enterica subsp. enterica serovar Enteritidis | |
| SRR5064857 | Salmonella enterica subsp. enterica serovar Enteritidis | |
| SRR5065189 | Salmonella enterica subsp. enterica serovar Enteritidis | |
| SRR5065190 | Salmonella enterica subsp. enterica serovar Enteritidis | |
| SRR5065191 | Salmonella enterica subsp. enterica serovar Enteritidis | |
| SRR5065192 | Salmonella enterica subsp. enterica serovar Enteritidis | |
| SRR5065193 | Salmonella enterica subsp. enterica serovar Enteritidis | |
| SRR5065194 | Salmonella enterica subsp. enterica serovar Enteritidis | |
| SRR5065195 | Salmonella enterica subsp. enterica serovar Enteritidis | |
| SRR5065196 | Salmonella enterica subsp. enterica serovar Enteritidis | |
| SRR5065197 | Salmonella enterica subsp. enterica serovar Enteritidis | |
| SRR5065198 | Salmonella enterica subsp. enterica serovar Enteritidis | |
| SRR5065199 | Salmonella enterica subsp. enterica serovar Enteritidis | |
| SRR5126097 | Salmonella enterica subsp. enterica serovar Enteritidis | |
| SRR5126098 | Salmonella enterica subsp. enterica serovar Enteritidis | |
| SRR5126099 | Salmonella enterica subsp. enterica serovar Enteritidis | |
| SRR5126100 | Salmonella enterica subsp. enterica serovar Enteritidis | |
| SRR5126101 | Salmonella enterica subsp. enterica serovar Enteritidis | |
| SRR5126102 | Salmonella enterica subsp. enterica serovar Enteritidis | |
| SRR5126104 | Salmonella enterica subsp. enterica serovar Enteritidis | |
| SRR5126105 | Salmonella enterica subsp. enterica serovar Enteritidis | |
| SRR5126106 | Salmonella enterica subsp. enterica serovar Enteritidis | |
| SRR5126107 | Salmonella enterica subsp. enterica serovar Enteritidis | |
| SRR5126108 | Salmonella enterica subsp. enterica serovar Enteritidis | |
| SRR5126109 | Salmonella enterica subsp. enterica serovar Enteritidis | |
| SRR5126110 | Salmonella enterica subsp. enterica serovar Enteritidis | |
| SRR5126111 | Salmonella enterica subsp. enterica serovar Enteritidis | |
| SRR5126112 | Salmonella enterica subsp. enterica serovar Enteritidis | |
| SRR5126113 | Salmonella enterica subsp. enterica serovar Enteritidis | |
| SRR5126114 | Salmonella enterica subsp. enterica serovar Enteritidis | |
| SRR5126115 | Salmonella enterica subsp. enterica serovar Enteritidis | |
| SRR5126116 | Salmonella enterica subsp. enterica serovar Enteritidis | |
| SRR5126183 | Salmonella enterica subsp. enterica serovar Enteritidis | |
| SRR5126184 | Salmonella enterica subsp. enterica serovar Enteritidis | |
| SRR5126185 | Salmonella enterica subsp. enterica serovar Enteritidis | |
| SRR5126186 | Salmonella enterica subsp. enterica serovar Enteritidis | |
| SRR5126187 | Salmonella enterica subsp. enterica serovar Enteritidis | |
| SRR5126188 | Salmonella enterica subsp. enterica serovar Enteritidis | |
| SRR5126189 | Salmonella enterica subsp. enterica serovar Enteritidis | |
| SRR5126190 | Salmonella enterica subsp. enterica serovar Enteritidis | |
| SRR5126191 | Salmonella enterica subsp. enterica serovar Enteritidis | |
| SRR5126197 | Salmonella enterica subsp. enterica serovar Enteritidis | |
| SRR5126198 | Salmonella enterica subsp. enterica serovar Enteritidis | |
| SRR5126199 | Salmonella enterica subsp. enterica serovar Enteritidis | |
| SRR5126200 | Salmonella enterica subsp. enterica serovar Enteritidis | |
| SRR5126201 | Salmonella enterica subsp. enterica serovar Enteritidis | |
| SRR5126202 | Salmonella enterica subsp. enterica serovar Enteritidis | |
| SRR5126203 | Salmonella enterica subsp. enterica serovar Enteritidis | |
| SRR5126204 | Salmonella enterica subsp. enterica serovar Enteritidis | |
| SRR5126205 | Salmonella enterica subsp. enterica serovar Enteritidis | |
| SRR5126206 | Salmonella enterica subsp. enterica serovar Enteritidis | |
| SRR5126207 | Salmonella enterica subsp. enterica serovar Enteritidis | |
| SRR5126208 | Salmonella enterica subsp. enterica serovar Enteritidis | |
| SRR5126209 | Salmonella enterica subsp. enterica serovar Enteritidis | |
| SRR5126210 | Salmonella enterica subsp. enterica serovar Enteritidis | |
| SRR5126211 | Salmonella enterica subsp. enterica serovar Enteritidis | |
| SRR5126212 | Salmonella enterica subsp. enterica serovar Enteritidis | |
| SRR5126213 | Salmonella enterica subsp. enterica serovar Enteritidis | |
| SRR5126214 | Salmonella enterica subsp. enterica serovar Enteritidis | |
| SRR5126215 | Salmonella enterica subsp. enterica serovar Enteritidis | |
| SRR5126216 | Salmonella enterica subsp. enterica serovar Enteritidis | |
| SRR5126217 | Salmonella enterica subsp. enterica serovar Enteritidis | |
| SRR5126218 | Salmonella enterica subsp. enterica serovar Enteritidis | |
| SRR5126219 | Salmonella enterica subsp. enterica serovar Enteritidis | |
| SRR5126220 | Salmonella enterica subsp. enterica serovar Enteritidis | |
| SRR5126221 | Salmonella enterica subsp. enterica serovar Enteritidis | |
| SRR5126222 | Salmonella enterica subsp. enterica serovar Enteritidis | |
| SRR5126223 | Salmonella enterica subsp. enterica serovar Enteritidis | |
| SRR5126224 | Salmonella enterica subsp. enterica serovar Enteritidis | |
| SRR5126225 | Salmonella enterica subsp. enterica serovar Enteritidis | |
| SRR5126226 | Salmonella enterica subsp. enterica serovar Enteritidis | |
| SRR5126227 | Salmonella enterica subsp. enterica serovar Enteritidis | |
| SRR5126459 | Salmonella enterica subsp. enterica serovar Enteritidis | |
| SRR5126929 | Salmonella enterica subsp. enterica serovar Enteritidis | |
| SRR5126930 | Salmonella enterica subsp. enterica serovar Enteritidis | |
| SRR5126931 | Salmonella enterica subsp. enterica serovar Enteritidis | |
| SRR5126932 | Salmonella enterica subsp. enterica serovar Enteritidis | |
| SRR5126933 | Salmonella enterica subsp. enterica serovar Enteritidis | |
| SRR5126934 | Salmonella enterica subsp. enterica serovar Enteritidis | |
| SRR5126935 | Salmonella enterica subsp. enterica serovar Enteritidis | |
| SRR5126936 | Salmonella enterica subsp. enterica serovar Enteritidis | |
| SRR5126937 | Salmonella enterica subsp. enterica serovar Enteritidis | |
| SRR5126938 | Salmonella enterica subsp. enterica serovar Enteritidis | |
| SRR5126939 | Salmonella enterica subsp. enterica serovar Enteritidis | |
| SRR5126940 | Salmonella enterica subsp. enterica serovar Enteritidis | |
| SRR5126941 | Salmonella enterica subsp. enterica serovar Enteritidis | |
| SRR5126942 | Salmonella enterica subsp. enterica serovar Enteritidis | |
| SRR5126943 | Salmonella enterica subsp. enterica serovar Enteritidis | |
| SRR5126944 | Salmonella enterica subsp. enterica serovar Enteritidis | |
| SRR5126945 | Salmonella enterica subsp. enterica serovar Enteritidis | |
| SRR5126946 | Salmonella enterica subsp. enterica serovar Enteritidis | |
| SRR5126947 | Salmonella enterica subsp. enterica serovar Enteritidis | |
| SRR5126948 | Salmonella enterica subsp. enterica serovar Enteritidis | |
| SRR5126949 | Salmonella enterica subsp. enterica serovar Enteritidis | |
| SRR5126950 | Salmonella enterica subsp. enterica serovar Enteritidis | |
| SRR5126951 | Salmonella enterica subsp. enterica serovar Enteritidis | |
| SRR5126952 | Salmonella enterica subsp. enterica serovar Enteritidis | |
| SRR5126953 | Salmonella enterica subsp. enterica serovar Enteritidis | |
| SRR5126954 | Salmonella enterica subsp. enterica serovar Enteritidis | |
| SRR5126955 | Salmonella enterica subsp. enterica serovar Enteritidis | |
| SRR5126956 | Salmonella enterica subsp. enterica serovar Enteritidis | |
| SRR5126957 | Salmonella enterica subsp. enterica serovar Enteritidis | |
| SRR5126958 | Salmonella enterica subsp. enterica serovar Enteritidis | |
| SRR5126959 | Salmonella enterica subsp. enterica serovar Enteritidis | |
| SRR5126960 | Salmonella enterica subsp. enterica serovar Enteritidis | |
| SRR5126961 | Salmonella enterica subsp. enterica serovar Enteritidis | |
| SRR5126962 | Salmonella enterica subsp. enterica serovar Enteritidis | |
| SRR5126963 | Salmonella enterica subsp. enterica serovar Enteritidis | |
| SRR5126964 | Salmonella enterica subsp. enterica serovar Enteritidis | |
| SRR5126965 | Salmonella enterica subsp. enterica serovar Enteritidis | |
| SRR5126966 | Salmonella enterica subsp. enterica serovar Enteritidis | |
| SRR5126967 | Salmonella enterica subsp. enterica serovar Enteritidis | |
| SRR5126968 | Salmonella enterica subsp. enterica serovar Enteritidis | |
| SRR5126969 | Salmonella enterica subsp. enterica serovar Enteritidis | |
| SRR5126970 | Salmonella enterica subsp. enterica serovar Enteritidis | |
| SRR5126971 | Salmonella enterica subsp. enterica serovar Enteritidis | |
| SRR5126972 | Salmonella enterica subsp. enterica serovar Enteritidis | |
| SRR5126973 | Salmonella enterica subsp. enterica serovar Enteritidis | |
| SRR5126974 | Salmonella enterica subsp. enterica serovar Enteritidis | |
| SRR5126975 | Salmonella enterica subsp. enterica serovar Enteritidis | |
| SRR5126976 | Salmonella enterica subsp. enterica serovar Enteritidis | |
| SRR5126977 | Salmonella enterica subsp. enterica serovar Enteritidis | |
| SRR5126978 | Salmonella enterica subsp. enterica serovar Enteritidis | |
| SRR5126979 | Salmonella enterica subsp. enterica serovar Enteritidis | |
| SRR5126980 | Salmonella enterica subsp. enterica serovar Enteritidis | |
| SRR5126981 | Salmonella enterica subsp. enterica serovar Enteritidis | |
| SRR5126982 | Salmonella enterica subsp. enterica serovar Enteritidis | |
| SRR5126983 | Salmonella enterica subsp. enterica serovar Enteritidis | |
| SRR5126984 | Salmonella enterica subsp. enterica serovar Enteritidis | |
| SRR5126985 | Salmonella enterica subsp. enterica serovar Enteritidis | |
| SRR5126986 | Salmonella enterica subsp. enterica serovar Enteritidis | |
| SRR5126987 | Salmonella enterica subsp. enterica serovar Enteritidis | |
| SRR5126988 | Salmonella enterica subsp. enterica serovar Enteritidis | |
| SRR5126989 | Salmonella enterica subsp. enterica serovar Enteritidis | |
| SRR5126990 | Salmonella enterica subsp. enterica serovar Enteritidis | |
| SRR5126991 | Salmonella enterica subsp. enterica serovar Enteritidis | |
| SRR5126992 | Salmonella enterica subsp. enterica serovar Enteritidis | |
| SRR5126993 | Salmonella enterica subsp. enterica serovar Enteritidis | |
| SRR5126994 | Salmonella enterica subsp. enterica serovar Enteritidis | |
| SRR5126995 | Salmonella enterica subsp. enterica serovar Enteritidis | |
| SRR5126996 | Salmonella enterica subsp. enterica serovar Enteritidis | |
| SRR5126997 | Salmonella enterica subsp. enterica serovar Enteritidis | |
| SRR5126998 | Salmonella enterica subsp. enterica serovar Enteritidis | |
| SRR5126999 | Salmonella enterica subsp. enterica serovar Enteritidis | |
| SRR5127000 | Salmonella enterica subsp. enterica serovar Enteritidis | |
| SRR5159825 | Salmonella enterica subsp. enterica serovar Enteritidis | |
| SRR5159854 | Salmonella enterica subsp. enterica serovar Enteritidis | |
| SRR5159883 | Salmonella enterica subsp. enterica serovar Enteritidis | |
| SRR5160144 | Salmonella enterica subsp. enterica serovar Enteritidis | |
| SRR5160149 | Salmonella enterica subsp. enterica serovar Enteritidis | |
| SRR5160152 | Salmonella enterica subsp. enterica serovar Enteritidis | |
| SRR5160221 | Salmonella enterica subsp. enterica serovar Enteritidis | |
| SRR5160233 | Salmonella enterica subsp. enterica serovar Enteritidis | |
| SRR5160235 | Salmonella enterica subsp. enterica serovar Enteritidis | |
| SRR5160237 | Salmonella enterica subsp. enterica serovar Enteritidis | |
| SRR5160242 | Salmonella enterica subsp. enterica serovar Enteritidis | |
| SRR5160269 | Salmonella enterica subsp. enterica serovar Enteritidis | |
| SRR5160270 | Salmonella enterica subsp. enterica serovar Enteritidis | |
| SRR5160271 | Salmonella enterica subsp. enterica serovar Enteritidis | |
| SRR5160272 | Salmonella enterica subsp. enterica serovar Enteritidis | |
| SRR5160274 | Salmonella enterica subsp. enterica serovar Enteritidis | |
| SRR5160276 | Salmonella enterica subsp. enterica serovar Enteritidis | |
| SRR5160277 | Salmonella enterica subsp. enterica serovar Enteritidis | |
| SRR5160280 | Salmonella enterica subsp. enterica serovar Enteritidis | |
| SRR5160281 | Salmonella enterica subsp. enterica serovar Enteritidis | |
| SRR5160284 | Salmonella enterica subsp. enterica serovar Enteritidis | |
| SRR5160289 | Salmonella enterica subsp. enterica serovar Enteritidis | |
| SRR5160290 | Salmonella enterica subsp. enterica serovar Enteritidis | |
| SRR5160291 | Salmonella enterica subsp. enterica serovar Enteritidis | |
| SRR5160292 | Salmonella enterica subsp. enterica serovar Enteritidis | |
| SRR5160293 | Salmonella enterica subsp. enterica serovar Enteritidis | |
| SRR5160294 | Salmonella enterica subsp. enterica serovar Enteritidis | |
| SRR5160295 | Salmonella enterica subsp. enterica serovar Enteritidis | |
| SRR5160296 | Salmonella enterica subsp. enterica serovar Enteritidis | |
| SRR5160297 | Salmonella enterica subsp. enterica serovar Enteritidis | |
| SRR5160298 | Salmonella enterica subsp. enterica serovar Enteritidis | |
| SRR5160299 | Salmonella enterica subsp. enterica serovar Enteritidis | |
| SRR5160300 | Salmonella enterica subsp. enterica serovar Enteritidis | |
| SRR5160301 | Salmonella enterica subsp. enterica serovar Enteritidis | |
| SRR5160302 | Salmonella enterica subsp. enterica serovar Enteritidis | |
| SRR5160303 | Salmonella enterica subsp. enterica serovar Enteritidis | |
| SRR5160306 | Salmonella enterica subsp. enterica serovar Enteritidis | |
| SRR5160307 | Salmonella enterica subsp. enterica serovar Enteritidis | |
| SRR5160308 | Salmonella enterica subsp. enterica serovar Enteritidis | |
| SRR5160309 | Salmonella enterica subsp. enterica serovar Enteritidis | |
| SRR5160310 | Salmonella enterica subsp. enterica serovar Enteritidis | |
| SRR5160311 | Salmonella enterica subsp. enterica serovar Enteritidis | |
| SRR5160312 | Salmonella enterica subsp. enterica serovar Enteritidis | |
| SRR5160313 | Salmonella enterica subsp. enterica serovar Enteritidis | |
| SRR5160314 | Salmonella enterica subsp. enterica serovar Enteritidis | |
| SRR5160317 | Salmonella enterica subsp. enterica serovar Enteritidis | |
| SRR5160318 | Salmonella enterica subsp. enterica serovar Enteritidis | |
| SRR5160319 | Salmonella enterica subsp. enterica serovar Enteritidis | |
| SRR5160320 | Salmonella enterica subsp. enterica serovar Enteritidis | |
| SRR5160321 | Salmonella enterica subsp. enterica serovar Enteritidis | |
| SRR5160322 | Salmonella enterica subsp. enterica serovar Enteritidis | |
| SRR5160323 | Salmonella enterica subsp. enterica serovar Enteritidis | |
| SRR5160324 | Salmonella enterica subsp. enterica serovar Enteritidis | |
| SRR5160325 | Salmonella enterica subsp. enterica serovar Enteritidis | |
| SRR5160326 | Salmonella enterica subsp. enterica serovar Enteritidis | |
| SRR5160381 | Salmonella enterica subsp. enterica serovar Enteritidis | |
| SRR5160382 | Salmonella enterica subsp. enterica serovar Enteritidis | |
| SRR5160383 | Salmonella enterica subsp. enterica serovar Enteritidis | |
| SRR5160384 | Salmonella enterica subsp. enterica serovar Enteritidis | |
| SRR5160385 | Salmonella enterica subsp. enterica serovar Enteritidis | |
| SRR5160386 | Salmonella enterica subsp. enterica serovar Enteritidis | |
| SRR5160387 | Salmonella enterica subsp. enterica serovar Enteritidis | |
| SRR5160388 | Salmonella enterica subsp. enterica serovar Enteritidis | |
| SRR5160389 | Salmonella enterica subsp. enterica serovar Enteritidis | |
| SRR5160408 | Salmonella enterica subsp. enterica serovar Enteritidis | |
| SRR5160409 | Salmonella enterica subsp. enterica serovar Enteritidis | |
| SRR5160413 | Salmonella enterica subsp. enterica serovar Enteritidis | |
| SRR5160414 | Salmonella enterica subsp. enterica serovar Enteritidis | |
| SRR5160416 | Salmonella enterica subsp. enterica serovar Enteritidis | |
| SRR5160417 | Salmonella enterica subsp. enterica serovar Enteritidis | |
| SRR5160418 | Salmonella enterica subsp. enterica serovar Enteritidis | |
| SRR5160419 | Salmonella enterica subsp. enterica serovar Enteritidis | |
| SRR5160420 | Salmonella enterica subsp. enterica serovar Enteritidis | |
| SRR5160421 | Salmonella enterica subsp. enterica serovar Enteritidis | |
| SRR5160422 | Salmonella enterica subsp. enterica serovar Enteritidis | |
| SRR5160423 | Salmonella enterica subsp. enterica serovar Enteritidis | |
| SRR5160424 | Salmonella enterica subsp. enterica serovar Enteritidis | |
| SRR5160425 | Salmonella enterica subsp. enterica serovar Enteritidis | |
| SRR5160519 | Salmonella enterica subsp. enterica serovar Enteritidis | |
| SRR5160520 | Salmonella enterica subsp. enterica serovar Enteritidis | |
| SRR5160521 | Salmonella enterica subsp. enterica serovar Enteritidis | |
| SRR5160522 | Salmonella enterica subsp. enterica serovar Enteritidis | |
| SRR5160523 | Salmonella enterica subsp. enterica serovar Enteritidis | |
| SRR5160524 | Salmonella enterica subsp. enterica serovar Enteritidis | |
| SRR5160525 | Salmonella enterica subsp. enterica serovar Enteritidis | |
| SRR5160526 | Salmonella enterica subsp. enterica serovar Enteritidis | |
| SRR5160527 | Salmonella enterica subsp. enterica serovar Enteritidis | |
| SRR5160531 | Salmonella enterica subsp. enterica serovar Enteritidis | |
| SRR5160532 | Salmonella enterica subsp. enterica serovar Enteritidis | |
| SRR5160545 | Salmonella enterica subsp. enterica serovar Enteritidis | |
| SRR5160549 | Salmonella enterica subsp. enterica serovar Enteritidis | |
| SRR5160633 | Salmonella enterica subsp. enterica serovar Enteritidis | |
| SRR5160634 | Salmonella enterica subsp. enterica serovar Enteritidis | |
| SRR5160635 | Salmonella enterica subsp. enterica serovar Enteritidis | |
| SRR5160638 | Salmonella enterica subsp. enterica serovar Enteritidis | |
| SRR5160639 | Salmonella enterica subsp. enterica serovar Enteritidis | |
| SRR5160640 | Salmonella enterica subsp. enterica serovar Enteritidis | |
| SRR5160641 | Salmonella enterica subsp. enterica serovar Enteritidis | |
| SRR5160642 | Salmonella enterica subsp. enterica serovar Enteritidis | |
| SRR5160643 | Salmonella enterica subsp. enterica serovar Enteritidis | |
| SRR5160644 | Salmonella enterica subsp. enterica serovar Enteritidis | |
| SRR5160645 | Salmonella enterica subsp. enterica serovar Enteritidis | |
| SRR5160646 | Salmonella enterica subsp. enterica serovar Enteritidis | |
| SRR5160647 | Salmonella enterica subsp. enterica serovar Enteritidis | |
| SRR5160648 | Salmonella enterica subsp. enterica serovar Enteritidis | |
| SRR5160649 | Salmonella enterica subsp. enterica serovar Enteritidis | |
| SRR5160650 | Salmonella enterica subsp. enterica serovar Enteritidis | |
| SRR5160658 | Salmonella enterica subsp. enterica serovar Enteritidis | |
| SRR5160660 | Salmonella enterica subsp. enterica serovar Enteritidis | |
| SRR5160663 | Salmonella enterica subsp. enterica serovar Enteritidis | |
| SRR5160664 | Salmonella enterica subsp. enterica serovar Enteritidis | |
| SRR5160665 | Salmonella enterica subsp. enterica serovar Enteritidis | |
| SRR5160666 | Salmonella enterica subsp. enterica serovar Enteritidis | |
| SRR5160668 | Salmonella enterica subsp. enterica serovar Enteritidis | |
| SRR516354 | Salmonella enterica subsp. enterica serovar Enteritidis str. 48-0811 | |
| SRR516355 | Salmonella enterica subsp. enterica serovar Enteritidis str. 48-0811 | |
| SRR516360 | Salmonella enterica subsp. enterica serovar Enteritidis str. 48-0811 | |
| SRR516367 | Salmonella enterica subsp. enterica serovar Enteritidis str. 48-0811 | |
| SRR516368 | Salmonella enterica subsp. enterica serovar Enteritidis str. SARB19 | |
| SRR516371 | Salmonella enterica subsp. enterica serovar Enteritidis str. SARB19 | |
| SRR516372 | Salmonella enterica subsp. enterica serovar Enteritidis str. SARB19 | |
| SRR516377 | Salmonella enterica subsp. enterica serovar Enteritidis str. SARB19 | |
| SRR518748 | Salmonella enterica subsp. enterica serovar Enteritidis str. CDC_2010K_1580 | |
| SRR518749 | Salmonella enterica subsp. enterica serovar Enteritidis str. CDC_2010K_1543 | |
| SRR518750 | Salmonella enterica subsp. enterica serovar Enteritidis str. CDC_2010K_1810 | |
| SRR518751 | Salmonella enterica subsp. enterica serovar Enteritidis str. CDC_2010K_1808 | |
| SRR518752 | Salmonella enterica subsp. enterica serovar Enteritidis str. CDC_2010K_1795 | |
| SRR518753 | Salmonella enterica subsp. enterica serovar Enteritidis str. 6.0562-1 | |
| SRR518754 | Salmonella enterica subsp. enterica serovar Enteritidis str. 50-5646 | |
| SRR518755 | Salmonella enterica subsp. enterica serovar Enteritidis str. 596866-70 | |
| SRR518756 | Salmonella enterica subsp. enterica serovar Enteritidis str. 629164-26 | |
| SRR518757 | Salmonella enterica subsp. enterica serovar Enteritidis str. 629164-37 | |
| SRR518758 | Salmonella enterica subsp. enterica serovar Enteritidis str. 22558 | |
| SRR518759 | Salmonella enterica subsp. enterica serovar Enteritidis str. 33944 | |
| SRR518760 | Salmonella enterica subsp. enterica serovar Enteritidis str. 543463 22-17 | |
| SRR518761 | Salmonella enterica subsp. enterica serovar Enteritidis str. 543463 40-18 | |
| SRR518762 | Salmonella enterica subsp. enterica serovar Enteritidis str. 561362 1-1 | |
| SRR518763 | Salmonella enterica subsp. enterica serovar Enteritidis str. 648901 6-18 | |
| SRR518764 | Salmonella enterica subsp. enterica serovar Enteritidis str. 642044 8-1 | |
| SRR518765 | Salmonella enterica subsp. enterica serovar Enteritidis str. 543463 42-20 | |
| SRR518766 | Salmonella enterica subsp. enterica serovar Enteritidis str. CDC_2010K_1565 | |
| SRR518767 | Salmonella enterica subsp. enterica serovar Enteritidis str. 8b-1 | |
| SRR518768 | Salmonella enterica subsp. enterica serovar Enteritidis str. 576709 | |
| SRR518769 | Salmonella enterica subsp. enterica serovar Enteritidis str. 576709 | |
| SRR518770 | Salmonella enterica subsp. enterica serovar Enteritidis str. 639672-46 | |
| SRR518771 | Salmonella enterica subsp. enterica serovar Enteritidis str. CDC_2010K_1441 | |
| SRR518772 | Salmonella enterica subsp. enterica serovar Enteritidis str. CDC_2010K_1811 | |
| SRR518773 | Salmonella enterica subsp. enterica serovar Enteritidis str. 561362 9-7 | |
| SRR518774 | Salmonella enterica subsp. enterica serovar Enteritidis str. SE10 | |
| SRR518775 | Salmonella enterica subsp. enterica serovar Enteritidis str. 436 | |
| SRR518776 | Salmonella enterica subsp. enterica serovar Enteritidis str. CDC_2010K_1594 | |
| SRR518777 | Salmonella enterica subsp. enterica serovar Enteritidis str. CDC_2010K_1594 | |
| SRR518778 | Salmonella enterica subsp. enterica serovar Enteritidis str. CDC_2010K_1018 | |
| SRR518779 | Salmonella enterica subsp. enterica serovar Enteritidis str. CDC_2010K_1729 | |
| SRR518780 | Salmonella enterica subsp. enterica serovar Enteritidis str. CDC_2010K_0968 | |
| SRR518781 | Salmonella enterica subsp. enterica serovar Enteritidis str. CDC_2010K_1445 | |
| SRR518782 | Salmonella enterica subsp. enterica serovar Enteritidis str. CDC_2010K_1455 | |
| SRR518783 | Salmonella enterica subsp. enterica serovar Enteritidis str. CDC_2010K_1725 | |
| SRR518784 | Salmonella enterica subsp. enterica serovar Enteritidis str. 22510-1 | |
| SRR518785 | Salmonella enterica subsp. enterica serovar Enteritidis str. SE15-1 | |
| SRR518786 | Salmonella enterica subsp. enterica serovar Enteritidis str. 622731-39 | |
| SRR518787 | Salmonella enterica subsp. enterica serovar Enteritidis str. 629163 | |
| SRR518788 | Salmonella enterica subsp. enterica serovar Enteritidis str. 485549-17 | |
| SRR518789 | Salmonella enterica subsp. enterica serovar Enteritidis str. CVM_56-3991 | |
| SRR518790 | Salmonella enterica subsp. enterica serovar Enteritidis str. CVM_69-4941 | |
| SRR518791 | Salmonella enterica subsp. enterica serovar Enteritidis str. CVM_69-4941 | |
| SRR518792 | Salmonella enterica subsp. enterica serovar Enteritidis str. 17927 | |
| SRR518793 | Salmonella enterica subsp. enterica serovar Enteritidis str. 642044 4-1 | |
| SRR518794 | Salmonella enterica subsp. enterica serovar Enteritidis str. 648904 3-6 | |
| SRR518795 | Salmonella enterica subsp. enterica serovar Enteritidis str. 648901 16-16 | |
| SRR518796 | Salmonella enterica subsp. enterica serovar Enteritidis str. 53-407 | |
| SRR518797 | Salmonella enterica subsp. enterica serovar Enteritidis str. 81-2625 | |
| SRR518798 | Salmonella enterica subsp. enterica serovar Enteritidis str. CDC_2010K_1882 | |
| SRR518799 | Salmonella enterica subsp. enterica serovar Enteritidis str. 648898 4-5 | |
| SRR518800 | Salmonella enterica subsp. enterica serovar Enteritidis str. 640631 | |
| SRR518802 | Salmonella enterica subsp. enterica serovar Enteritidis str. 76-2651 | |
| SRR518803 | Salmonella enterica subsp. enterica serovar Enteritidis str. 653049 13-19 | |
| SRR518804 | Salmonella enterica subsp. enterica serovar Enteritidis str. 20037 | |
| SRR518805 | Salmonella enterica subsp. enterica serovar Enteritidis str. CHS44 | |
| SRR518806 | Salmonella enterica subsp. enterica serovar Enteritidis str. CDC_2010K_1884 | |
| SRR518807 | Salmonella enterica subsp. enterica serovar Enteritidis str. CDC_2010K_1558 | |
| SRR518808 | Salmonella enterica subsp. enterica serovar Enteritidis str. CDC_2010K_1010 | |
| SRR518809 | Salmonella enterica subsp. enterica serovar Enteritidis str. CDC_2010K_1559 | |
| SRR518810 | Salmonella enterica subsp. enterica serovar Enteritidis str. CDC_2010K_1745 | |
| SRR518811 | Salmonella enterica subsp. enterica serovar Enteritidis str. 78-1757 | |
| SRR518812 | Salmonella enterica subsp. enterica serovar Enteritidis str. 13183-1 | |
| SRR518813 | Salmonella enterica subsp. enterica serovar Enteritidis str. 639016-6 | |
| SRR518814 | Salmonella enterica subsp. enterica serovar Enteritidis str. 635290-58 | |
| SRR518815 | Salmonella enterica subsp. enterica serovar Enteritidis str. SARB17 | |
| SRR518816 | Salmonella enterica subsp. enterica serovar Enteritidis str. 596866-22 | |
| SRR518817 | Salmonella enterica subsp. enterica serovar Enteritidis str. 596866-22 | |
| SRR518818 | Salmonella enterica subsp. enterica serovar Enteritidis str. 639672-50 | |
| SRR518819 | Salmonella enterica subsp. enterica serovar Enteritidis str. 638970-15 | |
| SRR518820 | Salmonella enterica subsp. enterica serovar Enteritidis str. 638970-15 | |
| SRR518821 | Salmonella enterica subsp. enterica serovar Enteritidis str. 642046 4-7 | |
| SRR518822 | Salmonella enterica subsp. enterica serovar Enteritidis str. 648899 3-17 | |
| SRR518823 | Salmonella enterica subsp. enterica serovar Enteritidis str. 648905 5-18 | |
| SRR518824 | Salmonella enterica subsp. enterica serovar Enteritidis str. 50-3079 | |
| SRR518825 | Salmonella enterica subsp. enterica serovar Enteritidis str. 58-6482 | |
| SRR518826 | Salmonella enterica subsp. enterica serovar Enteritidis str. 18569 | |
| SRR518827 | Salmonella enterica subsp. enterica serovar Enteritidis str. CDC_2010K_1444 | |
| SRR518828 | Salmonella enterica subsp. enterica serovar Enteritidis str. CVM_N202 | |
| SRR518829 | Salmonella enterica subsp. enterica serovar Enteritidis str. 648903 1-6 | |
| SRR518830 | Salmonella enterica subsp. enterica serovar Enteritidis str. CDC_2010K_1566 | |
| SRR518831 | Salmonella enterica subsp. enterica serovar Enteritidis str. CDC_2010K_1575 | |
| SRR518832 | Salmonella enterica subsp. enterica serovar Enteritidis str. CHS4 | |
| SRR518833 | Salmonella enterica subsp. enterica serovar Enteritidis str. 13-1 | |
| SRR518834 | Salmonella enterica subsp. enterica serovar Enteritidis str. PT23 | |
| SRR518835 | Salmonella enterica subsp. enterica serovar Enteritidis str. SE30663 | |
| SRR518836 | Salmonella enterica subsp. enterica serovar Enteritidis str. CDC_2010K_0899 | |
| SRR518837 | Salmonella enterica subsp. enterica serovar Enteritidis str. CDC_2010K_0895 | |
| SRR518838 | Salmonella enterica subsp. enterica serovar Enteritidis str. CDC_2010K_1747 | |
| SRR518839 | Salmonella enterica subsp. enterica serovar Enteritidis str. CDC_2010K_1791 | |
| SRR518840 | Salmonella enterica subsp. enterica serovar Enteritidis str. 77-0424 | |
| SRR518841 | Salmonella enterica subsp. enterica serovar Enteritidis str. 77-1427 | |
| SRR518843 | Salmonella enterica subsp. enterica serovar Enteritidis str. 77-2659 | |
| SRR518844 | Salmonella enterica subsp. enterica serovar Enteritidis str. 607308-16 | |
| SRR518845 | Salmonella enterica subsp. enterica serovar Enteritidis str. 607307-2 | |
| SRR518846 | Salmonella enterica subsp. enterica serovar Enteritidis str. 607308-9 | |
| SRR518847 | Salmonella enterica subsp. enterica serovar Enteritidis str. CVM_76-3618 | |
| SRR518848 | Salmonella enterica subsp. enterica serovar Enteritidis str. SL913 | |
| SRR518849 | Salmonella enterica subsp. enterica serovar Enteritidis str. SL909 | |
| SRR518850 | Salmonella enterica subsp. enterica serovar Enteritidis str. 648900 1-16 | |
| SRR518851 | Salmonella enterica subsp. enterica serovar Enteritidis str. 648901 39-2 | |
| SRR518852 | Salmonella enterica subsp. enterica serovar Enteritidis str. 648902 6-8 | |
| SRR518853 | Salmonella enterica subsp. enterica serovar Enteritidis str. SE8a | |
| SRR518854 | Salmonella enterica subsp. enterica serovar Enteritidis str. 22704 | |
| SRR518855 | Salmonella enterica subsp. enterica serovar Enteritidis str. CDC_2010K_1457 | |
| SRR518856 | Salmonella enterica subsp. enterica serovar Enteritidis str. 648901 1-17 | |
| SRR518857 | Salmonella enterica subsp. enterica serovar Enteritidis str. 62-1976 | |
| SRR518858 | Salmonella enterica subsp. enterica serovar Enteritidis str. 607308-19 | |
| SRR518859 | Salmonella enterica subsp. enterica serovar Enteritidis str. 607307-6 | |
| SRR518860 | Salmonella enterica subsp. enterica serovar Enteritidis str. CVM_81-2490 | |
| SRR518935 | Salmonella enterica subsp. enterica serovar Enteritidis str. CDC_2010K_0956 | |
| SRR518936 | Salmonella enterica subsp. enterica serovar Enteritidis str. CDC_2010K_0956 | |
| SRR520411 | Salmonella enterica subsp. enterica serovar Enteritidis str. ATCC BAA-1587 | |
| SRR520414 | Salmonella enterica subsp. enterica serovar Enteritidis str. ATCC BAA-1587 | |
| SRR520417 | Salmonella enterica subsp. enterica serovar Enteritidis str. ATCC BAA-1587 | |
| SRR520420 | Salmonella enterica subsp. enterica serovar Enteritidis str. ATCC BAA-1587 | |
| SRR520429 | Salmonella enterica subsp. enterica serovar Enteritidis str. ATCC BAA-1587 | |
| SRR520436 | Salmonella enterica subsp. enterica serovar Enteritidis str. ATCC BAA-1587 | |
| SRR5232056 | Salmonella enterica subsp. enterica serovar Enteritidis | |
| SRR5232061 | Salmonella enterica subsp. enterica serovar Enteritidis | |
| SRR5232062 | Salmonella enterica subsp. enterica serovar Enteritidis | |
| SRR5232080 | Salmonella enterica subsp. enterica serovar Enteritidis | |
| SRR5232081 | Salmonella enterica subsp. enterica serovar Enteritidis | |
| SRR5232082 | Salmonella enterica subsp. enterica serovar Enteritidis | |
| SRR5232083 | Salmonella enterica subsp. enterica serovar Enteritidis | |
| SRR5232084 | Salmonella enterica subsp. enterica serovar Enteritidis | |
| SRR5232085 | Salmonella enterica subsp. enterica serovar Enteritidis | |
| SRR5341556 | Salmonella enterica subsp. enterica serovar Enteritidis | |
| SRR5381280 | Salmonella enterica subsp. enterica serovar Enteritidis | |
| SRR5481459 | Salmonella enterica subsp. enterica serovar Enteritidis | |
| SRR5481460 | Salmonella enterica subsp. enterica serovar Enteritidis | |
| SRR5481461 | Salmonella enterica subsp. enterica serovar Enteritidis | |
| SRR5481462 | Salmonella enterica subsp. enterica serovar Enteritidis | |
| SRR5601734 | Salmonella enterica subsp. enterica serovar Enteritidis str. UC05 | |
| SRR5649661 | Salmonella enterica subsp. enterica serovar Enteritidis str. CFSAN000044 | |
| SRR5649766 | Salmonella enterica subsp. enterica serovar Enteritidis str. CFSAN000060 | |
| SRR5656389 | Salmonella enterica subsp. enterica serovar Enteritidis str. CFSAN000035 | |
| SRR5656392 | Salmonella enterica subsp. enterica serovar Enteritidis str. CFSAN000040 | |
| SRR5656420 | Salmonella enterica subsp. enterica serovar Enteritidis str. CFSAN000036 | |
| SRR5656423 | Salmonella enterica subsp. enterica serovar Enteritidis str. CFSAN000054 | |
| SRR5656424 | Salmonella enterica subsp. enterica serovar Enteritidis str. CFSAN000053 | |
| SRR5656425 | Salmonella enterica subsp. enterica serovar Enteritidis str. CFSAN000052 | |
| SRR5819637 | Salmonella enterica subsp. enterica serovar Enteritidis | |
| SRR5819643 | Salmonella enterica subsp. enterica serovar Enteritidis | |
| SRR5819786 | Salmonella enterica subsp. enterica serovar Enteritidis | |
| SRR5819791 | Salmonella enterica subsp. enterica serovar Enteritidis | |
| SRR5950245 | Salmonella enterica subsp. enterica serovar Enteritidis | |
| SRR5950483 | Salmonella enterica subsp. enterica serovar Enteritidis | |
| SRR5950825 | Salmonella enterica subsp. enterica serovar Enteritidis | |
| SRR5951329 | Salmonella enterica subsp. enterica serovar Enteritidis | |
| SRR5951392 | Salmonella enterica subsp. enterica serovar Enteritidis | |
| SRR6038563 | Salmonella enterica subsp. enterica serovar Enteritidis str. 629163 | |
| SRR6038565 | Salmonella enterica subsp. enterica serovar Enteritidis str. 629164-37 | |
| SRR6038568 | Salmonella enterica subsp. enterica serovar Enteritidis str. 485549-17 | |
| SRR6038569 | Salmonella enterica subsp. enterica serovar Enteritidis str. 629164-26 | |
| SRR610557 | Salmonella enterica subsp. enterica serovar Enteritidis str. 10-29153 | |
| SRR610567 | Salmonella enterica subsp. enterica serovar Enteritidis str. 10-30147 | |
| SRR610679 | Salmonella enterica subsp. enterica serovar Enteritidis str. 10-31528 | |
| SRR610680 | Salmonella enterica subsp. enterica serovar Enteritidis str. 10-33213 | |
| SRR610681 | Salmonella enterica subsp. enterica serovar Enteritidis str. 10-33369 | |
| SRR610733 | Salmonella enterica subsp. enterica serovar Enteritidis str. 10-33371 | |
| SRR6107963 | Salmonella enterica subsp. enterica serovar Enteritidis | |
| SRR6108680 | Salmonella enterica subsp. enterica serovar Enteritidis | |
| SRR6108693 | Salmonella enterica subsp. enterica serovar Enteritidis | |
| SRR611123 | Salmonella enterica subsp. enterica serovar Enteritidis str. 10-34587 | |
| SRR611124 | Salmonella enterica subsp. enterica serovar Enteritidis str. 10-34599 | |
| SRR611125 | Salmonella enterica subsp. enterica serovar Enteritidis str. 10-34601 | |
| SRR611126 | Salmonella enterica subsp. enterica serovar Enteritidis str. 10-35178 | |
| SRR611127 | Salmonella enterica subsp. enterica serovar Enteritidis str. 10-35179 | |
| SRR611128 | Salmonella enterica subsp. enterica serovar Enteritidis str. 10-35180 | |
| SRR611280 | Salmonella enterica subsp. enterica serovar Enteritidis str. 10-35181 | |
| SRR611281 | Salmonella enterica subsp. enterica serovar Enteritidis str. 10-35182 | |
| SRR611282 | Salmonella enterica subsp. enterica serovar Enteritidis str. 10-35183 | |
| SRR611283 | Salmonella enterica subsp. enterica serovar Enteritidis str. 10-35417 | |
| SRR611284 | Salmonella enterica subsp. enterica serovar Enteritidis str. 10-36119 | |
| SRR611285 | Salmonella enterica subsp. enterica serovar Enteritidis str. 10-36319 | |
| SRR6126768 | Salmonella enterica subsp. enterica serovar Enteritidis str. CFSAN000034 | |
| SRR6126854 | Salmonella enterica subsp. enterica serovar Enteritidis str. CFSAN000059 | |
| SRR6179224 | Salmonella enterica subsp. enterica serovar Enteritidis | |
| SRR6179266 | Salmonella enterica subsp. enterica serovar Enteritidis | |
| SRR6179271 | Salmonella enterica subsp. enterica serovar Enteritidis | |
| SRR6179272 | Salmonella enterica subsp. enterica serovar Enteritidis | |
| SRR6179273 | Salmonella enterica subsp. enterica serovar Enteritidis | |
| SRR6179274 | Salmonella enterica subsp. enterica serovar Enteritidis | |
| SRR618448 | Salmonella enterica subsp. enterica serovar Enteritidis str. 10-28670 | |
| SRR618449 | Salmonella enterica subsp. enterica serovar Enteritidis str. 10-29949 | |
| SRR618450 | Salmonella enterica subsp. enterica serovar Enteritidis str. 10-33603 | |
| SRR618451 | Salmonella enterica subsp. enterica serovar Enteritidis str. 10-34213 | |
| SRR618454 | Salmonella enterica subsp. enterica serovar Enteritidis str. 10-35184 | |
| SRR618456 | Salmonella enterica subsp. enterica serovar Enteritidis str. 10-36979 | |
| SRR618458 | Salmonella enterica subsp. enterica serovar Enteritidis str. 10-37723 | |
| SRR618459 | Salmonella enterica subsp. enterica serovar Enteritidis str. 10-38792 | |
| SRR618462 | Salmonella enterica subsp. enterica serovar Enteritidis str. 10-39087 | |
| SRR618464 | Salmonella enterica subsp. enterica serovar Enteritidis str. 11-03844 | |
| SRR618465 | Salmonella enterica subsp. enterica serovar Enteritidis str. 11-06235 | |
| SRR618468 | Salmonella enterica subsp. enterica serovar Enteritidis str. 11-21079 | |
| SRR618469 | Salmonella enterica subsp. enterica serovar Enteritidis str. 11-22186 | |
| SRR618470 | Salmonella enterica subsp. enterica serovar Enteritidis str. 11-27690 | |
| SRR618471 | Salmonella enterica subsp. enterica serovar Enteritidis str. 11-30508 | |
| SRR618472 | Salmonella enterica subsp. enterica serovar Enteritidis str. 11-31312 | |
| SRR618473 | Salmonella enterica subsp. enterica serovar Enteritidis str. 11-32014 | |
| SRR6214393 | Salmonella enterica subsp. enterica serovar Enteritidis | |
| SRR6214444 | Salmonella enterica subsp. enterica serovar Enteritidis | |
| SRR6309792 | Salmonella enterica subsp. enterica serovar Enteritidis | |
| SRR6311926 | Salmonella enterica subsp. enterica serovar Enteritidis | |
| SRR6311928 | Salmonella enterica subsp. enterica serovar Enteritidis | |
| SRR6312168 | Salmonella enterica subsp. enterica serovar Enteritidis | |
| SRR6377030 | Salmonella enterica subsp. enterica serovar Enteritidis | |
| SRR6377031 | Salmonella enterica subsp. enterica serovar Enteritidis | |
| SRR6377033 | Salmonella enterica subsp. enterica serovar Enteritidis | |
| SRR6377123 | Salmonella enterica subsp. enterica serovar Enteritidis | |
| SRR6377124 | Salmonella enterica subsp. enterica serovar Enteritidis | |
| SRR6377127 | Salmonella enterica subsp. enterica serovar Enteritidis | |
| SRR6377132 | Salmonella enterica subsp. enterica serovar Enteritidis | |
| SRR6377133 | Salmonella enterica subsp. enterica serovar Enteritidis | |
| SRR6377154 | Salmonella enterica subsp. enterica serovar Enteritidis | |
| SRR6411456 | Salmonella enterica subsp. enterica serovar Enteritidis | |
| SRR6411457 | Salmonella enterica subsp. enterica serovar Enteritidis | |
| SRR6411478 | Salmonella enterica subsp. enterica serovar Enteritidis | |
| SRR6411492 | Salmonella enterica subsp. enterica serovar Enteritidis | |
| SRR6411494 | Salmonella enterica subsp. enterica serovar Enteritidis | |
| SRR6412209 | Salmonella enterica subsp. enterica serovar Enteritidis | |
| SRR6412210 | Salmonella enterica subsp. enterica serovar Enteritidis | |
| SRR6412217 | Salmonella enterica subsp. enterica serovar Enteritidis | |
| SRR6412220 | Salmonella enterica subsp. enterica serovar Enteritidis | |
| SRR6466198 | Salmonella enterica subsp. enterica serovar Enteritidis | |
| SRR6466285 | Salmonella enterica subsp. enterica serovar Enteritidis | |
| SRR6466288 | Salmonella enterica subsp. enterica serovar Enteritidis | |
| SRR6466289 | Salmonella enterica subsp. enterica serovar Enteritidis | |
| SRR6466292 | Salmonella enterica subsp. enterica serovar Enteritidis | |
| SRR6466293 | Salmonella enterica subsp. enterica serovar Enteritidis | |
| SRR6466358 | Salmonella enterica subsp. enterica serovar Enteritidis | |
| SRR6466360 | Salmonella enterica subsp. enterica serovar Enteritidis | |
| SRR6466361 | Salmonella enterica subsp. enterica serovar Enteritidis | |
| SRR6491040 | Salmonella enterica subsp. enterica serovar Enteritidis | |
| SRR6491046 | Salmonella enterica subsp. enterica serovar Enteritidis | |
| SRR6491187 | Salmonella enterica subsp. enterica serovar Enteritidis | |
| SRR6498587 | Salmonella enterica subsp. enterica serovar Enteritidis | |
| SRR651129 | Salmonella enterica subsp. enterica serovar Enteritidis str. 12-14426 | |
| SRR651130 | Salmonella enterica subsp. enterica serovar Enteritidis str. 12-14693 | |
| SRR651131 | Salmonella enterica subsp. enterica serovar Enteritidis str. 12-14700 | |
| SRR651153 | Salmonella enterica subsp. enterica serovar Enteritidis str. 12-14703 | |
| SRR651154 | Salmonella enterica subsp. enterica serovar Enteritidis str. 12-14895 | |
| SRR651155 | Salmonella enterica subsp. enterica serovar Enteritidis str. 12-16076 | |
| SRR651156 | Salmonella enterica subsp. enterica serovar Enteritidis str. 12-16086 | |
| SRR651157 | Salmonella enterica subsp. enterica serovar Enteritidis str. 12-17240 | |
| SRR651158 | Salmonella enterica subsp. enterica serovar Enteritidis str. 12-17486 | |
| SRR651159 | Salmonella enterica subsp. enterica serovar Enteritidis str. 12-17892 | |
| SRR651160 | Salmonella enterica subsp. enterica serovar Enteritidis str. 12-17893 | |
| SRR651161 | Salmonella enterica subsp. enterica serovar Enteritidis str. 12-18137 | |
| SRR651162 | Salmonella enterica subsp. enterica serovar Enteritidis str. 12-18160 | |
| SRR651164 | Salmonella enterica subsp. enterica serovar Enteritidis str. 12-18401 | |
| SRR651165 | Salmonella enterica subsp. enterica serovar Enteritidis str. 12-19490 | |
| SRR651166 | Salmonella enterica subsp. enterica serovar Enteritidis str. 12-19798 | |
| SRR651168 | Salmonella enterica subsp. enterica serovar Enteritidis str. 12-19824 | |
| SRR651169 | Salmonella enterica subsp. enterica serovar Enteritidis str. 12-20008 | |
| SRR651171 | Salmonella enterica subsp. enterica serovar Enteritidis str. 12-20418 | |
| SRR651173 | Salmonella enterica subsp. enterica serovar Enteritidis str. 12-21190 | |
| SRR651174 | Salmonella enterica subsp. enterica serovar Enteritidis str. 12-21313 | |
| SRR651175 | Salmonella enterica subsp. enterica serovar Enteritidis str. 12-21314 | |
| SRR651176 | Salmonella enterica subsp. enterica serovar Enteritidis str. 12-21569 | |
| SRR651177 | Salmonella enterica subsp. enterica serovar Enteritidis str. 12-21687 | |
| SRR651184 | Salmonella enterica subsp. enterica serovar Enteritidis str. 12-18138 | |
| SRR651966 | Salmonella enterica subsp. enterica serovar Enteritidis str. 12-11922 | |
| SRR651967 | Salmonella enterica subsp. enterica serovar Enteritidis str. 12-12016 | |
| SRR651968 | Salmonella enterica subsp. enterica serovar Enteritidis str. 12-12071 | |
| SRR651969 | Salmonella enterica subsp. enterica serovar Enteritidis str. 12-12205 | |
| SRR651970 | Salmonella enterica subsp. enterica serovar Enteritidis str. 12-12288 | |
| SRR651972 | Salmonella enterica subsp. enterica serovar Enteritidis str. 12-14089 | |
| SRR651974 | Salmonella enterica subsp. enterica serovar Enteritidis str. 12-14487 | |
| SRR651975 | Salmonella enterica subsp. enterica serovar Enteritidis str. 12-15432 | |
| SRR651976 | Salmonella enterica subsp. enterica serovar Enteritidis str. 12-15721 | |
| SRR651977 | Salmonella enterica subsp. enterica serovar Enteritidis str. 12-16414 | |
| SRR651993 | Salmonella enterica subsp. enterica serovar Enteritidis str. 12-16608 | |
| SRR651994 | Salmonella enterica subsp. enterica serovar Enteritidis str. 12-17211 | |
| SRR652067 | Salmonella enterica subsp. enterica serovar Enteritidis str. 12-18526 | |
| SRR652068 | Salmonella enterica subsp. enterica serovar Enteritidis str. 12-18775 | |
| SRR652069 | Salmonella enterica subsp. enterica serovar Enteritidis str. 12-19760 | |
| SRR652070 | Salmonella enterica subsp. enterica serovar Enteritidis str. 12-20343 | |
| SRR652071 | Salmonella enterica subsp. enterica serovar Enteritidis str. 12-21567 | |
| SRR652073 | Salmonella enterica subsp. enterica serovar Enteritidis str. 12-22120 | |
| SRR652074 | Salmonella enterica subsp. enterica serovar Enteritidis str. 12-22891 | |
| SRR652075 | Salmonella enterica subsp. enterica serovar Enteritidis str. 12-22983 | |
| SRR652076 | Salmonella enterica subsp. enterica serovar Enteritidis str. 12-23418 | |
| SRR652077 | Salmonella enterica subsp. enterica serovar Enteritidis str. 12-23426 | |
| SRR652078 | Salmonella enterica subsp. enterica serovar Enteritidis str. 12-24078 | |
| SRR652079 | Salmonella enterica subsp. enterica serovar Enteritidis str. 12-24683 | |
| SRR652080 | Salmonella enterica subsp. enterica serovar Enteritidis str. 12-24729 | |
| SRR652081 | Salmonella enterica subsp. enterica serovar Enteritidis str. 12-25457 | |
| SRR652082 | Salmonella enterica subsp. enterica serovar Enteritidis str. 12-26550 | |
| SRR652083 | Salmonella enterica subsp. enterica serovar Enteritidis str. 12-26681 | |
| SRR652084 | Salmonella enterica subsp. enterica serovar Enteritidis str. 12-26778 | |
| SRR652085 | Salmonella enterica subsp. enterica serovar Enteritidis str. 12-26898 | |
| SRR653600 | Salmonella enterica subsp. enterica serovar Enteritidis str. 12-14697 | |
| SRR653601 | Salmonella enterica subsp. enterica serovar Enteritidis str. 12-14699 | |
| SRR653603 | Salmonella enterica subsp. enterica serovar Enteritidis str. 12-14982 | |
| SRR6667875 | Salmonella enterica subsp. enterica serovar Enteritidis | |
| SRR6668033 | Salmonella enterica subsp. enterica serovar Enteritidis | |
|  |  | |
| ***Salmonella enterica subsp. enterica* serovar Dublin** | | |
| **Accession number** | | **Scientific name** |
| ERR015586 | | Salmonella enterica subsp. enterica serovar Dublin |
| ERR015591 | | Salmonella enterica subsp. enterica serovar Dublin |
| ERR015592 | | Salmonella enterica subsp. enterica serovar Dublin |
| ERR015593 | | Salmonella enterica subsp. enterica serovar Dublin |
| ERR015595 | | Salmonella enterica subsp. enterica serovar Dublin |
| ERR025162 | | Salmonella enterica subsp. enterica serovar Dublin |
| ERR025163 | | Salmonella enterica subsp. enterica serovar Dublin |
| ERR025167 | | Salmonella enterica subsp. enterica serovar Dublin |
| ERR025168 | | Salmonella enterica subsp. enterica serovar Dublin |
| ERR025169 | | Salmonella enterica subsp. enterica serovar Dublin |
| ERR025170 | | Salmonella enterica subsp. enterica serovar Dublin |
| ERR025171 | | Salmonella enterica subsp. enterica serovar Dublin |
| ERR036125 | | Salmonella enterica subsp. enterica serovar Dublin |
| ERR036126 | | Salmonella enterica subsp. enterica serovar Dublin |
| ERR036127 | | Salmonella enterica subsp. enterica serovar Dublin |
| ERR036128 | | Salmonella enterica subsp. enterica serovar Dublin |
| ERR036129 | | Salmonella enterica subsp. enterica serovar Dublin |
| ERR036130 | | Salmonella enterica subsp. enterica serovar Dublin |
| ERR036131 | | Salmonella enterica subsp. enterica serovar Dublin |
| ERR038343 | | Salmonella enterica subsp. enterica serovar Dublin |
| ERR038344 | | Salmonella enterica subsp. enterica serovar Dublin |
| ERR1010001 | | Salmonella enterica subsp. enterica serovar Dublin |
| ERR1010029 | | Salmonella enterica subsp. enterica serovar Dublin |
| ERR1010030 | | Salmonella enterica subsp. enterica serovar Dublin |
| ERR1010037 | | Salmonella enterica subsp. enterica serovar Dublin |
| ERR1010056 | | Salmonella enterica subsp. enterica serovar Dublin |
| ERR1010061 | | Salmonella enterica subsp. enterica serovar Dublin |
| ERR1010062 | | Salmonella enterica subsp. enterica serovar Dublin |
| ERR1010078 | | Salmonella enterica subsp. enterica serovar Dublin |
| ERR1010083 | | Salmonella enterica subsp. enterica serovar Dublin |
| ERR1010109 | | Salmonella enterica subsp. enterica serovar Dublin |
| ERR1010168 | | Salmonella enterica subsp. enterica serovar Dublin |
| ERR1201706 | | Salmonella enterica subsp. enterica serovar Dublin |
| ERR1201707 | | Salmonella enterica subsp. enterica serovar Dublin |
| ERR1201708 | | Salmonella enterica subsp. enterica serovar Dublin |
| ERR1201709 | | Salmonella enterica subsp. enterica serovar Dublin |
| ERR1201710 | | Salmonella enterica subsp. enterica serovar Dublin |
| ERR1201711 | | Salmonella enterica subsp. enterica serovar Dublin |
| ERR1201712 | | Salmonella enterica subsp. enterica serovar Dublin |
| ERR1201713 | | Salmonella enterica subsp. enterica serovar Dublin |
| ERR1201714 | | Salmonella enterica subsp. enterica serovar Dublin |
| ERR1201715 | | Salmonella enterica subsp. enterica serovar Dublin |
| ERR1201716 | | Salmonella enterica subsp. enterica serovar Dublin |
| ERR1201717 | | Salmonella enterica subsp. enterica serovar Dublin |
| ERR1201718 | | Salmonella enterica subsp. enterica serovar Dublin |
| ERR1201719 | | Salmonella enterica subsp. enterica serovar Dublin |
| ERR1201720 | | Salmonella enterica subsp. enterica serovar Dublin |
| ERR1201721 | | Salmonella enterica subsp. enterica serovar Dublin |
| ERR1201722 | | Salmonella enterica subsp. enterica serovar Dublin |
| ERR1201723 | | Salmonella enterica subsp. enterica serovar Dublin |
| ERR1201724 | | Salmonella enterica subsp. enterica serovar Dublin |
| ERR1201725 | | Salmonella enterica subsp. enterica serovar Dublin |
| ERR1201726 | | Salmonella enterica subsp. enterica serovar Dublin |
| ERR1201727 | | Salmonella enterica subsp. enterica serovar Dublin |
| ERR1201728 | | Salmonella enterica subsp. enterica serovar Dublin |
| ERR1201729 | | Salmonella enterica subsp. enterica serovar Dublin |
| ERR1201730 | | Salmonella enterica subsp. enterica serovar Dublin |
| ERR1201731 | | Salmonella enterica subsp. enterica serovar Dublin |
| ERR1201732 | | Salmonella enterica subsp. enterica serovar Dublin |
| ERR1201733 | | Salmonella enterica subsp. enterica serovar Dublin |
| ERR1706739 | | Salmonella enterica subsp. enterica serovar Dublin |
| ERR1706740 | | Salmonella enterica subsp. enterica serovar Dublin |
| ERR1706745 | | Salmonella enterica subsp. enterica serovar Dublin |
| ERR1706746 | | Salmonella enterica subsp. enterica serovar Dublin |
| ERR1706757 | | Salmonella enterica subsp. enterica serovar Dublin |
| ERR1706761 | | Salmonella enterica subsp. enterica serovar Dublin |
| ERR1706764 | | Salmonella enterica subsp. enterica serovar Dublin |
| ERR1706766 | | Salmonella enterica subsp. enterica serovar Dublin |
| ERR1706767 | | Salmonella enterica subsp. enterica serovar Dublin |
| ERR1706769 | | Salmonella enterica subsp. enterica serovar Dublin |
| ERR1828947 | | Salmonella enterica subsp. enterica serovar Dublin |
| ERR1837575 | | Salmonella enterica subsp. enterica serovar Dublin |
| ERR2173653 | | Salmonella enterica subsp. enterica serovar Dublin |
| ERR226476 | | Salmonella enterica subsp. enterica serovar Dublin |
| ERR226479 | | Salmonella enterica subsp. enterica serovar Dublin |
| ERR230468 | | Salmonella enterica subsp. enterica serovar Dublin |
| ERR351247 | | Salmonella enterica subsp. enterica serovar Dublin str. CT_02021853 |
| ERR424895 | | Salmonella enterica subsp. enterica serovar Dublin |
| ERR424896 | | Salmonella enterica subsp. enterica serovar Dublin |
| ERR424907 | | Salmonella enterica subsp. enterica serovar Dublin |
| ERR424911 | | Salmonella enterica subsp. enterica serovar Dublin |
| ERR433181 | | Salmonella enterica subsp. enterica serovar Dublin |
| ERR433182 | | Salmonella enterica subsp. enterica serovar Dublin |
| ERR433183 | | Salmonella enterica subsp. enterica serovar Dublin |
| ERR472736 | | Salmonella enterica subsp. enterica serovar Dublin |
| ERR472737 | | Salmonella enterica subsp. enterica serovar Dublin |
| ERR587446 | | Salmonella enterica subsp. enterica serovar Dublin |
| ERR587447 | | Salmonella enterica subsp. enterica serovar Dublin |
| ERR587448 | | Salmonella enterica subsp. enterica serovar Dublin |
| ERR587449 | | Salmonella enterica subsp. enterica serovar Dublin |
| ERR587450 | | Salmonella enterica subsp. enterica serovar Dublin |
| ERR587451 | | Salmonella enterica subsp. enterica serovar Dublin |
| ERR587452 | | Salmonella enterica subsp. enterica serovar Dublin |
| ERR587453 | | Salmonella enterica subsp. enterica serovar Dublin |
| ERR587454 | | Salmonella enterica subsp. enterica serovar Dublin |
| ERR587455 | | Salmonella enterica subsp. enterica serovar Dublin |
| ERR587456 | | Salmonella enterica subsp. enterica serovar Dublin |
| ERR587457 | | Salmonella enterica subsp. enterica serovar Dublin |
| ERR587458 | | Salmonella enterica subsp. enterica serovar Dublin |
| ERR587459 | | Salmonella enterica subsp. enterica serovar Dublin |
| ERR587460 | | Salmonella enterica subsp. enterica serovar Dublin |
| ERR587461 | | Salmonella enterica subsp. enterica serovar Dublin |
| ERR587462 | | Salmonella enterica subsp. enterica serovar Dublin |
| ERR587463 | | Salmonella enterica subsp. enterica serovar Dublin |
| ERR587464 | | Salmonella enterica subsp. enterica serovar Dublin |
| ERR587465 | | Salmonella enterica subsp. enterica serovar Dublin |
| ERR587466 | | Salmonella enterica subsp. enterica serovar Dublin |
| ERR587467 | | Salmonella enterica subsp. enterica serovar Dublin |
| ERR587468 | | Salmonella enterica subsp. enterica serovar Dublin |
| ERR587469 | | Salmonella enterica subsp. enterica serovar Dublin |
| ERR587470 | | Salmonella enterica subsp. enterica serovar Dublin |
| ERR791765 | | Salmonella enterica subsp. enterica serovar Dublin |
| ERR984718 | | Salmonella enterica subsp. enterica serovar Dublin |
| ERR984728 | | Salmonella enterica subsp. enterica serovar Dublin |
| ERR984730 | | Salmonella enterica subsp. enterica serovar Dublin |
| ERR984733 | | Salmonella enterica subsp. enterica serovar Dublin |
| ERR984734 | | Salmonella enterica subsp. enterica serovar Dublin |
| ERR984786 | | Salmonella enterica subsp. enterica serovar Dublin |
| ERR984809 | | Salmonella enterica subsp. enterica serovar Dublin |
| ERR984811 | | Salmonella enterica subsp. enterica serovar Dublin |
| ERR984815 | | Salmonella enterica subsp. enterica serovar Dublin |
| ERR984819 | | Salmonella enterica subsp. enterica serovar Dublin |
| ERR984849 | | Salmonella enterica subsp. enterica serovar Dublin |
| ERR984883 | | Salmonella enterica subsp. enterica serovar Dublin |
| ERR984916 | | Salmonella enterica subsp. enterica serovar Dublin |
| ERR984922 | | Salmonella enterica subsp. enterica serovar Dublin |
| ERR984943 | | Salmonella enterica subsp. enterica serovar Dublin |
| ERR998469 | | Salmonella enterica subsp. enterica serovar Dublin |
| ERR998477 | | Salmonella enterica subsp. enterica serovar Dublin |
| ERR998558 | | Salmonella enterica subsp. enterica serovar Dublin |
| ERR998607 | | Salmonella enterica subsp. enterica serovar Dublin |
| ERR998620 | | Salmonella enterica subsp. enterica serovar Dublin |
| SRR1106394 | | Salmonella enterica subsp. enterica serovar Dublin |
| SRR1106503 | | Salmonella enterica subsp. enterica serovar Dublin |
| SRR1106504 | | Salmonella enterica subsp. enterica serovar Dublin |
| SRR1118717 | | Salmonella enterica subsp. enterica serovar Dublin |
| SRR1118718 | | Salmonella enterica subsp. enterica serovar Dublin |
| SRR1122493 | | Salmonella enterica subsp. enterica serovar Dublin |
| SRR1122494 | | Salmonella enterica subsp. enterica serovar Dublin |
| SRR1122706 | | Salmonella enterica subsp. enterica serovar Dublin |
| SRR1122707 | | Salmonella enterica subsp. enterica serovar Dublin |
| SRR1122708 | | Salmonella enterica subsp. enterica serovar Dublin |
| SRR1133229 | | Salmonella enterica subsp. enterica serovar Dublin |
| SRR1172020 | | Salmonella enterica subsp. enterica serovar Dublin |
| SRR1840709 | | Salmonella enterica subsp. enterica serovar Dublin |
| SRR1840710 | | Salmonella enterica subsp. enterica serovar Dublin |
| SRR1840717 | | Salmonella enterica subsp. enterica serovar Dublin |
| SRR1976179 | | Salmonella enterica subsp. enterica serovar Dublin str. SA20093032 |
| SRR1976180 | | Salmonella enterica subsp. enterica serovar Dublin str. SA20093032 |
| SRR2566988 | | Salmonella enterica subsp. enterica serovar Dublin |
| SRR2567125 | | Salmonella enterica subsp. enterica serovar Dublin |
| SRR2567128 | | Salmonella enterica subsp. enterica serovar Dublin |
| SRR2567144 | | Salmonella enterica subsp. enterica serovar Dublin |
| SRR2567191 | | Salmonella enterica subsp. enterica serovar Dublin |
| SRR3097384 | | Salmonella enterica subsp. enterica serovar Dublin |
| SRR3097404 | | Salmonella enterica subsp. enterica serovar Dublin |
| SRR3097409 | | Salmonella enterica subsp. enterica serovar Dublin |
| SRR3097419 | | Salmonella enterica subsp. enterica serovar Dublin |
| SRR3097426 | | Salmonella enterica subsp. enterica serovar Dublin |
| SRR3097435 | | Salmonella enterica subsp. enterica serovar Dublin |
| SRR3097449 | | Salmonella enterica subsp. enterica serovar Dublin |
| SRR3097456 | | Salmonella enterica subsp. enterica serovar Dublin |
| SRR3097505 | | Salmonella enterica subsp. enterica serovar Dublin |
| SRR3097507 | | Salmonella enterica subsp. enterica serovar Dublin |
| SRR3097518 | | Salmonella enterica subsp. enterica serovar Dublin |
| SRR3097528 | | Salmonella enterica subsp. enterica serovar Dublin |
| SRR3097533 | | Salmonella enterica subsp. enterica serovar Dublin |
| SRR3097565 | | Salmonella enterica subsp. enterica serovar Dublin |
| SRR3664744 | | Salmonella enterica subsp. enterica serovar Dublin |
| SRR3664802 | | Salmonella enterica subsp. enterica serovar Dublin |
| SRR3664934 | | Salmonella enterica subsp. enterica serovar Dublin |
| SRR3664944 | | Salmonella enterica subsp. enterica serovar Dublin |
| SRR444607 | | Salmonella enterica subsp. enterica serovar Dublin str. SL1438 |
| SRR444610 | | Salmonella enterica subsp. enterica serovar Dublin |
| SRR444973 | | Salmonella enterica subsp. enterica serovar Dublin str. SL1438 |
| SRR5155659 | | Salmonella enterica subsp. enterica serovar Dublin str. 0773 |
| SRR5498104 | | Salmonella enterica subsp. enterica serovar Dublin str. CFSAN000516 |
| SRR5498105 | | Salmonella enterica subsp. enterica serovar Dublin str. CFSAN000517 |
| SRR5601759 | | Salmonella enterica subsp. enterica serovar Dublin str. UC04 |
| SRR5627247 | | Salmonella enterica subsp. enterica serovar Dublin |
| SRR5985919 | | Salmonella enterica subsp. enterica serovar Dublin |
| SRR5985920 | | Salmonella enterica subsp. enterica serovar Dublin |
| SRR6000478 | | Salmonella enterica subsp. enterica serovar Dublin |
| SRR6160993 | | Salmonella enterica subsp. enterica serovar Dublin |
| SRR6278424 | | Salmonella enterica subsp. enterica serovar Dublin |
| SRR6278772 | | Salmonella enterica subsp. enterica serovar Dublin |
| SRR6291058 | | Salmonella enterica subsp. enterica serovar Dublin |
| SRR6291064 | | Salmonella enterica subsp. enterica serovar Dublin |
| SRR6291070 | | Salmonella enterica subsp. enterica serovar Dublin |
| SRR6291075 | | Salmonella enterica subsp. enterica serovar Dublin |
| SRR6291076 | | Salmonella enterica subsp. enterica serovar Dublin |
| SRR6308366 | | Salmonella enterica subsp. enterica serovar Dublin |
| SRR6308559 | | Salmonella enterica subsp. enterica serovar Dublin |
| SRR6310053 | | Salmonella enterica subsp. enterica serovar Dublin |
| SRR6311216 | | Salmonella enterica subsp. enterica serovar Dublin |
| SRR6311937 | | Salmonella enterica subsp. enterica serovar Dublin |
| SRR6312043 | | Salmonella enterica subsp. enterica serovar Dublin |
| SRR6321910 | | Salmonella enterica subsp. enterica serovar Dublin |
| SRR6475628 | | Salmonella enterica subsp. enterica serovar Dublin |
| SRR6475644 | | Salmonella enterica subsp. enterica serovar Dublin |
| SRR6502082 | | Salmonella enterica subsp. enterica serovar Dublin |
