# Supplementary material for: The O-Antigen Epitope Governs Susceptibility to Colistin in Salmonella enterica
Source: mBio. 2020 Jan 28;11(1):e02831-19. doi: 10.1128/mBio.02831-19 (PMC6989106; doi:10.1128/mBio.02831-19)
Supplement: TABLE S3 [file mBio.02831-19-st003.docx]

**Supplementary Table 3.** **Mutations identified in the genome-sequenced** *Salmonella* Enteritidis *S02703-14* **isolate**

| **Chromosome Position** | **Type of mutation** | **Reference** | **Consensus** | **Amino acid change** | **Effect** | **Product** | | | |  |  |  |
| --- | --- | --- | --- | --- | --- | --- | --- | --- | --- | --- | --- | --- |
| 106408 | snp | G | T | T2051K | missense_variant | | T1SS secreted agglutinin RTX |  |  | |  |  |
| 235940 | snp | A | G | V35A | missense_variant | | Periplasmic fimbrial chaperone StfD |  |  | |  |  |
| 323278 | snp | A | G | N59D | missense_variant | | Outer membrane fimbrial usher protein |  |  | | |  |
| 1818500 | del | TCT | TT | S152fs | frameshift_variant | | O-antigen polymerase |  |  | |  |  |
| 2631101 | snp | A | G | D826G | missense_variant | | AIDA autotransporter-like protein |  |  | |  |  |
| 2631107 | mnp | AG | GA | S828D | missense_variant | | AIDA autotransporter-like protein |  |  | |  |  |
| 2631164 | snp | T | C | L847S | missense_variant | | AIDA autotransporter-like protein |  |  | |  |  |

snp, single nucleotide polymorphism; mnp, multi nucleotide polymorphism; del, deletion; fs, frameshift
